# Quantifying diversity and growth form distribution of scleractinian corals, in Mangrove Bay, El Qoseir, Egypt

**DOI:** 10.3897/BDJ.13.e134282

**Published:** 2025-01-23

**Authors:** Theres Koch, Angelina Ivkić, Lewis A Jones, Victor S Scharnhorst, Constanze M Stix, Antonia Auer, Theda Schöchtner, Gözde Özer, Simon Steinwender, Joseph Wallace Daurella, Martin Zuschin

**Affiliations:** 1 Department of Functional and Evolutionary Ecology, University of Vienna, Djerassiplatz 1, 1030 Vienna, Austria Department of Functional and Evolutionary Ecology, University of Vienna Djerassiplatz 1, 1030 Vienna Austria; 2 Department of Paleoentology, University of Vienna, Josef-Holaubek-Platz 2, 1090 Vienna, Austria Department of Paleoentology, University of Vienna Josef-Holaubek-Platz 2, 1090 Vienna Austria; 3 Department of Earth Sciences, University College London, Gower Street, London, WC1E 6BT, United Kingdom Department of Earth Sciences, University College London Gower Street, London, WC1E 6BT United Kingdom; 4 Institute of Integrative Nature Conservation Research (INF), University of Natural Resources and Life Sciences, Gregor-Mendel-Straße 33, 1180 Vienna, Austria Institute of Integrative Nature Conservation Research (INF), University of Natural Resources and Life Sciences Gregor-Mendel-Straße 33, 1180 Vienna Austria; 5 Department of Biology, University of York, Wentworth Way, York, YO10 5DD, United Kingdom Department of Biology, University of York Wentworth Way, York, YO10 5DD United Kingdom

**Keywords:** coral reefs, Anthozoa, Scleractinian, northern Red Sea, coral community composition, Line Intercept Transect (LIT), coral biodiversity, coral cover

## Abstract

**Background:**

Coral reefs provide habitat for approximately 25% of all extant marine species, including 845 species of scleractinian corals. These rich ecosystems are becoming increasingly degraded in many regions by a range of anthropogenic factors, including recurrent bleaching episodes associated with rising sea surface temperatures. Within the northern Red Sea, coral reef communities appear to be faring relatively better than global trends and continue to exhibit remarkable diversity and thermal tolerance. However, recent reports of coral bleaching in the region highlight the urgent need for comprehensive ecological surveys to provide a baseline for long-term monitoring of biodiversity and potential species loss. This field report aims to support the tracking of diversity and growth form distributions of scleractinian corals at two reef sites at Mangrove Bay, El Qoseir, Egypt. Over time, it is our hope that such reports will contribute to broader databases and analyses focused on the biodiversity of reef-building coral species in the northern Red Sea.

**New information:**

This study presents the first comprehensive checklist of scleractinian coral species (Scleractinia Bourne, 1900) in Mangrove Bay, El Qoseir, Egypt. As a result of our 2023 field survey, we identified a total of 68 species across 29 genera and 14 families, spanning six reef habitats — three per study site — at two study locations (sheltered and current-exposed), with depths ranging from 0 to 9.5 m. We found that both scleractinian coral diversity and coral growth forms varied amongst the six habitats. Branching corals were found in each habitat, but were particularly abundant at the current-exposed reef edge, where they covered 67% of the habitat. Encrusting and massive corals became more prevalent with increasing depth at both study sites, with massive corals covering 72% at the sheltered deeper reef slope and encrusting corals covering 24% at the current-exposed deeper reef slope. Species of the genus *Porites* dominated the sheltered site at all depths and the deeper reef slope at the current-exposed site, while *Pocilloporaverrucosa* was most abundant at the exposed site’s reef edge and shallow slope.

We provide photographs confirming these new records and access to the raw data on the abundance, distribution and conservation status of these taxa. Forty-three percent of the scleractinian species are classified as "Least Concern," but six are identified as "Vulnerable" (9%). Reef cover analyses revealed algae as the dominant group in most habitats, while scleractinian coral cover ranged between approximately 17% at the current-exposed reef edge and almost 35% at the current-exposed shallow reef slope. *Millepora* contributed the most at the exposed reef edge with nearly 28% cover, but was scarce elsewhere; soft corals exhibited their highest abundance on the deeper slope.

Our observations serve as a critical baseline for future research and conservation efforts at Mangrove Bay by presenting an initial inventory of the local scleractinian communities and emphasise the importance of detailed species data in understanding and preserving coral reef ecosystems.

## Introduction

Coral reefs support the greatest biodiversity of marine organisms on Earth (Fisher et al. 2015, Anthony et al. 2020) and host more than 845 known reef-building scleractinian coral species worldwide (Veron et al. 2015, LaJeunesse et al. 2018). Despite their ecological and evolutionary significance, it is estimated that at least 50% of the world's coral reefs have already been decimated or severely damaged since the 1950s (Eddy et al. 2018, Eddy et al. 2021) due to global warming, pollution, overfishing and habitat destruction (Harborne et al. 2017). In particular, rising sea surface temperatures associated with global warming, have led to recurrent pan-tropical coral bleaching events in recent decades (Hughes et al. 2017) and remain the principal threat to coral reefs today. Under continued global warming, severe repercussions for coral reefs and the rich biodiversity they house are expected by the end of the century (Couce et al. 2013, Freeman et al. 2013, Jones et al. 2019). However, despite the current diminished state of many coral reefs worldwide, coral reef communities in the northern Red Sea have fared relatively well to pan-tropical bleaching events until recently, with the region often considered a potential refuge (Osman et al. 2018).

Due to their high diversity and ability to tolerate elevated temperatures, Red Sea scleractinians — particularly those in the northern Red Sea — have garnered significant interest in recent years (Hoegh-Guldberg et al. 2018, Fine et al. 2019, Banc-Prandi et al. 2022, Eladawy et al. 2022). Previous inventories have identified 346 scleractinian coral species throughout the Red Sea, with approximately 307 species in the north and central Red Sea and 200 species along the 1800 km fringing reefs of the Egyptian coastline (PERGSA 2010, DiBattista et al. 2016). Coral diversity in the northern Red Sea is supported by its unique thermal environment, where sea temperatures are cooler than in the central and southern Red Sea (Barreto et al. 2023). These lower temperatures, which are below the corals' physiological thermal maxima, may act as a buffer against thermal stress and bleaching events (Fine et al. 2013, Eladawy et al. 2022).

Given the potential significance of the northern Red Sea, detailed knowledge and data about scleractinian coral species’ distributions are essential for understanding coral biodiversity and potential future species loss within the region. Here, we present the first comprehensive checklist of coral species observed at Mangrove Bay (El Qoseir), Egypt, ensuring efficient and open access to this information in alignment with the FAIR (Findable, Accessible, Interoperable, Reusable) principles (Wilkinson et al. 2016). This publication supports the long-term monitoring of Red Sea coral reefs by providing an ecological baseline for Mangrove Bay, including coral diversity, coral growth form distributions and coral cover. Our contribution is timely given the recent reports of significant coral bleaching events in the region (Hanafy and Dosoky 2023). We anticipate that this checklist will benefit the research community by contributing to broader databases and analyses of the Red Sea, including community analyses and evaluations of scleractinian species’ distributions.

## Materials and methods

### Study area

**Figure 1.**
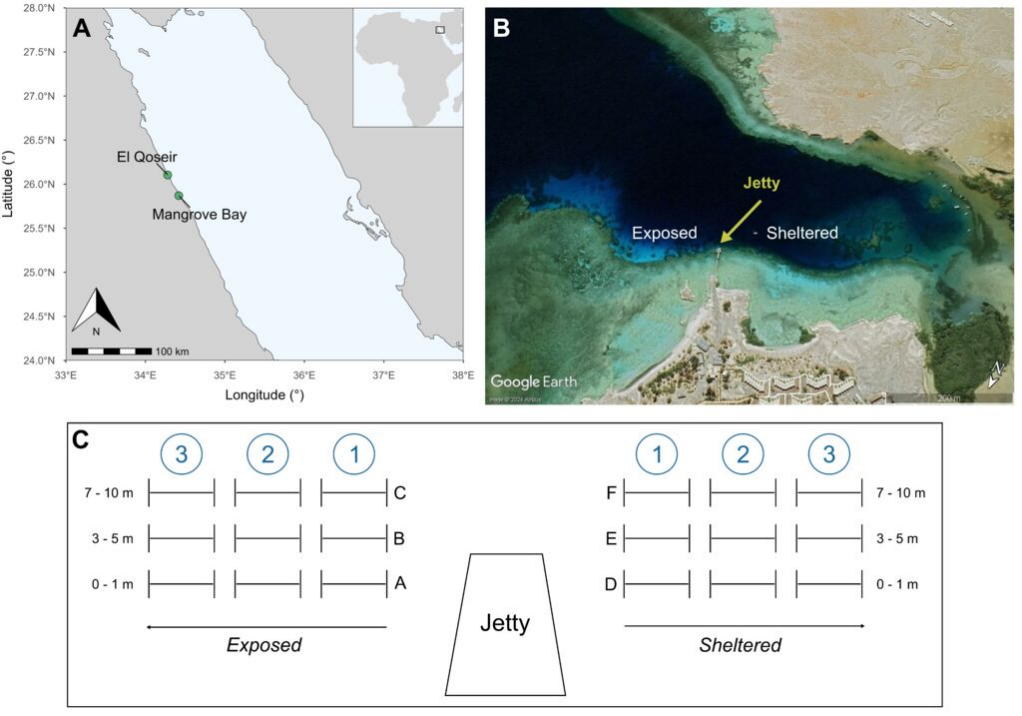
Map showing the location of Mangrove Bay and El Qoseir in Egypt (**A**). Google Earth image depicting the two study locations (**B**). Layout of the Line Intercept Transects (**C**).

Data collection took place between 19 and 25 October 2023, at Ducks Diving El Qoseir house reef, Mangrove Bay (Sharm Fugani, 25°52'12''N, 34°25'11''E), located approximately 29 km south of the coastal city of El Qoseir, Egypt (Fig. 1A). The two reef sites of Mangrove Bay, located 85 m apart, were selected as study locations due to their ease of accessibility and the good reef condition, which is largely due to the protective management by the local dive centre. The two sites are part of a typical modern Red Sea fringing reef system, characterised by its proximity to the shoreline, narrow reef flats and a comparatively steep reef slope (Loya 1972, Dullo and Montaggioni 1998, Mansour and Madkour 2015).

The house reef consists of a rocky intertidal zone and a sandy bottom, with an average depth of 15 m and a maximum depth of 30 m. As depth increased, the reef structure was progressively interrupted by stretches of sand. Sea surface temperatures in the northern Red Sea typically range from 21–28°C annually, with an average of 27°C in October (Nandkeolyar et al. 2013, Eladawy et al. 2017). The studied coral reef includes both a sheltered and a relatively more exposed site, the latter being predominantly current-exposed. A jetty served as a separation marker between them (Fig. 1B). Physical factors such as current exposure significantly impact the distribution of coral communities and other marine organisms (Riegl and Piller 1997, Bayraktarov et al. 2013). Therefore, we collected data from both sites of the reef (sheltered and current-exposed).

### Sampling protocol

To assess the coral community composition of the reef, the Line Intercept Transect (LIT) sampling protocol was employed (English et al. 1994). This method was elected over alternative sampling protocols (e.g. plotless methods) due to its standardised approach, recognised for its precision and replicability (English et al. 1994, Urbina-Barreto et al. 2021). Compared to plotless methods like time-based open search methods, LIT has the advantage of measuring the exact transition points of benthic organisms along the transect, thus providing a more accurate estimate of coral cover (Urbina-Barreto et al. 2021, Kuo et al. 2022). Although time-based methods might be more efficient for rapid assessments, they often compromise precision by relying on fewer data points and larger intervals which can overlook smaller or less abundant species (Kuo et al. 2022). The LIT method offers a standardised sampling protocol that minimises observer bias (e.g. identification expertise) and environmental bias (e.g. visibility conditions). As highlighted by Kuo et al. (2022), the LIT method provides a detailed assessment of benthic community structure, particularly when precise monitoring of coral cover and coral species diversity is the goal (Urbina-Barreto et al. 2021, Kuo et al. 2022). By using LIT, we captured multiple characteristics of the reef, such as biodiversity and coral cover, allowing future surveys to fully replicate our sampling approach to monitor these attributes. In this study, we applied the LIT method across different depths to capture variations in benthic communities at three depths.

**Figure 2.**
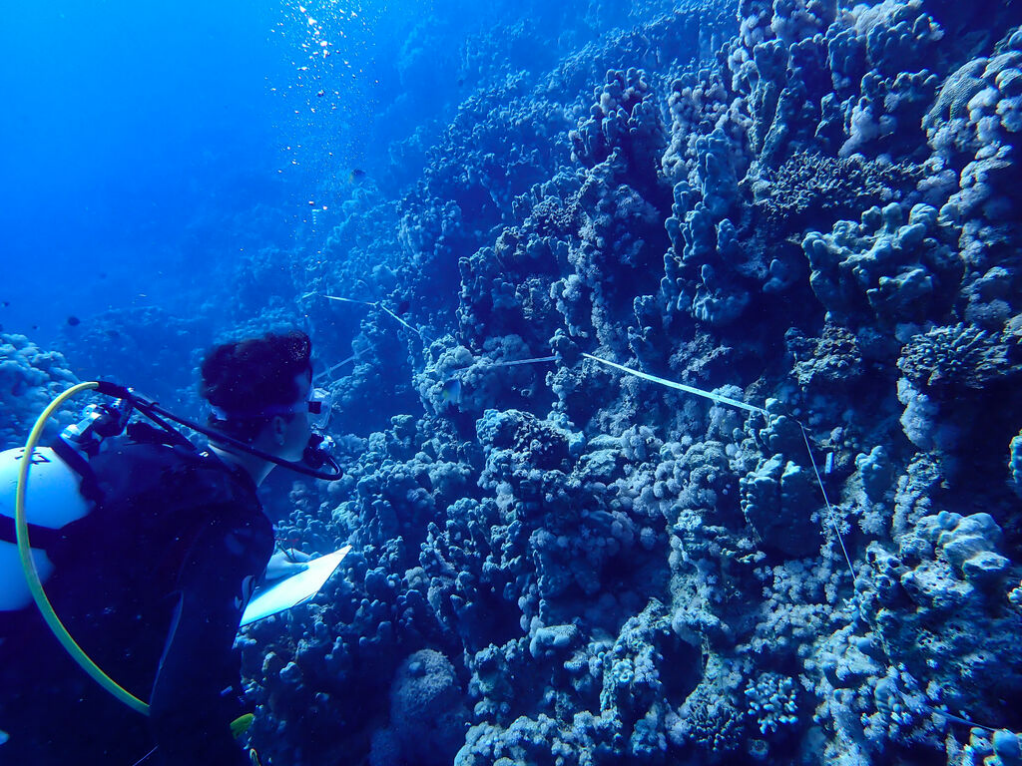
The transect line was stretched horizontally following the reef contour. Every biotic and abiotic component directly beneath the line was recorded. Photo credit: Victor Sebastian Scharnhorst.

At each study site, three replicate LITs, each with a minimum transect length of 15 m, were deployed at three depths: reef edge (0–1 m), shallow reef slope (3.7–4.75 m) and deeper reef slope (7.2–9.5 m). This methodology covered six habitats in total: sheltered reef edge, sheltered shallow reef slope, sheltered deeper reef slope, current-exposed reef edge, current-exposed shallow reef slope and current-exposed deeper reef slope. Transects were laid out using measurement tapes, with each transect replicate separated by a distance of at least 5 m (Fig. 1C). Each transect line was stretched tightly while following the horizontal reef contour, ensuring that it did not exceed a maximum distance of 15 cm from the substrate (English et al. 1994), (Fig. 2). A total of 18 transects were deployed, nine on each side of the jetty. Geographic coordinates were recorded for each site using the Garmin GPSMAP® 67 and using the World Geodetic System 1984 (WGS84).

### Data collection and analysis

Observational transect data were recorded via snorkelling (reef edge) and SCUBA (self-contained underwater breathing apparatus) diving surveys (reef slope). While following the transect line, all benthic organisms (e.g. corals, algae or molluscs) and substrate types (e.g. sand, rubble or rock) located directly beneath the measuring tape were recorded on a slate, including the length they covered along the transect line per occurrence (English et al. 1994; Table 1). A "gap" was noted when, 15 cm below the measuring tape, there was neither coral framework nor other substrate. As these added no data, they were excluded from further analysis. Scleractinian coral morphology and growth form were also documented. This was necessary because, although a single coral species can exhibit multiple growth forms, coral morphology tends to shift with increasing depth, independent of taxonomy (Kahng et al. 2019, Kramer et al. 2020).

As the LIT method has been shown to effectively capture a substantial diversity of coral species (Mohammed 2012) and given that photography has also been proven to yield high species counts (Alexandroff et al. 2016), we took three photographs of each intersected coral colony using an Olympus TG-6 underwater camera: an overview showing the growth form, a colony-focused image and a close-up of individual polyps and corallites.

All data from both reef sites at Mangrove Bay — 18 transects — were compiled into a single dataset for efficient data handling. This dataset includes information on transects, habitat details, depth measurements, exposure levels, coral growth forms and the type of abiogenic or biogenic benthic component directly beneath the measuring tape at the time of data collection. The data have been uploaded to a Zenodo public repository, accessible via https://doi.org/10.5281/zenodo.13926187. Using this dataset, we determined coral cover and the percentage of each coral growth form relative to the total coral cover, as well as the percentage of each abiogenic or biogenic benthic component. The transect length was defined as the total length of all categories, excluding gaps, as these provide no information and would compromise the accuracy of our assessment. This approach allows for a comprehensive analysis of coral reef biodiversity and provides a broader ecological context.

A key element of our species inventory is the checklist created using the Darwin Core (DwC) standard (Darwin Core Maintenance Group 2021). The DwC standard provides a structured framework for documenting species data, including taxonomy, distribution and occurrence. Applying DwC terms enhances data usability and facilitates reliable exchange and interpretation (Reyserhove et al. 2020, Darwin Core Maintenance Group 2021). A checklist adhering to this standard allows for easy tracking of changes in species presence, abundance and range. The dataset we compiled and used to create the checklist has also been uploaded to Zenodo (https://doi.org/10.5281/zenodo.13935417).

### Species identification

Species identification was performed on land by comparing the photographed morphology and growth forms of the scleractinians observed underwater with the *Corals of the World* database (http://www.coralsoftheworld.org/page/home/) (Veron et al. 2016) and relevant literature (Veron and Pichon 1976, Veron and Pichon 1979, Scheer and Pillai 1983, Hoeksema 1989, Sheppard and Sheppard 1991, Huang et al. 2014, Bouwmeester et al. 2015, Veron et al. 2015, Arrigoni et al. 2016a, Arrigoni et al. 2016b, Arrigoni et al. 2017, Arrigoni et al. 2019, Arrigoni et al. 2020). The World Register of Marine Species (WoRMS) (https://www.marinespecies.org/) was used for the latest taxonomic classification of the observed corals (Ahyong et al. 2024).

## Checklists

### Scleractinian species at Mangrove Bay, Egypt

#### Acroporidae

Verrill, 1901

67254691-648E-5D69-BDD9-6587841AF2BB

#### Acropora digitifera

(Dana, 1846)

095BF018-9766-5A23-8137-691698FAA872

##### Materials

- **Type status:** Other material. **Occurrence:** occurrenceRemarks: Observed and photographed on a current-exposed reef edge; occurrenceStatus: present; occurrenceID: 234084F0-C800-58F8-89A9-D7EC13813727; **Taxon:** scientificNameID: urn:lsid:marinespecies.org:taxname:207045; scientificName: *Acroporadigitifera*; kingdom: Animalia; phylum: Cnidaria; class: Anthozoa; taxonRank: Species; **Location:** continent: Africa; waterBody: Red Sea; country: Egypt; stateProvince: Muhafazah al Bahr al Ahmar; locality: Current-exposed coral reef located on the coastline of Egypt (Mangrove Bay), 29 km south of the coastal city of El Qoseir; verbatimDepth: 1; verbatimLatitude: N25°52.209′; verbatimLongitude: E034°25.181′; decimalLatitude: 25.87015; decimalLongitude: 34.4196833; geodeticDatum: WGS84; **Event:** samplingEffort: 3 x 20 m Line Intercept Transects; eventDate: 22-10-2023; year: 2023; habitat: current-exposed reef edge; **Record Level:** institutionCode: UNIVIE; collectionCode: Coral_Reef-Mangrove_Bay-Egypt-2023; basisOfRecord: HumanObservation
- **Type status:** Other material. **Occurrence:** occurrenceRemarks: Observed and photographed on a sheltered reef edge; occurrenceStatus: present; occurrenceID: 6CE31C97-1A22-54A0-96D2-51E023343597; **Taxon:** scientificNameID: urn:lsid:marinespecies.org:taxname:207045; scientificName: *Acroporadigitifera*; kingdom: Animalia; phylum: Cnidaria; class: Anthozoa; taxonRank: Species; **Location:** continent: Africa; waterBody: Red Sea; country: Egypt; stateProvince: Muhafazah al Bahr al Ahmar; locality: Sheltered coral reef located on the coastline of Egypt (Mangrove Bay), 29 km south of the coastal city of El Qoseir; verbatimDepth: 1; verbatimLatitude: N25°52.183′; verbatimLongitude: E034°25.140′; decimalLatitude: 25.8697167; decimalLongitude: 34.419; geodeticDatum: WGS84; **Event:** samplingEffort: 3 x 20 m Line Intercept Transects; eventDate: 19-10-2023; year: 2023; habitat: sheltered reef edge; **Record Level:** institutionCode: UNIVIE; collectionCode: Coral_Reef-Mangrove_Bay-Egypt-2023; basisOfRecord: HumanObservation

##### Conservation status

Near Threatened

##### Notes

Fig. 3 .

#### Acropora hemprichii

(Ehrenberg, 1834)

47EB5AB9-DA62-500E-8422-0C6B792EC68B

##### Materials

- **Type status:** Other material. **Occurrence:** occurrenceRemarks: Observed and photographed on a current-exposed shallow reef slope; occurrenceStatus: present; occurrenceID: 17B81438-B8C4-50F8-AB16-F506AF0F6FC5; **Taxon:** scientificNameID: urn:lsid:marinespecies.org:taxname:288207; scientificName: *Acroporahemprichii*; kingdom: Animalia; phylum: Cnidaria; class: Anthozoa; taxonRank: Species; **Location:** continent: Africa; waterBody: Red Sea; country: Egypt; stateProvince: Muhafazah al Bahr al Ahmar; locality: Current-exposed coral reef located on the coastline of Egypt (Mangrove Bay), 29 km south of the coastal city of El Qoseir; verbatimDepth: 4.3; verbatimLatitude: N25°52.209′; verbatimLongitude: E034°25.181′; decimalLatitude: 25.87015; decimalLongitude: 34.4196833; geodeticDatum: WGS84; **Event:** samplingEffort: 3 x 20 m Line Intercept Transects; eventDate: 24-10-2023; year: 2023; habitat: current-exposed shallow reef slope; **Record Level:** institutionCode: UNIVIE; collectionCode: Coral_Reef-Mangrove_Bay-Egypt-2023; basisOfRecord: HumanObservation
- **Type status:** Other material. **Occurrence:** occurrenceRemarks: Observed and photographed on a current-exposed deeper reef slope; occurrenceStatus: present; occurrenceID: 05A515FF-C43B-5B1A-A40C-609A536B62E0; **Taxon:** scientificNameID: urn:lsid:marinespecies.org:taxname:288207; scientificName: *Acroporahemprichii*; kingdom: Animalia; phylum: Cnidaria; class: Anthozoa; taxonRank: Species; **Location:** continent: Africa; waterBody: Red Sea; country: Egypt; stateProvince: Muhafazah al Bahr al Ahmar; locality: Current-exposed coral reef located on the coastline of Egypt (Mangrove Bay), 29 km south of the coastal city of El Qoseir; minimumDepthInMeters: 8.5; maximumDepthInMeters: 8.8; verbatimLatitude: N25°52.209′; verbatimLongitude: E034°25.181′; decimalLatitude: 25.87015; decimalLongitude: 34.4196833; geodeticDatum: WGS84; **Event:** samplingEffort: 3 x 20 m Line Intercept Transects; eventDate: 22-10-2023; year: 2023; habitat: current-exposed deeper reef slope; **Record Level:** institutionCode: UNIVIE; collectionCode: Coral_Reef-Mangrove_Bay-Egypt-2023; basisOfRecord: HumanObservation
- **Type status:** Other material. **Occurrence:** occurrenceRemarks: Observed and photographed on a sheltered shallow reef slope; occurrenceStatus: present; occurrenceID: AA07BB31-711F-5AB8-A8EE-FCE754A24F6B; **Taxon:** scientificNameID: urn:lsid:marinespecies.org:taxname:288207; scientificName: *Acroporahemprichii*; kingdom: Animalia; phylum: Cnidaria; class: Anthozoa; taxonRank: Species; **Location:** continent: Africa; waterBody: Red Sea; country: Egypt; stateProvince: Muhafazah al Bahr al Ahmar; locality: Sheltered coral reef located on the coastline of Egypt (Mangrove Bay), 29 km south of the coastal city of El Qoseir; minimumDepthInMeters: 3.7; maximumDepthInMeters: 4.75; verbatimLatitude: N25°52.183′; verbatimLongitude: E034°25.140′; decimalLatitude: 25.8697167; decimalLongitude: 34.419; geodeticDatum: WGS84; **Event:** samplingEffort: 3 x 20 m Line Intercept Transects; eventDate: 23-10-2023; year: 2023; habitat: sheltered shallow reef slope; **Record Level:** institutionCode: UNIVIE; collectionCode: Coral_Reef-Mangrove_Bay-Egypt-2023; basisOfRecord: HumanObservation

##### Conservation status

Vulnerable

##### Notes

Fig. 4 .

#### Acropora spp. f. spp.

Oken, 1815

34E99B0B-0841-5042-A5CE-3DC8ACADFD2C

##### Materials

- **Type status:** Other material. **Occurrence:** occurrenceRemarks: Observed and photographed on a current-exposed reef edge; occurrenceStatus: present; occurrenceID: 7CF6B52B-34FF-513D-AD10-7FB82D06BCCB; **Taxon:** scientificNameID: urn:lsid:marinespecies.org:taxname:205469; scientificName: *Acropora* spp.; kingdom: Animalia; phylum: Cnidaria; class: Anthozoa; taxonRank: Genus; **Location:** continent: Africa; waterBody: Red Sea; country: Egypt; stateProvince: Muhafazah al Bahr al Ahmar; locality: Current-exposed coral reef located on the coastline of Egypt (Mangrove Bay), 29 km south of the coastal city of El Qoseir; verbatimDepth: 1; verbatimLatitude: N25°52.209′; verbatimLongitude: E034°25.181′; decimalLatitude: 25.87015; decimalLongitude: 34.4196833; geodeticDatum: WGS84; **Event:** samplingEffort: 3 x 20 m Line Intercept Transects; eventDate: 23-10-2023; year: 2023; habitat: current-exposed reef edge; **Record Level:** institutionCode: UNIVIE; collectionCode: Coral_Reef-Mangrove_Bay-Egypt-2023; basisOfRecord: HumanObservation
- **Type status:** Other material. **Occurrence:** occurrenceRemarks: Observed and photographed on a current-exposed shallow reef slope; occurrenceStatus: present; occurrenceID: 76518BC5-7897-5407-96A4-08F4985B4DA8; **Taxon:** scientificNameID: urn:lsid:marinespecies.org:taxname:205469; scientificName: *Acropora* spp.; kingdom: Animalia; phylum: Cnidaria; class: Anthozoa; taxonRank: Genus; **Location:** continent: Africa; waterBody: Red Sea; country: Egypt; stateProvince: Muhafazah al Bahr al Ahmar; locality: Current-exposed coral reef located on the coastline of Egypt (Mangrove Bay), 29 km south of the coastal city of El Qoseir; verbatimDepth: 4.3; verbatimLatitude: N25°52.209′; verbatimLongitude: E034°25.181′; decimalLatitude: 25.87015; decimalLongitude: 34.4196833; geodeticDatum: WGS84; **Event:** samplingEffort: 3 x 20 m Line Intercept Transects; eventDate: 22-10-2023; year: 2023; habitat: current-exposed shallow reef slope; **Record Level:** institutionCode: UNIVIE; collectionCode: Coral_Reef-Mangrove_Bay-Egypt-2023; basisOfRecord: HumanObservation
- **Type status:** Other material. **Occurrence:** occurrenceRemarks: Observed and photographed on a current-exposed deeper reef slope; occurrenceStatus: present; occurrenceID: 0A9EFC80-6B64-5A41-BD2D-4E82F185878E; **Taxon:** scientificNameID: urn:lsid:marinespecies.org:taxname:205469; scientificName: *Acropora* spp.; kingdom: Animalia; phylum: Cnidaria; class: Anthozoa; taxonRank: Genus; **Location:** continent: Africa; waterBody: Red Sea; country: Egypt; stateProvince: Muhafazah al Bahr al Ahmar; locality: Current-exposed coral reef located on the coastline of Egypt (Mangrove Bay), 29 km south of the coastal city of El Qoseir; minimumDepthInMeters: 8.5; maximumDepthInMeters: 8.8; verbatimLatitude: N25°52.209′; verbatimLongitude: E034°25.181′; decimalLatitude: 25.87015; decimalLongitude: 34.4196833; geodeticDatum: WGS84; **Event:** samplingEffort: 3 x 20 m Line Intercept Transects; eventDate: 22-10-2023; year: 2023; habitat: current-exposed deeper reef slope; **Record Level:** institutionCode: UNIVIE; collectionCode: Coral_Reef-Mangrove_Bay-Egypt-2023; basisOfRecord: HumanObservation
- **Type status:** Other material. **Occurrence:** occurrenceRemarks: Observed and photographed on a sheltered reef edge; occurrenceStatus: present; occurrenceID: 4917B014-C337-5DE1-B279-449C89D8EAB6; **Taxon:** scientificNameID: urn:lsid:marinespecies.org:taxname:205469; scientificName: *Acropora* spp.; kingdom: Animalia; phylum: Cnidaria; class: Anthozoa; taxonRank: Genus; **Location:** continent: Africa; waterBody: Red Sea; country: Egypt; stateProvince: Muhafazah al Bahr al Ahmar; locality: Sheltered coral reef located on the coastline of Egypt (Mangrove Bay), 29 km south of the coastal city of El Qoseir; verbatimDepth: 1; verbatimLatitude: N25°52.183′; verbatimLongitude: E034°25.140′; decimalLatitude: 25.8697167; decimalLongitude: 34.419; geodeticDatum: WGS84; **Event:** samplingEffort: 3 x 20 m Line Intercept Transects; eventDate: 19-10-2023; year: 2023; habitat: sheltered reef edge; **Record Level:** institutionCode: UNIVIE; collectionCode: Coral_Reef-Mangrove_Bay-Egypt-2023; basisOfRecord: HumanObservation
- **Type status:** Other material. **Occurrence:** occurrenceRemarks: Observed and photographed on a sheltered shallow reef slope; occurrenceStatus: present; occurrenceID: 80BE18F6-BE98-5BB3-AE16-8EA4227DA552; **Taxon:** scientificNameID: urn:lsid:marinespecies.org:taxname:205469; scientificName: *Acropora* spp.; kingdom: Animalia; phylum: Cnidaria; class: Anthozoa; taxonRank: Genus; **Location:** continent: Africa; waterBody: Red Sea; country: Egypt; stateProvince: Muhafazah al Bahr al Ahmar; locality: Sheltered coral reef located on the coastline of Egypt (Mangrove Bay), 29 km south of the coastal city of El Qoseir; minimumDepthInMeters: 3.7; maximumDepthInMeters: 4.75; verbatimLatitude: N25°52.183′; verbatimLongitude: E034°25.140′; decimalLatitude: 25.8697167; decimalLongitude: 34.419; geodeticDatum: WGS84; **Event:** samplingEffort: 3 x 20 m Line Intercept Transects; eventDate: 23-10-2023; year: 2023; habitat: sheltered shallow reef slope; **Record Level:** institutionCode: UNIVIE; collectionCode: Coral_Reef-Mangrove_Bay-Egypt-2023; basisOfRecord: HumanObservation
- **Type status:** Other material. **Occurrence:** occurrenceRemarks: Observed and photographed on a sheltered deeper reef slope; occurrenceStatus: present; occurrenceID: 9DA6C8FD-D5BA-5973-BCF9-0DF994B9E8F9; **Taxon:** scientificNameID: urn:lsid:marinespecies.org:taxname:205469; scientificName: *Acropora* spp.; kingdom: Animalia; phylum: Cnidaria; class: Anthozoa; taxonRank: Genus; **Location:** continent: Africa; waterBody: Red Sea; country: Egypt; stateProvince: Muhafazah al Bahr al Ahmar; locality: Sheltered coral reef located on the coastline of Egypt (Mangrove Bay), 29 km south of the coastal city of El Qoseir; minimumDepthInMeters: 7.2; maximumDepthInMeters: 9.5; verbatimLatitude: N25°52.183′; verbatimLongitude: E034°25.140′; decimalLatitude: 25.8697167; decimalLongitude: 34.419; geodeticDatum: WGS84; **Event:** samplingEffort: 3 x 20 m Line Intercept Transects; eventDate: 23-10-2023; year: 2023; habitat: sheltered deeper reef slope; **Record Level:** institutionCode: UNIVIE; collectionCode: Coral_Reef-Mangrove_Bay-Egypt-2023; basisOfRecord: HumanObservation

##### Conservation status

Unknown

##### Notes

**Figure 5.**
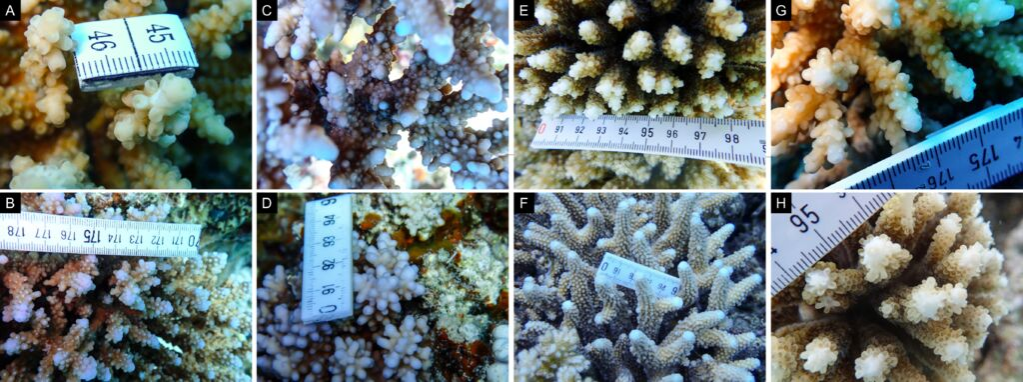
Amongst the *Acropora* colonies identified to genus level, eight morphotypes (**A**—**H**) were distinguished. Photo credits: (**A**) Lewis Alan Jones; (**B**), (**C**), (**F**) Joseph Wallace Daurella; (**D**) Theres Koch; (**E**), (**H**) Antonia Auer, Theda Schöchtner, Gözde Özer; (**G**) Victor Sebastian Scharnhorst.

Fig. 5; eight distinguished morphotypes.

#### Astreopora myriophthalma

(Lamarck, 1816)

E07C847D-0800-5A35-A008-EB2AB82CD59E

##### Materials

- **Type status:** Other material. **Occurrence:** occurrenceRemarks: Observed and photographed on a current-exposed shallow reef slope; occurrenceStatus: present; occurrenceID: BF2767C6-BCBB-5338-9BD7-361A7B7F636F; **Taxon:** scientificNameID: urn:lsid:marinespecies.org:taxname:207128; scientificName: *Astreoporamyriophthalma*; kingdom: Animalia; phylum: Cnidaria; class: Anthozoa; taxonRank: Species; **Location:** continent: Africa; waterBody: Red Sea; country: Egypt; stateProvince: Muhafazah al Bahr al Ahmar; locality: Current-exposed coral reef located on the coastline of Egypt (Mangrove Bay), 29 km south of the coastal city of El Qoseir; verbatimDepth: 4.3; verbatimLatitude: N25°52.209′; verbatimLongitude: E034°25.181′; decimalLatitude: 25.87015; decimalLongitude: 34.4196833; geodeticDatum: WGS84; **Event:** samplingEffort: 3 x 20 m Line Intercept Transects; eventDate: 24-10-2023; year: 2023; habitat: current-exposed shallow reef slope; **Record Level:** institutionCode: UNIVIE; collectionCode: Coral_Reef-Mangrove_Bay-Egypt-2023; basisOfRecord: HumanObservation
- **Type status:** Other material. **Occurrence:** occurrenceRemarks: Observed and photographed on a current-exposed deeper reef slope; occurrenceStatus: present; occurrenceID: 6EADA401-C476-580E-A08B-AEC2F4703F31; **Taxon:** scientificNameID: urn:lsid:marinespecies.org:taxname:207128; scientificName: *Astreoporamyriophthalma*; kingdom: Animalia; phylum: Cnidaria; class: Anthozoa; taxonRank: Species; **Location:** continent: Africa; waterBody: Red Sea; country: Egypt; stateProvince: Muhafazah al Bahr al Ahmar; locality: Current-exposed coral reef located on the coastline of Egypt (Mangrove Bay), 29 km south of the coastal city of El Qoseir; minimumDepthInMeters: 8.5; maximumDepthInMeters: 8.8; verbatimLatitude: N25°52.209′; verbatimLongitude: E034°25.181′; decimalLatitude: 25.87015; decimalLongitude: 34.4196833; geodeticDatum: WGS84; **Event:** samplingEffort: 3 x 20 m Line Intercept Transects; eventDate: 22-10-2023; year: 2023; habitat: current-exposed deeper reef slope; **Record Level:** institutionCode: UNIVIE; collectionCode: Coral_Reef-Mangrove_Bay-Egypt-2023; basisOfRecord: HumanObservation

##### Conservation status

Least Concern

##### Notes

Fig. 6 .

#### Montipora crypta

Turak, DeVantier & Veron, 2000

BEDD3290-FC56-5729-A3CB-EF65B786454F

##### Materials

- **Type status:** Other material. **Occurrence:** occurrenceRemarks: Observed and photographed on a current-exposed deeper reef slope; occurrenceStatus: present; occurrenceID: 4D1C95A2-2648-5F00-9239-6CDC0ECA84D2; **Taxon:** scientificNameID: urn:lsid:marinespecies.org:taxname:1617333; scientificName: *Montiporacrypta*; kingdom: Animalia; phylum: Cnidaria; class: Anthozoa; taxonRank: Species; **Location:** continent: Africa; waterBody: Red Sea; country: Egypt; stateProvince: Muhafazah al Bahr al Ahmar; locality: Current-exposed coral reef located on the coastline of Egypt (Mangrove Bay), 29 km south of the coastal city of El Qoseir; minimumDepthInMeters: 8.5; maximumDepthInMeters: 8.8; verbatimLatitude: N25°52.209′; verbatimLongitude: E034°25.181′; decimalLatitude: 25.87015; decimalLongitude: 34.4196833; geodeticDatum: WGS84; **Event:** samplingEffort: 3 x 20 m Line Intercept Transects; eventDate: 22-10-2023; year: 2023; habitat: current-exposed deeper reef slope; **Record Level:** institutionCode: UNIVIE; collectionCode: Coral_Reef-Mangrove_Bay-Egypt-2023; basisOfRecord: HumanObservation

##### Conservation status

Near Threatened

##### Notes

Fig. 7 .

#### Montipora danae

Milne Edwards & Haime, 1851

26816D73-C9FA-5585-A690-2BEC695DB6D8

##### Materials

- **Type status:** Other material. **Occurrence:** occurrenceRemarks: Observed and photographed on a current-exposed deeper reef slope; occurrenceStatus: present; occurrenceID: 431E7F81-E213-506F-9DB0-396E5ABDAD94; **Taxon:** scientificNameID: urn:lsid:marinespecies.org:taxname:207152; scientificName: *Montiporadanae*; kingdom: Animalia; phylum: Cnidaria; class: Anthozoa; taxonRank: Species; **Location:** continent: Africa; waterBody: Red Sea; country: Egypt; stateProvince: Muhafazah al Bahr al Ahmar; locality: Current-exposed coral reef located on the coastline of Egypt (Mangrove Bay), 29 km south of the coastal city of El Qoseir; minimumDepthInMeters: 8.5; maximumDepthInMeters: 8.8; verbatimLatitude: N25°52.209′; verbatimLongitude: E034°25.181′; decimalLatitude: 25.87015; decimalLongitude: 34.4196833; geodeticDatum: WGS84; **Event:** samplingEffort: 3 x 20 m Line Intercept Transects; eventDate: 22-10-2023; year: 2023; habitat: current-exposed deeper reef slope; **Record Level:** institutionCode: UNIVIE; collectionCode: Coral_Reef-Mangrove_Bay-Egypt-2023; basisOfRecord: HumanObservation

##### Conservation status

Least Concern

##### Notes

Fig. 8 .

#### Montipora efflorescens

Bernard, 1897

32F40F7C-249A-56C5-85C2-C0404A277507

##### Materials

- **Type status:** Other material. **Occurrence:** occurrenceRemarks: Observed and photographed on a current-exposed shallow reef slope; occurrenceStatus: present; occurrenceID: CD7502CE-2893-5276-9547-A4553BA1DEC7; **Taxon:** scientificNameID: urn:lsid:marinespecies.org:taxname:207163; scientificName: *Montiporaefflorescens*; kingdom: Animalia; phylum: Cnidaria; class: Anthozoa; taxonRank: Species; **Location:** continent: Africa; waterBody: Red Sea; country: Egypt; stateProvince: Muhafazah al Bahr al Ahmar; locality: Current-exposed coral reef located on the coastline of Egypt (Mangrove Bay), 29 km south of the coastal city of El Qoseir; verbatimDepth: 4.3; verbatimLatitude: N25°52.209′; verbatimLongitude: E034°25.181′; decimalLatitude: 25.87015; decimalLongitude: 34.4196833; geodeticDatum: WGS84; **Event:** samplingEffort: 3 x 20 m Line Intercept Transects; eventDate: 24-10-2023; year: 2023; habitat: current-exposed shallow reef slope; **Record Level:** institutionCode: UNIVIE; collectionCode: Coral_Reef-Mangrove_Bay-Egypt-2023; basisOfRecord: HumanObservation
- **Type status:** Other material. **Occurrence:** occurrenceRemarks: Observed and photographed on a current-exposed deeper reef slope; occurrenceStatus: present; occurrenceID: EB706A08-1FAF-55DE-9736-D1149C784DF8; **Taxon:** scientificNameID: urn:lsid:marinespecies.org:taxname:207163; scientificName: *Montiporaefflorescens*; kingdom: Animalia; phylum: Cnidaria; class: Anthozoa; taxonRank: Species; **Location:** continent: Africa; waterBody: Red Sea; country: Egypt; stateProvince: Muhafazah al Bahr al Ahmar; locality: Current-exposed coral reef located on the coastline of Egypt (Mangrove Bay), 29 km south of the coastal city of El Qoseir; minimumDepthInMeters: 8.5; maximumDepthInMeters: 8.8; verbatimLatitude: N25°52.209′; verbatimLongitude: E034°25.181′; decimalLatitude: 25.87015; decimalLongitude: 34.4196833; geodeticDatum: WGS84; **Event:** samplingEffort: 3 x 20 m Line Intercept Transects; eventDate: 24-10-2023; year: 2023; habitat: current-exposed deeper reef slope; **Record Level:** institutionCode: UNIVIE; collectionCode: Coral_Reef-Mangrove_Bay-Egypt-2023; basisOfRecord: HumanObservation

##### Conservation status

Near Threatened

##### Notes

Fig. 9 .

#### Montipora cf. grisea

Bernard, 1897

8117CF0E-F0F9-5233-A104-1F1F205CA301

##### Materials

- **Type status:** Other material. **Occurrence:** occurrenceRemarks: Observed and photographed on a current-exposed shallow reef slope; occurrenceStatus: present; occurrenceID: ED26F58A-830E-5BE0-9E11-10541184C1F8; **Taxon:** scientificNameID: urn:lsid:marinespecies.org:taxname:287709; scientificName: Montiporacf.grisea; kingdom: Animalia; phylum: Cnidaria; class: Anthozoa; taxonRank: Species; **Location:** continent: Africa; waterBody: Red Sea; country: Egypt; stateProvince: Muhafazah al Bahr al Ahmar; locality: Current-exposed coral reef located on the coastline of Egypt (Mangrove Bay), 29 km south of the coastal city of El Qoseir; verbatimDepth: 4.3; verbatimLatitude: N25°52.209′; verbatimLongitude: E034°25.181′; decimalLatitude: 25.87015; decimalLongitude: 34.4196833; geodeticDatum: WGS84; **Event:** samplingEffort: 3 x 20 m Line Intercept Transects; eventDate: 24-10-2023; year: 2023; habitat: current-exposed shallow reef slope; **Record Level:** institutionCode: UNIVIE; collectionCode: Coral_Reef-Mangrove_Bay-Egypt-2023; basisOfRecord: HumanObservation

##### Conservation status

Least Concern

##### Notes

Fig. 10 .

#### Montipora maeandrina

(Ehrenberg, 1834)

8E8EED16-D36A-5866-B292-3811502E3B1C

##### Materials

- **Type status:** Other material. **Occurrence:** occurrenceRemarks: Observed and photographed on a current-exposed shallow reef slope; occurrenceStatus: present; occurrenceID: 0B3BF3B2-8A74-5617-A260-7EEDB26B5375; **Taxon:** scientificNameID: urn:lsid:marinespecies.org:taxname:207188; scientificName: *Montiporamaeandrina*; kingdom: Animalia; phylum: Cnidaria; class: Anthozoa; taxonRank: Species; **Location:** continent: Africa; waterBody: Red Sea; country: Egypt; stateProvince: Muhafazah al Bahr al Ahmar; locality: Current-exposed coral reef located on the coastline of Egypt (Mangrove Bay), 29 km south of the coastal city of El Qoseir; verbatimDepth: 4.3; verbatimLatitude: N25°52.209′; verbatimLongitude: E034°25.181′; decimalLatitude: 25.87015; decimalLongitude: 34.4196833; geodeticDatum: WGS84; **Event:** samplingEffort: 3 x 20 m Line Intercept Transects; eventDate: 24-10-2023; year: 2023; habitat: current-exposed shallow reef slope; **Record Level:** institutionCode: UNIVIE; collectionCode: Coral_Reef-Mangrove_Bay-Egypt-2023; basisOfRecord: HumanObservation
- **Type status:** Other material. **Occurrence:** occurrenceRemarks: Observed and photographed on a current-exposed deeper reef slope; occurrenceStatus: present; occurrenceID: 05B82702-12D8-5F08-9651-61F3FF2F78EB; **Taxon:** scientificNameID: urn:lsid:marinespecies.org:taxname:207188; scientificName: *Montiporamaeandrina*; kingdom: Animalia; phylum: Cnidaria; class: Anthozoa; taxonRank: Species; **Location:** continent: Africa; waterBody: Red Sea; country: Egypt; stateProvince: Muhafazah al Bahr al Ahmar; locality: Current-exposed coral reef located on the coastline of Egypt (Mangrove Bay), 29 km south of the coastal city of El Qoseir; minimumDepthInMeters: 8.5; maximumDepthInMeters: 8.8; verbatimLatitude: N25°52.209′; verbatimLongitude: E034°25.181′; decimalLatitude: 25.87015; decimalLongitude: 34.4196833; geodeticDatum: WGS84; **Event:** samplingEffort: 3 x 20 m Line Intercept Transects; eventDate: 22-10-2023; year: 2023; habitat: current-exposed deeper reef slope; **Record Level:** institutionCode: UNIVIE; collectionCode: Coral_Reef-Mangrove_Bay-Egypt-2023; basisOfRecord: HumanObservation
- **Type status:** Other material. **Occurrence:** occurrenceRemarks: Observed and photographed on a sheltered shallow reef slope; occurrenceStatus: present; occurrenceID: E85E9A47-AA94-5720-BCC6-41539FAEBD8B; **Taxon:** scientificNameID: urn:lsid:marinespecies.org:taxname:207188; scientificName: *Montiporamaeandrina*; kingdom: Animalia; phylum: Cnidaria; class: Anthozoa; taxonRank: Species; **Location:** continent: Africa; waterBody: Red Sea; country: Egypt; stateProvince: Muhafazah al Bahr al Ahmar; locality: Sheltered coral reef located on the coastline of Egypt (Mangrove Bay), 29 km south of the coastal city of El Qoseir; minimumDepthInMeters: 3.7; maximumDepthInMeters: 4.75; verbatimLatitude: N25°52.183′; verbatimLongitude: E034°25.140′; decimalLatitude: 25.8697167; decimalLongitude: 34.419; geodeticDatum: WGS84; **Event:** samplingEffort: 3 x 20 m Line Intercept Transects; eventDate: 25-10-2023; year: 2023; habitat: sheltered shallow reef slope; **Record Level:** institutionCode: UNIVIE; collectionCode: Coral_Reef-Mangrove_Bay-Egypt-2023; basisOfRecord: HumanObservation
- **Type status:** Other material. **Occurrence:** occurrenceRemarks: Observed and photographed on a sheltered deeper reef slope; occurrenceStatus: present; occurrenceID: C2192C7C-5B74-5012-8F1E-C85538A79F4F; **Taxon:** scientificNameID: urn:lsid:marinespecies.org:taxname:207188; scientificName: *Montiporamaeandrina*; kingdom: Animalia; phylum: Cnidaria; class: Anthozoa; taxonRank: Species; **Location:** continent: Africa; waterBody: Red Sea; country: Egypt; stateProvince: Muhafazah al Bahr al Ahmar; locality: Sheltered coral reef located on the coastline of Egypt (Mangrove Bay), 29 km south of the coastal city of El Qoseir; minimumDepthInMeters: 7.2; maximumDepthInMeters: 9.5; verbatimLatitude: N25°52.183′; verbatimLongitude: E034°25.140′; decimalLatitude: 25.8697167; decimalLongitude: 34.419; geodeticDatum: WGS84; **Event:** samplingEffort: 3 x 20 m Line Intercept Transects; eventDate: 23-10-2023; year: 2023; habitat: sheltered deeper reef slope; **Record Level:** institutionCode: UNIVIE; collectionCode: Coral_Reef-Mangrove_Bay-Egypt-2023; basisOfRecord: HumanObservation

##### Conservation status

Vulnerable

##### Notes

Fig. 11 .

#### Montipora spp.

Blainville, 1830

8F912817-7103-51AD-930C-5740F7C9A8F4

##### Materials

- **Type status:** Other material. **Occurrence:** occurrenceRemarks: Observed and photographed on a current-exposed shallow reef slope; occurrenceStatus: present; occurrenceID: 550F703B-2189-540F-B6DF-9B0715420BAD; **Taxon:** scientificNameID: urn:lsid:marinespecies.org:taxname:203834; scientificName: *Montipora* spp.; kingdom: Animalia; phylum: Cnidaria; class: Anthozoa; taxonRank: Genus; **Location:** continent: Africa; waterBody: Red Sea; country: Egypt; stateProvince: Muhafazah al Bahr al Ahmar; locality: Current-exposed coral reef located on the coastline of Egypt (Mangrove Bay), 29 km south of the coastal city of El Qoseir; verbatimDepth: 4.3; verbatimLatitude: N25°52.209′; verbatimLongitude: E034°25.181′; decimalLatitude: 25.87015; decimalLongitude: 34.4196833; geodeticDatum: WGS84; **Event:** samplingEffort: 3 x 20 m Line Intercept Transects; eventDate: 22-10-2023; year: 2023; habitat: current-exposed shallow reef slope; **Record Level:** institutionCode: UNIVIE; collectionCode: Coral_Reef-Mangrove_Bay-Egypt-2023; basisOfRecord: HumanObservation
- **Type status:** Other material. **Occurrence:** occurrenceRemarks: Observed and photographed on a sheltered shallow reef slope; occurrenceStatus: present; occurrenceID: 64835677-69D7-5737-A4FF-4C52314F5F22; **Taxon:** scientificNameID: urn:lsid:marinespecies.org:taxname:203834; scientificName: *Montipora* sp.; kingdom: Animalia; phylum: Cnidaria; class: Anthozoa; taxonRank: Genus; **Location:** continent: Africa; waterBody: Red Sea; country: Egypt; stateProvince: Muhafazah al Bahr al Ahmar; locality: Sheltered coral reef located on the coastline of Egypt (Mangrove Bay), 29 km south of the coastal city of El Qoseir; minimumDepthInMeters: 3.7; maximumDepthInMeters: 4.75; verbatimLatitude: N25°52.183′; verbatimLongitude: E034°25.140′; decimalLatitude: 25.8697167; decimalLongitude: 34.419; geodeticDatum: WGS84; **Event:** samplingEffort: 3 x 20 m Line Intercept Transects; eventDate: 25-10-2023; year: 2023; habitat: sheltered shallow reef slope; **Record Level:** institutionCode: UNIVIE; collectionCode: Coral_Reef-Mangrove_Bay-Egypt-2023; basisOfRecord: HumanObservation

##### Conservation status

Unknown

##### Notes

**Figure 12.**
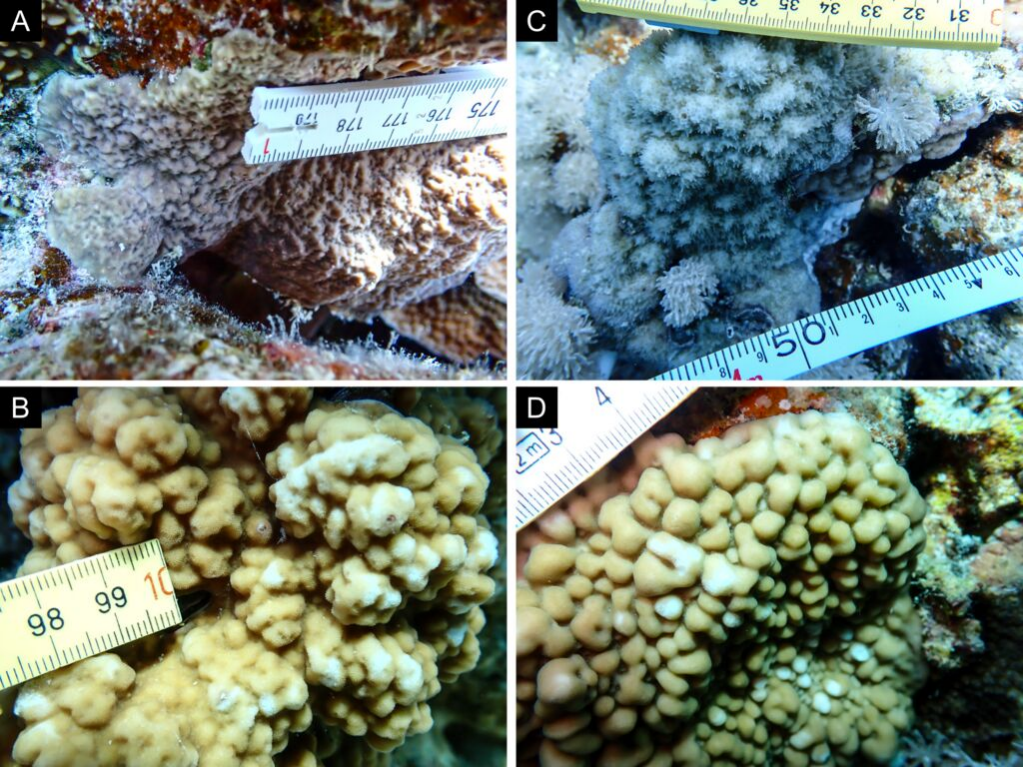
Amongst the *Montipora* colonies identified to genus level, four morphotypes (**A**—**D**) were distinguished. Photo credits: (**A**), (**C**) Joseph Wallace Daurella; (**B**), (**D**) Victor Sebastian Scharnhorst.

Fig. 12; four distinguished morphotypes.

#### Montipora tuberculosa

(Lamarck, 1816)

FC9ECE4A-86C5-5629-85A5-1D37CE2B09A2

##### Materials

- **Type status:** Other material. **Occurrence:** occurrenceRemarks: Observed and photographed on a current-exposed reef edge; occurrenceStatus: present; occurrenceID: 941F7AA6-6F1B-5AFD-8820-1E056DADFF7C; **Taxon:** scientificNameID: urn:lsid:marinespecies.org:taxname:207156; scientificName: *Montiporatuberculosa*; kingdom: Animalia; phylum: Cnidaria; class: Anthozoa; taxonRank: Species; **Location:** continent: Africa; waterBody: Red Sea; country: Egypt; stateProvince: Muhafazah al Bahr al Ahmar; locality: Current-exposed coral reef located on the coastline of Egypt (Mangrove Bay), 29 km south of the coastal city of El Qoseir; verbatimDepth: 1; verbatimLatitude: N25°52.209′; verbatimLongitude: E034°25.181′; decimalLatitude: 25.87015; decimalLongitude: 34.4196833; geodeticDatum: WGS84; **Event:** samplingEffort: 3 x 20 m Line Intercept Transects; eventDate: 23-10-2023; year: 2023; habitat: current-exposed reef edge; **Record Level:** institutionCode: UNIVIE; collectionCode: Coral_Reef-Mangrove_Bay-Egypt-2023; basisOfRecord: HumanObservation
- **Type status:** Other material. **Occurrence:** occurrenceRemarks: Observed and photographed on a current-exposed shallow reef slope; occurrenceStatus: present; occurrenceID: 376F5D6A-149F-56E8-9A83-57B615D89988; **Taxon:** scientificNameID: urn:lsid:marinespecies.org:taxname:207156; scientificName: *Montiporatuberculosa*; kingdom: Animalia; phylum: Cnidaria; class: Anthozoa; taxonRank: Species; **Location:** continent: Africa; waterBody: Red Sea; country: Egypt; stateProvince: Muhafazah al Bahr al Ahmar; locality: Current-exposed coral reef located on the coastline of Egypt (Mangrove Bay), 29 km south of the coastal city of El Qoseir; verbatimDepth: 4.3; verbatimLatitude: N25°52.209′; verbatimLongitude: E034°25.181′; decimalLatitude: 25.87015; decimalLongitude: 34.4196833; geodeticDatum: WGS84; **Event:** samplingEffort: 3 x 20 m Line Intercept Transects; eventDate: 22-10-2023; year: 2023; habitat: current-exposed shallow reef slope; **Record Level:** institutionCode: UNIVIE; collectionCode: Coral_Reef-Mangrove_Bay-Egypt-2023; basisOfRecord: HumanObservation
- **Type status:** Other material. **Occurrence:** occurrenceRemarks: Observed and photographed on a sheltered reef edge; occurrenceStatus: present; occurrenceID: C452B179-F940-58C0-B6CC-23C79D922C0A; **Taxon:** scientificNameID: urn:lsid:marinespecies.org:taxname:207156; scientificName: *Montiporatuberculosa*; kingdom: Animalia; phylum: Cnidaria; class: Anthozoa; taxonRank: Species; **Location:** continent: Africa; waterBody: Red Sea; country: Egypt; stateProvince: Muhafazah al Bahr al Ahmar; locality: Sheltered coral reef located on the coastline of Egypt (Mangrove Bay), 29 km south of the coastal city of El Qoseir; verbatimDepth: 1; verbatimLatitude: N25°52.183′; verbatimLongitude: E034°25.140′; decimalLatitude: 25.8697167; decimalLongitude: 34.419; geodeticDatum: WGS84; **Event:** samplingEffort: 3 x 20 m Line Intercept Transects; eventDate: 19-10-2023; year: 2023; habitat: sheltered reef edge; **Record Level:** institutionCode: UNIVIE; collectionCode: Coral_Reef-Mangrove_Bay-Egypt-2023; basisOfRecord: HumanObservation
- **Type status:** Other material. **Occurrence:** occurrenceRemarks: Observed and photographed on a sheltered deeper reef slope; occurrenceStatus: present; occurrenceID: B2DE15C6-9363-5161-BEDE-616F2DDB350F; **Taxon:** scientificNameID: urn:lsid:marinespecies.org:taxname:207156; scientificName: *Montiporatuberculosa*; kingdom: Animalia; phylum: Cnidaria; class: Anthozoa; taxonRank: Species; **Location:** continent: Africa; waterBody: Red Sea; country: Egypt; stateProvince: Muhafazah al Bahr al Ahmar; locality: Sheltered coral reef located on the coastline of Egypt (Mangrove Bay), 29 km south of the coastal city of El Qoseir; minimumDepthInMeters: 7.2; maximumDepthInMeters: 9.5; verbatimLatitude: N25°52.183′; verbatimLongitude: E034°25.140′; decimalLatitude: 25.8697167; decimalLongitude: 34.419; geodeticDatum: WGS84; **Event:** samplingEffort: 3 x 20 m Line Intercept Transects; eventDate: 23-10-2023; year: 2023; habitat: sheltered deeper reef slope; **Record Level:** institutionCode: UNIVIE; collectionCode: Coral_Reef-Mangrove_Bay-Egypt-2023; basisOfRecord: HumanObservation

##### Conservation status

Least Concern

##### Notes

Fig. 13 .

#### Agariciidae

Gray, 1847

3CD9687E-B6FC-5001-A148-9331B69D8BCB

#### Gardineroseris planulata

(Dana, 1846)

6BC6BA6B-19D0-50EA-A0F5-F0BA335C408F

##### Materials

- **Type status:** Other material. **Occurrence:** occurrenceRemarks: Observed and photographed on a sheltered shallow reef slope; occurrenceStatus: present; occurrenceID: BB4537AB-8A2C-5D65-A4AD-5CAF558B6E87; **Taxon:** scientificNameID: urn:lsid:marinespecies.org:taxname:207274; scientificName: *Gardineroserisplanulata*; kingdom: Animalia; phylum: Cnidaria; class: Anthozoa; taxonRank: Species; **Location:** continent: Africa; waterBody: Red Sea; country: Egypt; stateProvince: Muhafazah al Bahr al Ahmar; locality: Sheltered coral reef located on the coastline of Egypt (Mangrove Bay), 29 km south of the coastal city of El Qoseir; minimumDepthInMeters: 3.7; maximumDepthInMeters: 4.75; verbatimLatitude: N25°52.183′; verbatimLongitude: E034°25.140′; decimalLatitude: 25.8697167; decimalLongitude: 34.419; geodeticDatum: WGS84; **Event:** samplingEffort: 3 x 20 m Line Intercept Transects; eventDate: 25-10-2023; year: 2023; habitat: sheltered shallow reef slope; **Record Level:** institutionCode: UNIVIE; collectionCode: Coral_Reef-Mangrove_Bay-Egypt-2023; basisOfRecord: HumanObservation

##### Conservation status

Least Concern

##### Notes

Fig. 14.

#### Leptoseris mycetoseroides

Wells, 1954

6C5DA7FF-0018-57B1-9E53-D149BD5DF90E

##### Materials

- **Type status:** Other material. **Occurrence:** occurrenceRemarks: Observed and photographed on a sheltered shallow reef slope; occurrenceStatus: present; occurrenceID: 13E6AAE5-CEE1-5C85-B6AA-A1D1EE676C16; **Taxon:** scientificNameID: urn:lsid:marinespecies.org:taxname:207283; scientificName: *Leptoserismycetoseroides*; kingdom: Animalia; phylum: Cnidaria; class: Anthozoa; taxonRank: Species; **Location:** continent: Africa; waterBody: Red Sea; country: Egypt; stateProvince: Muhafazah al Bahr al Ahmar; locality: Sheltered coral reef located on the coastline of Egypt (Mangrove Bay), 29 km south of the coastal city of El Qoseir; minimumDepthInMeters: 3.7; maximumDepthInMeters: 4.75; verbatimLatitude: N25°52.183′; verbatimLongitude: E034°25.140′; decimalLatitude: 25.8697167; decimalLongitude: 34.419; geodeticDatum: WGS84; **Event:** samplingEffort: 3 x 20 m Line Intercept Transects; eventDate: 25-10-2023; year: 2023; habitat: sheltered shallow reef slope; **Record Level:** institutionCode: UNIVIE; collectionCode: Coral_Reef-Mangrove_Bay-Egypt-2023; basisOfRecord: HumanObservation

##### Conservation status

Least Concern

##### Notes

Fig. 15 .

#### Pavona diffluens

(Lamarck, 1816)

EB8C368E-5AE2-5FE6-96F8-518961B51D22

##### Materials

- **Type status:** Other material. **Occurrence:** occurrenceRemarks: Observed and photographed on a current-exposed shallow reef slope; occurrenceStatus: present; occurrenceID: 6A4753E9-1B14-5212-8182-EEE90CE4A86B; **Taxon:** scientificNameID: urn:lsid:marinespecies.org:taxname:207295; scientificName: *Pavonadiffluens*; kingdom: Animalia; phylum: Cnidaria; class: Anthozoa; taxonRank: Species; **Location:** continent: Africa; waterBody: Red Sea; country: Egypt; stateProvince: Muhafazah al Bahr al Ahmar; locality: Current-exposed coral reef located on the coastline of Egypt (Mangrove Bay), 29 km south of the coastal city of El Qoseir; verbatimDepth: 4.3; verbatimLatitude: N25°52.209′; verbatimLongitude: E034°25.181′; decimalLatitude: 25.87015; decimalLongitude: 34.4196833; geodeticDatum: WGS84; **Event:** samplingEffort: 3 x 20 m Line Intercept Transects; eventDate: 22-10-2023; year: 2023; habitat: current-exposed shallow reef slope; **Record Level:** institutionCode: UNIVIE; collectionCode: Coral_Reef-Mangrove_Bay-Egypt-2023; basisOfRecord: HumanObservation
- **Type status:** Other material. **Occurrence:** occurrenceRemarks: Observed and photographed on a sheltered deeper reef slope; occurrenceStatus: present; occurrenceID: EEEB2E87-1BE2-55C8-AA09-A9D4A379F04F; **Taxon:** scientificNameID: urn:lsid:marinespecies.org:taxname:207295; scientificName: *Pavonadiffluens*; kingdom: Animalia; phylum: Cnidaria; class: Anthozoa; taxonRank: Species; **Location:** continent: Africa; waterBody: Red Sea; country: Egypt; stateProvince: Muhafazah al Bahr al Ahmar; locality: Sheltered coral reef located on the coastline of Egypt (Mangrove Bay), 29 km south of the coastal city of El Qoseir; minimumDepthInMeters: 7.2; maximumDepthInMeters: 9.5; verbatimLatitude: N25°52.183′; verbatimLongitude: E034°25.140′; decimalLatitude: 25.8697167; decimalLongitude: 34.419; geodeticDatum: WGS84; **Event:** samplingEffort: 3 x 20 m Line Intercept Transects; eventDate: 25-10-2023; year: 2023; habitat: sheltered deeper reef slope; **Record Level:** institutionCode: UNIVIE; collectionCode: Coral_Reef-Mangrove_Bay-Egypt-2023; basisOfRecord: HumanObservation

##### Conservation status

Vulnerable

##### Notes

Fig. 16 .

#### Pavona spp.

Lamarck, 1801

1B953B69-618B-5AAF-8667-F7A230694BC6

##### Materials

- **Type status:** Other material. **Occurrence:** occurrenceRemarks: Observed and photographed on a current-exposed shallow reef slope; occurrenceStatus: present; occurrenceID: 232BB3E2-252F-5B2F-8146-FE31FA671C90; **Taxon:** scientificNameID: urn:lsid:marinespecies.org:taxname:206614; scientificName: *Pavona* sp.; kingdom: Animalia; phylum: Cnidaria; class: Anthozoa; taxonRank: Genus; **Location:** continent: Africa; waterBody: Red Sea; country: Egypt; stateProvince: Muhafazah al Bahr al Ahmar; locality: Current-exposed coral reef located on the coastline of Egypt (Mangrove Bay), 29 km south of the coastal city of El Qoseir; verbatimDepth: 4.3; verbatimLatitude: N25°52.209′; verbatimLongitude: E034°25.181′; decimalLatitude: 25.87015; decimalLongitude: 34.4196833; geodeticDatum: WGS84; **Event:** samplingEffort: 3 x 20 m Line Intercept Transects; eventDate: 22-10-2023; year: 2023; habitat: current-exposed shallow reef slope; **Record Level:** institutionCode: UNIVIE; collectionCode: Coral_Reef-Mangrove_Bay-Egypt-2023; basisOfRecord: HumanObservation
- **Type status:** Other material. **Occurrence:** occurrenceRemarks: Observed and photographed on a sheltered reef edge; occurrenceStatus: present; occurrenceID: 2E5B04A6-BF2F-583D-B1C5-A81188DE392E; **Taxon:** scientificNameID: urn:lsid:marinespecies.org:taxname:206614; scientificName: *Pavona* sp.; kingdom: Animalia; phylum: Cnidaria; class: Anthozoa; taxonRank: Genus; **Location:** continent: Africa; waterBody: Red Sea; country: Egypt; stateProvince: Muhafazah al Bahr al Ahmar; locality: Sheltered coral reef located on the coastline of Egypt (Mangrove Bay), 29 km south of the coastal city of El Qoseir; verbatimDepth: 1; verbatimLatitude: N25°52.183′; verbatimLongitude: E034°25.140′; decimalLatitude: 25.8697167; decimalLongitude: 34.419; geodeticDatum: WGS84; **Event:** samplingEffort: 3 x 20 m Line Intercept Transects; eventDate: 20-10-2023; year: 2023; habitat: sheltered reef edge; **Record Level:** institutionCode: UNIVIE; collectionCode: Coral_Reef-Mangrove_Bay-Egypt-2023; basisOfRecord: HumanObservation
- **Type status:** Other material. **Occurrence:** occurrenceRemarks: Observed and photographed on a sheltered shallow reef slope; occurrenceStatus: present; occurrenceID: F21EDB82-6C90-5EBE-8C28-C16CD6D539D7; **Taxon:** scientificNameID: urn:lsid:marinespecies.org:taxname:206614; scientificName: *Pavona* sp.; kingdom: Animalia; phylum: Cnidaria; class: Anthozoa; taxonRank: Genus; **Location:** continent: Africa; waterBody: Red Sea; country: Egypt; stateProvince: Muhafazah al Bahr al Ahmar; locality: Sheltered coral reef located on the coastline of Egypt (Mangrove Bay), 29 km south of the coastal city of El Qoseir; minimumDepthInMeters: 3.7; maximumDepthInMeters: 4.75; verbatimLatitude: N25°52.183′; verbatimLongitude: E034°25.140′; decimalLatitude: 25.8697167; decimalLongitude: 34.419; geodeticDatum: WGS84; **Event:** samplingEffort: 3 x 20 m Line Intercept Transects; eventDate: 25-10-2023; year: 2023; habitat: sheltered shallow reef slope; **Record Level:** institutionCode: UNIVIE; collectionCode: Coral_Reef-Mangrove_Bay-Egypt-2023; basisOfRecord: HumanObservation

##### Conservation status

Unknown

##### Notes

**Figure 17.**
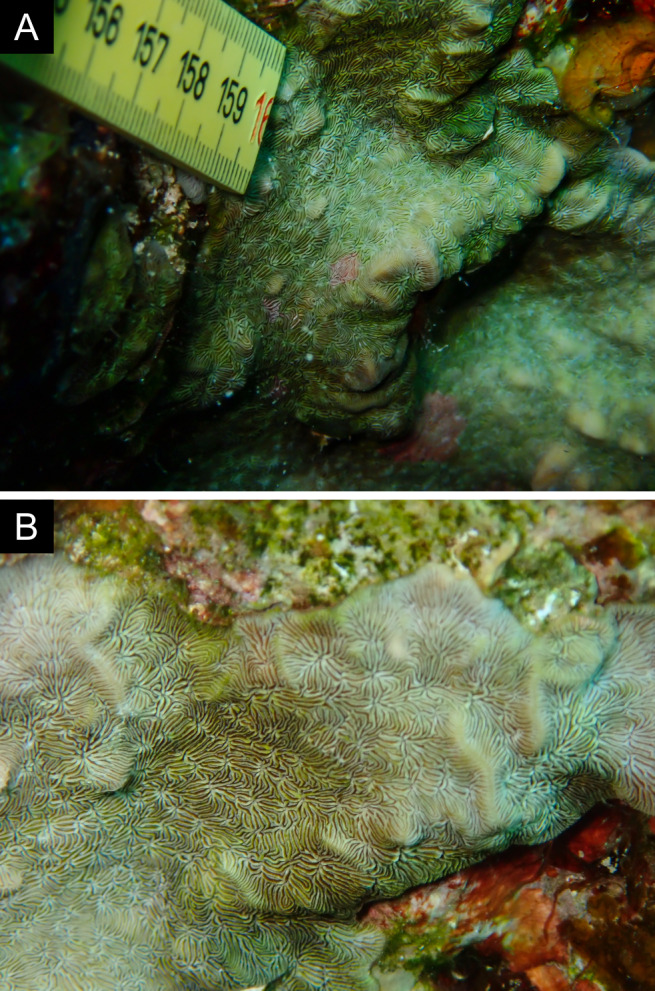
Amongst the *Pavona* colonies identified only to genus level, one morphotype (**A**—**B**) was distinguished. Photo credits: Theres Koch.

Fig. 17; one distinguished morphotype.

#### Pavona varians

(Verrill, 1864)

8C61C9CC-A03F-5710-9ACE-81C4EDC9477B

##### Materials

- **Type status:** Other material. **Occurrence:** occurrenceRemarks: Observed and photographed on a current-exposed shallow reef slope; occurrenceStatus: present; occurrenceID: C27173B7-E04B-55E3-8014-AEAA4B4C9483; **Taxon:** scientificNameID: urn:lsid:marinespecies.org:taxname:207303; scientificName: *Pavonavarians*; kingdom: Animalia; phylum: Cnidaria; class: Anthozoa; taxonRank: Species; **Location:** continent: Africa; waterBody: Red Sea; country: Egypt; stateProvince: Muhafazah al Bahr al Ahmar; locality: Current-exposed coral reef located on the coastline of Egypt (Mangrove Bay), 29 km south of the coastal city of El Qoseir; verbatimDepth: 4.3; verbatimLatitude: N25°52.209′; verbatimLongitude: E034°25.181′; decimalLatitude: 25.87015; decimalLongitude: 34.4196833; geodeticDatum: WGS84; **Event:** samplingEffort: 3 x 20 m Line Intercept Transects; eventDate: 24-10-2023; year: 2023; habitat: current-exposed shallow reef slope; **Record Level:** institutionCode: UNIVIE; collectionCode: Coral_Reef-Mangrove_Bay-Egypt-2023; basisOfRecord: HumanObservation
- **Type status:** Other material. **Occurrence:** occurrenceRemarks: Observed and photographed on a current-exposed deeper reef slope; occurrenceStatus: present; occurrenceID: 66E91763-4638-5D01-B3A1-6253E56BA1F7; **Taxon:** scientificNameID: urn:lsid:marinespecies.org:taxname:207303; scientificName: *Pavonavarians*; kingdom: Animalia; phylum: Cnidaria; class: Anthozoa; taxonRank: Species; **Location:** continent: Africa; waterBody: Red Sea; country: Egypt; stateProvince: Muhafazah al Bahr al Ahmar; locality: Current-exposed coral reef located on the coastline of Egypt (Mangrove Bay), 29 km south of the coastal city of El Qoseir; minimumDepthInMeters: 8.5; maximumDepthInMeters: 8.8; verbatimLatitude: N25°52.209′; verbatimLongitude: E034°25.181′; decimalLatitude: 25.87015; decimalLongitude: 34.4196833; geodeticDatum: WGS84; **Event:** samplingEffort: 3 x 20 m Line Intercept Transects; eventDate: 22-10-2023; year: 2023; habitat: current-exposed deeper reef slope; **Record Level:** institutionCode: UNIVIE; collectionCode: Coral_Reef-Mangrove_Bay-Egypt-2023; basisOfRecord: HumanObservation
- **Type status:** Other material. **Occurrence:** occurrenceRemarks: Observed and photographed on a sheltered reef edge; occurrenceStatus: present; occurrenceID: 5AB161BA-86A6-58AA-A8D5-1B378ADD5A8F; **Taxon:** scientificNameID: urn:lsid:marinespecies.org:taxname:207303; scientificName: *Pavonavarians*; kingdom: Animalia; phylum: Cnidaria; class: Anthozoa; taxonRank: Species; **Location:** continent: Africa; waterBody: Red Sea; country: Egypt; stateProvince: Muhafazah al Bahr al Ahmar; locality: Sheltered coral reef located on the coastline of Egypt (Mangrove Bay), 29 km south of the coastal city of El Qoseir; verbatimDepth: 1; verbatimLatitude: N25°52.183′; verbatimLongitude: E034°25.140′; decimalLatitude: 25.8697167; decimalLongitude: 34.419; geodeticDatum: WGS84; **Event:** samplingEffort: 3 x 20 m Line Intercept Transects; eventDate: 24-10-2023; year: 2023; habitat: sheltered reef edge; **Record Level:** institutionCode: UNIVIE; collectionCode: Coral_Reef-Mangrove_Bay-Egypt-2023; basisOfRecord: HumanObservation
- **Type status:** Other material. **Occurrence:** occurrenceRemarks: Observed and photographed on a sheltered shallow reef slope; occurrenceStatus: present; occurrenceID: 9A3063F3-E24A-5116-9CC2-BEB634D95DF6; **Taxon:** scientificNameID: urn:lsid:marinespecies.org:taxname:207303; scientificName: *Pavonavarians*; kingdom: Animalia; phylum: Cnidaria; class: Anthozoa; taxonRank: Species; **Location:** continent: Africa; waterBody: Red Sea; country: Egypt; stateProvince: Muhafazah al Bahr al Ahmar; locality: Sheltered coral reef located on the coastline of Egypt (Mangrove Bay), 29 km south of the coastal city of El Qoseir; minimumDepthInMeters: 3.7; maximumDepthInMeters: 4.75; verbatimLatitude: N25°52.183′; verbatimLongitude: E034°25.140′; decimalLatitude: 25.8697167; decimalLongitude: 34.419; geodeticDatum: WGS84; **Event:** samplingEffort: 3 x 20 m Line Intercept Transects; eventDate: 23-10-2023; year: 2023; habitat: sheltered shallow reef slope; **Record Level:** institutionCode: UNIVIE; collectionCode: Coral_Reef-Mangrove_Bay-Egypt-2023; basisOfRecord: HumanObservation
- **Type status:** Other material. **Occurrence:** occurrenceRemarks: Observed and photographed on a sheltered deeper reef slope; occurrenceStatus: present; occurrenceID: 7DE74121-8948-5C8D-9ECC-3B45FD0F4BAA; **Taxon:** scientificNameID: urn:lsid:marinespecies.org:taxname:207303; scientificName: *Pavonavarians*; kingdom: Animalia; phylum: Cnidaria; class: Anthozoa; taxonRank: Species; **Location:** continent: Africa; waterBody: Red Sea; country: Egypt; stateProvince: Muhafazah al Bahr al Ahmar; locality: Sheltered coral reef located on the coastline of Egypt (Mangrove Bay), 29 km south of the coastal city of El Qoseir; minimumDepthInMeters: 7.2; maximumDepthInMeters: 9.5; verbatimLatitude: N25°52.183′; verbatimLongitude: E034°25.140′; decimalLatitude: 25.8697167; decimalLongitude: 34.419; geodeticDatum: WGS84; **Event:** samplingEffort: 3 x 20 m Line Intercept Transects; eventDate: 25-10-2023; year: 2023; habitat: sheltered deeper reef slope; **Record Level:** institutionCode: UNIVIE; collectionCode: Coral_Reef-Mangrove_Bay-Egypt-2023; basisOfRecord: HumanObservation

##### Conservation status

Least Concern

##### Notes

Fig. 18 .

#### Astrocoeniidae

Koby, 1890

72621C38-E0A9-5DE2-97AD-0EB9FD0CA746

#### Stylocoeniella cf. guentheri

(Bassett-Smith, 1890)

E44E268F-0AD2-5F66-B1AD-E99993F8598D

##### Materials

- **Type status:** Other material. **Occurrence:** occurrenceRemarks: Observed and photographed on a current-exposed shallow reef slope; occurrenceStatus: present; occurrenceID: 460F4F9D-F269-5722-98F4-7E0C84A7C4EE; **Taxon:** scientificNameID: urn:lsid:marinespecies.org:taxname:206948; scientificName: Stylocoeniellacf.guentheri; kingdom: Animalia; phylum: Cnidaria; class: Anthozoa; taxonRank: Species; **Location:** continent: Africa; waterBody: Red Sea; country: Egypt; stateProvince: Muhafazah al Bahr al Ahmar; locality: Current-exposed coral reef located on the coastline of Egypt (Mangrove Bay), 29 km south of the coastal city of El Qoseir; verbatimDepth: 4.3; verbatimLatitude: N25°52.209′; verbatimLongitude: E034°25.181′; decimalLatitude: 25.87015; decimalLongitude: 34.4196833; geodeticDatum: WGS84; **Event:** samplingEffort: 3 x 20 m Line Intercept Transects; eventDate: 24-10-2023; year: 2023; habitat: current-exposed shallow reef slope; **Record Level:** institutionCode: UNIVIE; collectionCode: Coral_Reef-Mangrove_Bay-Egypt-2023; basisOfRecord: HumanObservation
- **Type status:** Other material. **Occurrence:** occurrenceRemarks: Observed and photographed on a current-exposed deeper reef slope; occurrenceStatus: present; occurrenceID: A7E97706-4963-5220-8F70-6E2970E43B4E; **Taxon:** scientificNameID: urn:lsid:marinespecies.org:taxname:206948; scientificName: Stylocoeniellacf.guentheri; kingdom: Animalia; phylum: Cnidaria; class: Anthozoa; taxonRank: Species; **Location:** continent: Africa; waterBody: Red Sea; country: Egypt; stateProvince: Muhafazah al Bahr al Ahmar; locality: Current-exposed coral reef located on the coastline of Egypt (Mangrove Bay), 29 km south of the coastal city of El Qoseir; minimumDepthInMeters: 8.5; maximumDepthInMeters: 8.8; verbatimLatitude: N25°52.209′; verbatimLongitude: E034°25.181′; decimalLatitude: 25.87015; decimalLongitude: 34.4196833; geodeticDatum: WGS84; **Event:** samplingEffort: 3 x 20 m Line Intercept Transects; eventDate: 22-10-2023; year: 2023; habitat: current-exposed deeper reef slope; **Record Level:** institutionCode: UNIVIE; collectionCode: Coral_Reef-Mangrove_Bay-Egypt-2023; basisOfRecord: HumanObservation

##### Conservation status

Least Concern

##### Notes

**Figure 19.**
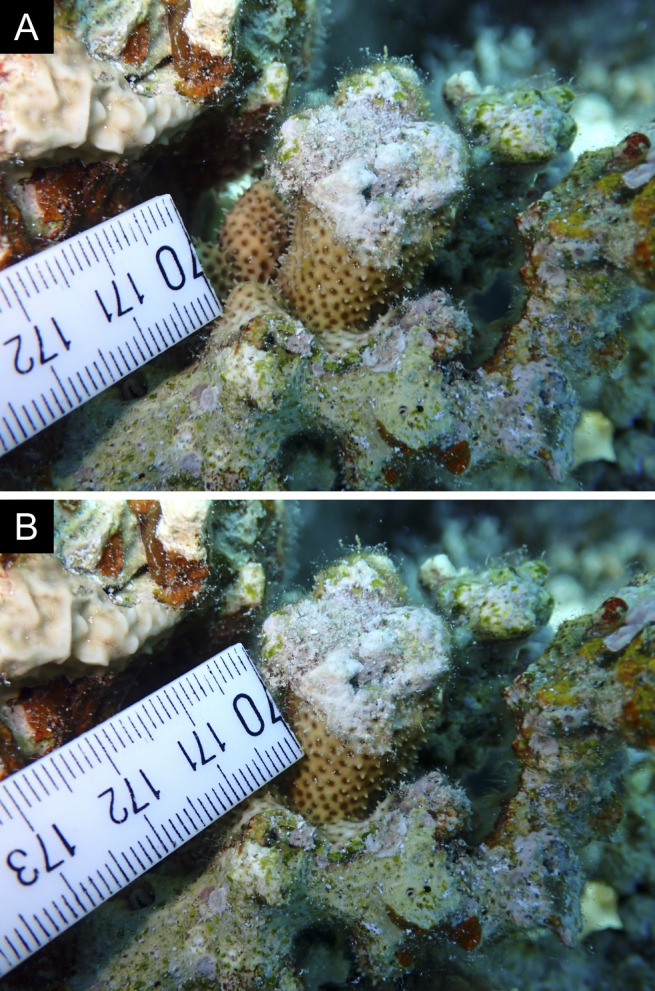
The growth form and appearance of Stylocoeniellacf.guentheri in the reef are shown in (**A**), while (**B**) provides a closer-up of the colony. Photo credits: Victor Sebastian Scharnhorst.

Fig. 19; possible alternative: *Stylocoeniellaarmata* (Ehrenberg, 1834).

#### Coscinaraeidae

Benzoni, Arrigoni, Stefani & Stolarski, 2012

3B0AF31B-088A-5B00-8565-08C31B92CD9F

#### Coscinaraea monile

(Forskål, 1775)

3461FA2A-E16F-588A-874D-E7B49903F363

##### Materials

- **Type status:** Other material. **Occurrence:** occurrenceRemarks: Observed and photographed on a current-exposed shallow reef slope; occurrenceStatus: present; occurrenceID: A5557BEF-6330-576F-8E45-D2DF03332C48; **Taxon:** scientificNameID: urn:lsid:marinespecies.org:taxname:207255; scientificName: *Coscinaraeamonile*; kingdom: Animalia; phylum: Cnidaria; class: Anthozoa; taxonRank: Species; **Location:** continent: Africa; waterBody: Red Sea; country: Egypt; stateProvince: Muhafazah al Bahr al Ahmar; locality: Current-exposed coral reef located on the coastline of Egypt (Mangrove Bay), 29 km south of the coastal city of El Qoseir; verbatimDepth: 4.3; verbatimLatitude: N25°52.209′; verbatimLongitude: E034°25.181′; decimalLatitude: 25.87015; decimalLongitude: 34.4196833; geodeticDatum: WGS84; **Event:** samplingEffort: 3 x 20 m Line Intercept Transects; eventDate: 22-10-2023; year: 2023; habitat: current-exposed shallow reef slope; **Record Level:** institutionCode: UNIVIE; collectionCode: Coral_Reef-Mangrove_Bay-Egypt-2023; basisOfRecord: HumanObservation
- **Type status:** Other material. **Occurrence:** occurrenceRemarks: Observed and photographed on a current-exposed deeper reef slope; occurrenceStatus: present; occurrenceID: FF0B2BDA-A64B-5DEA-98CA-98D4CDB5AA39; **Taxon:** scientificNameID: urn:lsid:marinespecies.org:taxname:207255; scientificName: *Coscinaraeamonile*; kingdom: Animalia; phylum: Cnidaria; class: Anthozoa; taxonRank: Species; **Location:** continent: Africa; waterBody: Red Sea; country: Egypt; stateProvince: Muhafazah al Bahr al Ahmar; locality: Current-exposed coral reef located on the coastline of Egypt (Mangrove Bay), 29 km south of the coastal city of El Qoseir; minimumDepthInMeters: 8.5; maximumDepthInMeters: 8.8; verbatimLatitude: N25°52.209′; verbatimLongitude: E034°25.181′; decimalLatitude: 25.87015; decimalLongitude: 34.4196833; geodeticDatum: WGS84; **Event:** samplingEffort: 3 x 20 m Line Intercept Transects; eventDate: 22-10-2023; year: 2023; habitat: current-exposed deeper reef slope; **Record Level:** institutionCode: UNIVIE; collectionCode: Coral_Reef-Mangrove_Bay-Egypt-2023; basisOfRecord: HumanObservation
- **Type status:** Other material. **Occurrence:** occurrenceRemarks: Observed and photographed on a sheltered deeper reef slope; occurrenceStatus: present; occurrenceID: E31D1F96-0940-5227-9668-147303D3CB80; **Taxon:** scientificNameID: urn:lsid:marinespecies.org:taxname:207255; scientificName: *Coscinaraeamonile*; kingdom: Animalia; phylum: Cnidaria; class: Anthozoa; taxonRank: Species; **Location:** continent: Africa; waterBody: Red Sea; country: Egypt; stateProvince: Muhafazah al Bahr al Ahmar; locality: Sheltered coral reef located on the coastline of Egypt (Mangrove Bay), 29 km south of the coastal city of El Qoseir; minimumDepthInMeters: 7.2; maximumDepthInMeters: 9.5; verbatimLatitude: N25°52.183′; verbatimLongitude: E034°25.140′; decimalLatitude: 25.8697167; decimalLongitude: 34.419; geodeticDatum: WGS84; **Event:** samplingEffort: 3 x 20 m Line Intercept Transects; eventDate: 25-10-2023; year: 2023; habitat: sheltered deeper reef slope; **Record Level:** institutionCode: UNIVIE; collectionCode: Coral_Reef-Mangrove_Bay-Egypt-2023; basisOfRecord: HumanObservation

##### Conservation status

Least Concern

##### Notes

Fig. 20 .

#### Dendrophylliidae

Gray, 1847

DBD51620-4654-5F8C-A247-D77E942E2AC2

#### Turbinaria mesenterina

(Lamarck, 1816)

5615B0A4-B628-5E99-9EF8-8E8372F3A1B4

##### Materials

- **Type status:** Other material. **Occurrence:** occurrenceRemarks: Observed and photographed on a current-exposed deeper reef slope; occurrenceStatus: present; occurrenceID: 2887E521-8C8A-508F-A0C9-7BCB9F6B62F6; **Taxon:** scientificNameID: urn:lsid:marinespecies.org:taxname:207511; scientificName: *Turbinariamesenterina*; kingdom: Animalia; phylum: Cnidaria; class: Anthozoa; taxonRank: Species; **Location:** continent: Africa; waterBody: Red Sea; country: Egypt; stateProvince: Muhafazah al Bahr al Ahmar; locality: Current-exposed coral reef located on the coastline of Egypt (Mangrove Bay), 29 km south of the coastal city of El Qoseir; minimumDepthInMeters: 8.5; maximumDepthInMeters: 8.8; verbatimLatitude: N25°52.209′; verbatimLongitude: E034°25.181′; decimalLatitude: 25.87015; decimalLongitude: 34.4196833; geodeticDatum: WGS84; **Event:** samplingEffort: 3 x 20 m Line Intercept Transects; eventDate: 22-10-2023; year: 2023; habitat: current-exposed deeper reef slope; **Record Level:** institutionCode: UNIVIE; collectionCode: Coral_Reef-Mangrove_Bay-Egypt-2023; basisOfRecord: HumanObservation

##### Conservation status

Vulnerable

##### Notes

Fig. 21 .

#### Euphylliidae

Milne Edwards & Haime, 1857

926DB9DD-E9FB-545C-9A3D-7EBAD85FE526

#### Galaxea fascicularis

(Linnaeus, 1767)

BA3D7091-1FF4-55EF-8352-6D650CCB586D

##### Materials

- **Type status:** Other material. **Occurrence:** occurrenceRemarks: Observed and photographed on a current-exposed shallow reef slope; occurrenceStatus: present; occurrenceID: 1AADB492-604B-540E-BB84-B2D7AF2985F8; **Taxon:** scientificNameID: urn:lsid:marinespecies.org:taxname:207366; scientificName: *Galaxeafascicularis*; kingdom: Animalia; phylum: Cnidaria; class: Anthozoa; taxonRank: Species; **Location:** continent: Africa; waterBody: Red Sea; country: Egypt; stateProvince: Muhafazah al Bahr al Ahmar; locality: Current-exposed coral reef located on the coastline of Egypt (Mangrove Bay), 29 km south of the coastal city of El Qoseir; verbatimDepth: 4.3; verbatimLatitude: N25°52.209′; verbatimLongitude: E034°25.181′; decimalLatitude: 25.87015; decimalLongitude: 34.4196833; geodeticDatum: WGS84; **Event:** samplingEffort: 3 x 20 m Line Intercept Transects; eventDate: 24-10-2023; year: 2023; habitat: current-exposed shallow reef slope; **Record Level:** institutionCode: UNIVIE; collectionCode: Coral_Reef-Mangrove_Bay-Egypt-2023; basisOfRecord: HumanObservation
- **Type status:** Other material. **Occurrence:** occurrenceRemarks: Observed and photographed on a current-exposed deeper reef slope; occurrenceStatus: present; occurrenceID: 624C41AB-F8F3-546B-86B1-72C53027B974; **Taxon:** scientificNameID: urn:lsid:marinespecies.org:taxname:207366; scientificName: *Galaxeafascicularis*; kingdom: Animalia; phylum: Cnidaria; class: Anthozoa; taxonRank: Species; **Location:** continent: Africa; waterBody: Red Sea; country: Egypt; stateProvince: Muhafazah al Bahr al Ahmar; locality: Current-exposed coral reef located on the coastline of Egypt (Mangrove Bay), 29 km south of the coastal city of El Qoseir; minimumDepthInMeters: 8.5; maximumDepthInMeters: 8.8; verbatimLatitude: N25°52.209′; verbatimLongitude: E034°25.181′; decimalLatitude: 25.87015; decimalLongitude: 34.4196833; geodeticDatum: WGS84; **Event:** samplingEffort: 3 x 20 m Line Intercept Transects; eventDate: 24-10-2023; year: 2023; habitat: current-exposed deeper reef slope; **Record Level:** institutionCode: UNIVIE; collectionCode: Coral_Reef-Mangrove_Bay-Egypt-2023; basisOfRecord: HumanObservation

##### Conservation status

Near Threatened

##### Notes

Fig. 22 .

#### Gyrosmilia interrupta

(Ehrenberg, 1834)

5DA26FBA-5F80-5B46-929E-248428314C3C

##### Materials

- **Type status:** Other material. **Occurrence:** occurrenceRemarks: Observed and photographed on a current-exposed deeper reef slope; occurrenceStatus: present; occurrenceID: 2C514366-C588-5AED-AD89-7F2F880CF306; **Taxon:** scientificNameID: urn:lsid:marinespecies.org:taxname:207496; scientificName: *Gyrosmiliainterrupta*; kingdom: Animalia; phylum: Cnidaria; class: Anthozoa; taxonRank: Species; **Location:** continent: Africa; waterBody: Red Sea; country: Egypt; stateProvince: Muhafazah al Bahr al Ahmar; locality: Current-exposed coral reef located on the coastline of Egypt (Mangrove Bay), 29 km south of the coastal city of El Qoseir; minimumDepthInMeters: 8.5; maximumDepthInMeters: 8.8; verbatimLatitude: N25°52.209′; verbatimLongitude: E034°25.181′; decimalLatitude: 25.87015; decimalLongitude: 34.4196833; geodeticDatum: WGS84; **Event:** samplingEffort: 3 x 20 m Line Intercept Transects; eventDate: 22-10-2023; year: 2023; habitat: current-exposed deeper reef slope; **Record Level:** institutionCode: UNIVIE; collectionCode: Coral_Reef-Mangrove_Bay-Egypt-2023; basisOfRecord: HumanObservation

##### Conservation status

Least Concern

##### Notes

Fig. 23 .

#### Fungiidae

Dana, 1846

19296977-6FC0-5AAE-8D25-C16435EAABFB

#### Ctenactis cf. crassa

(Dana, 1846)

C10B6D76-52F0-5B98-9CCE-DEB9246D04C5

##### Materials

- **Type status:** Other material. **Occurrence:** occurrenceRemarks: Observed and photographed on a current-exposed shallow reef slope; occurrenceStatus: present; occurrenceID: 836A8707-0412-5C0E-8924-858A9CDA304F; **Taxon:** scientificNameID: urn:lsid:marinespecies.org:taxname:288875; scientificName: Ctenactiscf.crassa; kingdom: Animalia; phylum: Cnidaria; class: Anthozoa; taxonRank: Species; **Location:** continent: Africa; waterBody: Red Sea; country: Egypt; stateProvince: Muhafazah al Bahr al Ahmar; locality: Current-exposed coral reef located on the coastline of Egypt (Mangrove Bay), 29 km south of the coastal city of El Qoseir; verbatimDepth: 4.3; verbatimLatitude: N25°52.209′; verbatimLongitude: E034°25.181′; decimalLatitude: 25.87015; decimalLongitude: 34.4196833; geodeticDatum: WGS84; **Event:** samplingEffort: 3 x 20 m Line Intercept Transects; eventDate: 24-10-2023; year: 2023; habitat: current-exposed shallow reef slope; **Record Level:** institutionCode: UNIVIE; collectionCode: Coral_Reef-Mangrove_Bay-Egypt-2023; basisOfRecord: HumanObservation
- **Type status:** Other material. **Occurrence:** occurrenceRemarks: Observed and photographed on a sheltered shallow reef slope; occurrenceStatus: present; occurrenceID: 9329969E-6C14-5291-9634-C40246F55659; **Taxon:** scientificNameID: urn:lsid:marinespecies.org:taxname:288875; scientificName: Ctenactiscf.crassa; kingdom: Animalia; phylum: Cnidaria; class: Anthozoa; taxonRank: Species; **Location:** continent: Africa; waterBody: Red Sea; country: Egypt; stateProvince: Muhafazah al Bahr al Ahmar; locality: Sheltered coral reef located on the coastline of Egypt (Mangrove Bay), 29 km south of the coastal city of El Qoseir; minimumDepthInMeters: 3.7; maximumDepthInMeters: 4.75; verbatimLatitude: N25°52.183′; verbatimLongitude: E034°25.140′; decimalLatitude: 25.8697167; decimalLongitude: 34.419; geodeticDatum: WGS84; **Event:** samplingEffort: 3 x 20 m Line Intercept Transects; eventDate: 25-10-2023; year: 2023; habitat: sheltered shallow reef slope; **Record Level:** institutionCode: UNIVIE; collectionCode: Coral_Reef-Mangrove_Bay-Egypt-2023; basisOfRecord: HumanObservation
- **Type status:** Other material. **Occurrence:** occurrenceRemarks: Observed and photographed on a sheltered deeper reef slope; occurrenceStatus: present; occurrenceID: EF372AE4-B5FC-55B8-943A-475F1361B85A; **Taxon:** scientificNameID: urn:lsid:marinespecies.org:taxname:288875; scientificName: Ctenactiscf.crassa; kingdom: Animalia; phylum: Cnidaria; class: Anthozoa; taxonRank: Species; **Location:** continent: Africa; waterBody: Red Sea; country: Egypt; stateProvince: Muhafazah al Bahr al Ahmar; locality: Sheltered coral reef located on the coastline of Egypt (Mangrove Bay), 29 km south of the coastal city of El Qoseir; minimumDepthInMeters: 7.2; maximumDepthInMeters: 9.5; verbatimLatitude: N25°52.183′; verbatimLongitude: E034°25.140′; decimalLatitude: 25.8697167; decimalLongitude: 34.419; geodeticDatum: WGS84; **Event:** samplingEffort: 3 x 20 m Line Intercept Transects; eventDate: 23-10-2023; year: 2023; habitat: sheltered deeper reef slope; **Record Level:** institutionCode: UNIVIE; collectionCode: Coral_Reef-Mangrove_Bay-Egypt-2023; basisOfRecord: HumanObservation

##### Conservation status

Least Concern

##### Notes

**Figure 24.**
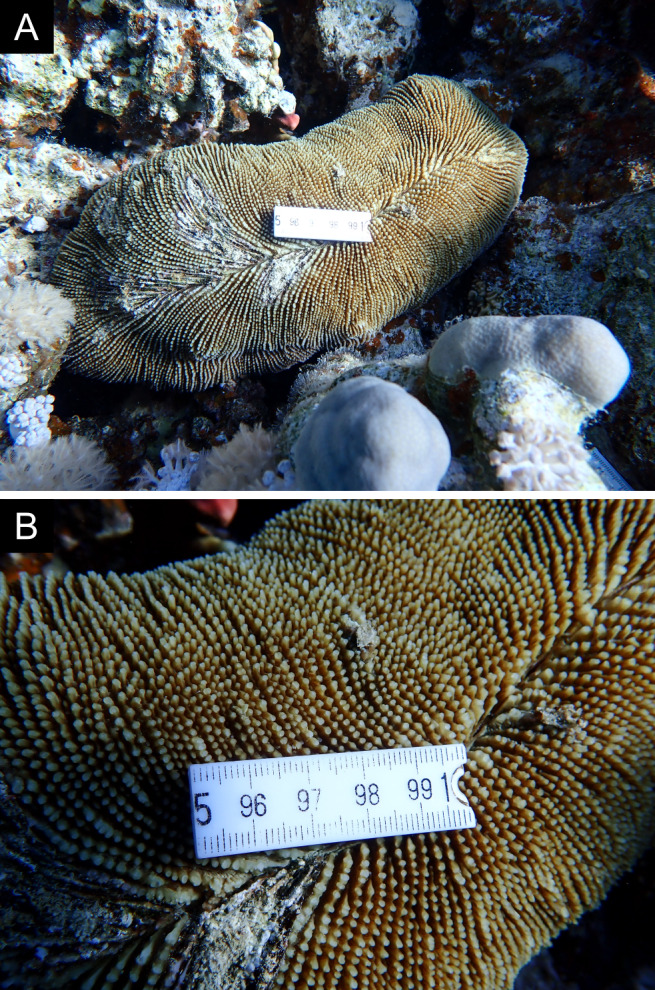
The growth form and appearance of Ctenactiscf.crassa in the reef are shown in (**A**), while (**B**) provides a close-up of the colony. Photo credits: Joseph Wallace Daurella.

Fig. 24; possible alternative: *Ctenactisechinata* (Pallas, 1766).

#### Leptastreidae

Rowlett, 2020

59D842E2-850E-5F02-9449-AD411FAE90B2

#### Leptastrea bottae

(Milne Edwards & Haime, 1849)

B3C705E0-1814-5FBA-91C6-08B93F86D78E

##### Materials

- **Type status:** Other material. **Occurrence:** occurrenceRemarks: Observed and photographed on a current-exposed deeper reef slope; occurrenceStatus: present; occurrenceID: 61EE2BBF-99F0-5005-9857-719FBE6AC2E1; **Taxon:** scientificNameID: urn:lsid:marinespecies.org:taxname:207476; scientificName: *Leptastreabottae*; kingdom: Animalia; phylum: Cnidaria; class: Anthozoa; taxonRank: Species; **Location:** continent: Africa; waterBody: Red Sea; country: Egypt; stateProvince: Muhafazah al Bahr al Ahmar; locality: Current-exposed coral reef located on the coastline of Egypt (Mangrove Bay), 29 km south of the coastal city of El Qoseir; minimumDepthInMeters: 8.5; maximumDepthInMeters: 8.8; verbatimLatitude: N25°52.209′; verbatimLongitude: E034°25.181′; decimalLatitude: 25.87015; decimalLongitude: 34.4196833; geodeticDatum: WGS84; **Event:** samplingEffort: 3 x 20 m Line Intercept Transects; eventDate: 22-10-2023; year: 2023; habitat: current-exposed deeper reef slope; **Record Level:** institutionCode: UNIVIE; collectionCode: Coral_Reef-Mangrove_Bay-Egypt-2023; basisOfRecord: HumanObservation

##### Conservation status

Near Threatened

##### Notes

Fig. 25 .

#### Leptastrea inaequalis

Klunzinger, 1879

FE1172F7-72B0-5FD6-BD14-9C9FD9D05D81

##### Materials

- **Type status:** Other material. **Occurrence:** occurrenceRemarks: Observed and photographed on a current-exposed shallow reef slope; occurrenceStatus: present; occurrenceID: E1BF60EB-9840-510D-AE58-BC617F549BE1; **Taxon:** scientificNameID: urn:lsid:marinespecies.org:taxname:207471; scientificName: *Leptastreainaequalis*; kingdom: Animalia; phylum: Cnidaria; class: Anthozoa; taxonRank: Species; **Location:** continent: Africa; waterBody: Red Sea; country: Egypt; stateProvince: Muhafazah al Bahr al Ahmar; locality: Current-exposed coral reef located on the coastline of Egypt (Mangrove Bay), 29 km south of the coastal city of El Qoseir; verbatimDepth: 4.3; verbatimLatitude: N25°52.209′; verbatimLongitude: E034°25.181′; decimalLatitude: 25.87015; decimalLongitude: 34.4196833; geodeticDatum: WGS84; **Event:** samplingEffort: 3 x 20 m Line Intercept Transects; eventDate: 22-10-2023; year: 2023; habitat: current-exposed shallow reef slope; **Record Level:** institutionCode: UNIVIE; collectionCode: Coral_Reef-Mangrove_Bay-Egypt-2023; basisOfRecord: HumanObservation

##### Conservation status

Near Threatened

##### Notes

Fig. 26 .

#### Leptastrea transversa

Klunzinger, 1879

7EC62BFF-FC11-54EF-866C-56DF39C09BFA

##### Materials

- **Type status:** Other material. **Occurrence:** occurrenceRemarks: Observed and photographed on a current-exposed shallow reef slope; occurrenceStatus: present; occurrenceID: C78474B0-BCBF-59C8-96B2-40DD9BCB7CC2; **Taxon:** scientificNameID: urn:lsid:marinespecies.org:taxname:207474; scientificName: *Leptastreatransversa*; kingdom: Animalia; phylum: Cnidaria; class: Anthozoa; taxonRank: Species; **Location:** continent: Africa; waterBody: Red Sea; country: Egypt; stateProvince: Muhafazah al Bahr al Ahmar; locality: Current-exposed coral reef located on the coastline of Egypt (Mangrove Bay), 29 km south of the coastal city of El Qoseir; verbatimDepth: 4.3; verbatimLatitude: N25°52.209′; verbatimLongitude: E034°25.181′; decimalLatitude: 25.87015; decimalLongitude: 34.4196833; geodeticDatum: WGS84; **Event:** samplingEffort: 3 x 20 m Line Intercept Transects; eventDate: 24-10-2023; year: 2023; habitat: current-exposed shallow reef slope; **Record Level:** institutionCode: UNIVIE; collectionCode: Coral_Reef-Mangrove_Bay-Egypt-2023; basisOfRecord: HumanObservation
- **Type status:** Other material. **Occurrence:** occurrenceRemarks: Observed and photographed on a current-exposed deeper reef slope; occurrenceStatus: present; occurrenceID: 458DDE44-A71A-5682-96D9-7BBE54E5145C; **Taxon:** scientificNameID: urn:lsid:marinespecies.org:taxname:207474; scientificName: *Leptastreatransversa*; kingdom: Animalia; phylum: Cnidaria; class: Anthozoa; taxonRank: Species; **Location:** continent: Africa; waterBody: Red Sea; country: Egypt; stateProvince: Muhafazah al Bahr al Ahmar; locality: Current-exposed coral reef located on the coastline of Egypt (Mangrove Bay), 29 km south of the coastal city of El Qoseir; minimumDepthInMeters: 8.5; maximumDepthInMeters: 8.8; verbatimLatitude: N25°52.209′; verbatimLongitude: E034°25.181′; decimalLatitude: 25.87015; decimalLongitude: 34.4196833; geodeticDatum: WGS84; **Event:** samplingEffort: 3 x 20 m Line Intercept Transects; eventDate: 22-10-2023; year: 2023; habitat: current-exposed deeper reef slope; **Record Level:** institutionCode: UNIVIE; collectionCode: Coral_Reef-Mangrove_Bay-Egypt-2023; basisOfRecord: HumanObservation
- **Type status:** Other material. **Occurrence:** occurrenceRemarks: Observed and photographed on a sheltered shallow reef slope; occurrenceStatus: present; occurrenceID: 7B251355-699E-5CA1-9E0C-E714ED56AF26; **Taxon:** scientificNameID: urn:lsid:marinespecies.org:taxname:207474; scientificName: *Leptastreatransversa*; kingdom: Animalia; phylum: Cnidaria; class: Anthozoa; taxonRank: Species; **Location:** continent: Africa; waterBody: Red Sea; country: Egypt; stateProvince: Muhafazah al Bahr al Ahmar; locality: Sheltered coral reef located on the coastline of Egypt (Mangrove Bay), 29 km south of the coastal city of El Qoseir; minimumDepthInMeters: 3.7; maximumDepthInMeters: 4.75; verbatimLatitude: N25°52.183′; verbatimLongitude: E034°25.140′; decimalLatitude: 25.8697167; decimalLongitude: 34.419; geodeticDatum: WGS84; **Event:** samplingEffort: 3 x 20 m Line Intercept Transects; eventDate: 23-10-2023; year: 2023; habitat: sheltered shallow reef slope; **Record Level:** institutionCode: UNIVIE; collectionCode: Coral_Reef-Mangrove_Bay-Egypt-2023; basisOfRecord: HumanObservation
- **Type status:** Other material. **Occurrence:** occurrenceRemarks: Observed and photographed on a sheltered deeper reef slope; occurrenceStatus: present; occurrenceID: 201307F3-5AD4-53A6-A1FB-12B4C679BDDE; **Taxon:** scientificNameID: urn:lsid:marinespecies.org:taxname:207474; scientificName: *Leptastreatransversa*; kingdom: Animalia; phylum: Cnidaria; class: Anthozoa; taxonRank: Species; **Location:** continent: Africa; waterBody: Red Sea; country: Egypt; stateProvince: Muhafazah al Bahr al Ahmar; locality: Sheltered coral reef located on the coastline of Egypt (Mangrove Bay), 29 km south of the coastal city of El Qoseir; minimumDepthInMeters: 7.2; maximumDepthInMeters: 9.5; verbatimLatitude: N25°52.183′; verbatimLongitude: E034°25.140′; decimalLatitude: 25.8697167; decimalLongitude: 34.419; geodeticDatum: WGS84; **Event:** samplingEffort: 3 x 20 m Line Intercept Transects; eventDate: 23-10-2023; year: 2023; habitat: sheltered deeper reef slope; **Record Level:** institutionCode: UNIVIE; collectionCode: Coral_Reef-Mangrove_Bay-Egypt-2023; basisOfRecord: HumanObservation

##### Conservation status

Least Concern

##### Notes

Fig. 27 .

#### Lobophylliidae

Dai & Horng, 2009

96F533BC-D237-5CFD-8324-D182273D1483

#### Acanthastrea hemprichii

(Ehrenberg, 1834)

5F7E99BE-AAFC-55EA-A8FC-6B0518192F46

##### Materials

- **Type status:** Other material. **Occurrence:** occurrenceRemarks: Observed and photographed on a current-exposed deeper reef slope; occurrenceStatus: present; occurrenceID: F2A16282-729D-505A-AF25-ECEC636A0730; **Taxon:** scientificNameID: urn:lsid:marinespecies.org:taxname:288878; scientificName: *Acanthastreahemprichii*; kingdom: Animalia; phylum: Cnidaria; class: Anthozoa; taxonRank: Species; **Location:** continent: Africa; waterBody: Red Sea; country: Egypt; stateProvince: Muhafazah al Bahr al Ahmar; locality: Current-exposed coral reef located on the coastline of Egypt (Mangrove Bay), 29 km south of the coastal city of El Qoseir; minimumDepthInMeters: 8.5; maximumDepthInMeters: 8.8; verbatimLatitude: N25°52.209′; verbatimLongitude: E034°25.181′; decimalLatitude: 25.87015; decimalLongitude: 34.4196833; geodeticDatum: WGS84; **Event:** samplingEffort: 3 x 20 m Line Intercept Transects; eventDate: 22-10-2023; year: 2023; habitat: current-exposed deeper reef slope; **Record Level:** institutionCode: UNIVIE; collectionCode: Coral_Reef-Mangrove_Bay-Egypt-2023; basisOfRecord: HumanObservation

##### Conservation status

Vulnerable

##### Notes

Fig. 28 .

#### Echinophyllia spp.

Klunzinger, 1879

4090A6B1-0015-589B-95D1-B26536399C48

##### Materials

- **Type status:** Other material. **Occurrence:** occurrenceRemarks: Observed and photographed on a current-exposed shallow reef slope; occurrenceStatus: present; occurrenceID: A83A305B-BCE2-5123-9366-EA9B72329302; **Taxon:** scientificNameID: urn:lsid:marinespecies.org:taxname:204799; scientificName: *Echinophyllia* spp.; kingdom: Animalia; phylum: Cnidaria; class: Anthozoa; taxonRank: Genus; **Location:** continent: Africa; waterBody: Red Sea; country: Egypt; stateProvince: Muhafazah al Bahr al Ahmar; locality: Current-exposed coral reef located on the coastline of Egypt (Mangrove Bay), 29 km south of the coastal city of El Qoseir; verbatimDepth: 4.3; verbatimLatitude: N25°52.209′; verbatimLongitude: E034°25.181′; decimalLatitude: 25.87015; decimalLongitude: 34.4196833; geodeticDatum: WGS84; **Event:** samplingEffort: 3 x 20 m Line Intercept Transects; eventDate: 22-10-2023; year: 2023; habitat: current-exposed shallow reef slope; **Record Level:** institutionCode: UNIVIE; collectionCode: Coral_Reef-Mangrove_Bay-Egypt-2023; basisOfRecord: HumanObservation

##### Conservation status

Unknown

##### Notes

**Figure 29.**
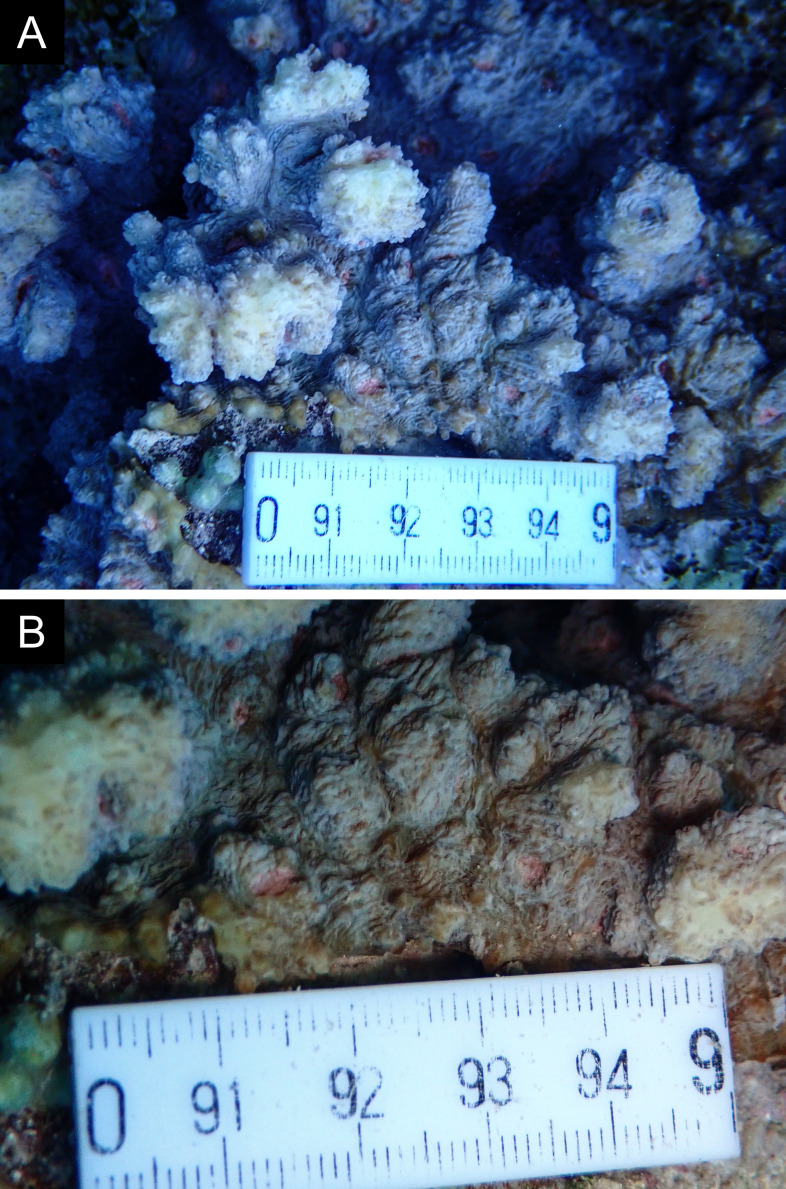
Amongst the *Echinophyllia* colonies identified only to genus level, one morphotype (**A**—**B**) was distinguished. Photo credits: Lewis Alan Jones.

Fig. 29; one distinguished morphotype.

#### Echinophyllia cf. spp.

Klunzinger, 1879

47362801-9628-5B4D-8070-146882D272BF

##### Materials

- **Type status:** Other material. **Occurrence:** occurrenceRemarks: Observed and photographed on a current-exposed deeper reef slope; occurrenceStatus: present; occurrenceID: 5C991BBC-2105-5B59-A41E-D85DA472175F; **Taxon:** scientificNameID: urn:lsid:marinespecies.org:taxname:204799; scientificName: *Echinophyllia* cf. sp.; kingdom: Animalia; phylum: Cnidaria; class: Anthozoa; taxonRank: Genus; **Location:** continent: Africa; waterBody: Red Sea; country: Egypt; stateProvince: Muhafazah al Bahr al Ahmar; locality: Current-exposed coral reef located on the coastline of Egypt (Mangrove Bay), 29 km south of the coastal city of El Qoseir; minimumDepthInMeters: 8.5; maximumDepthInMeters: 8.8; verbatimLatitude: N25°52.209′; verbatimLongitude: E034°25.181′; decimalLatitude: 25.87015; decimalLongitude: 34.4196833; geodeticDatum: WGS84; **Event:** samplingEffort: 3 x 20 m Line Intercept Transects; eventDate: 24-10-2023; year: 2023; habitat: current-exposed deeper reef slope; **Record Level:** institutionCode: UNIVIE; collectionCode: Coral_Reef-Mangrove_Bay-Egypt-2023; basisOfRecord: HumanObservation
- **Type status:** Other material. **Occurrence:** occurrenceRemarks: Observed and photographed on a sheltered deeper reef slope; occurrenceStatus: present; occurrenceID: ACE6C1D2-11F0-571E-ADE6-B0FC302C0559; **Taxon:** scientificNameID: urn:lsid:marinespecies.org:taxname:204799; scientificName: *Echinophyllia* cf. sp.; kingdom: Animalia; phylum: Cnidaria; class: Anthozoa; taxonRank: Genus; **Location:** continent: Africa; waterBody: Red Sea; country: Egypt; stateProvince: Muhafazah al Bahr al Ahmar; locality: Sheltered coral reef located on the coastline of Egypt (Mangrove Bay), 29 km south of the coastal city of El Qoseir; minimumDepthInMeters: 7.2; maximumDepthInMeters: 9.5; verbatimLatitude: N25°52.183′; verbatimLongitude: E034°25.140′; decimalLatitude: 25.8697167; decimalLongitude: 34.419; geodeticDatum: WGS84; **Event:** samplingEffort: 3 x 20 m Line Intercept Transects; eventDate: 25-10-2023; year: 2023; habitat: sheltered deeper reef slope; **Record Level:** institutionCode: UNIVIE; collectionCode: Coral_Reef-Mangrove_Bay-Egypt-2023; basisOfRecord: HumanObservation

##### Conservation status

Unknown

##### Notes

**Figure 30.**
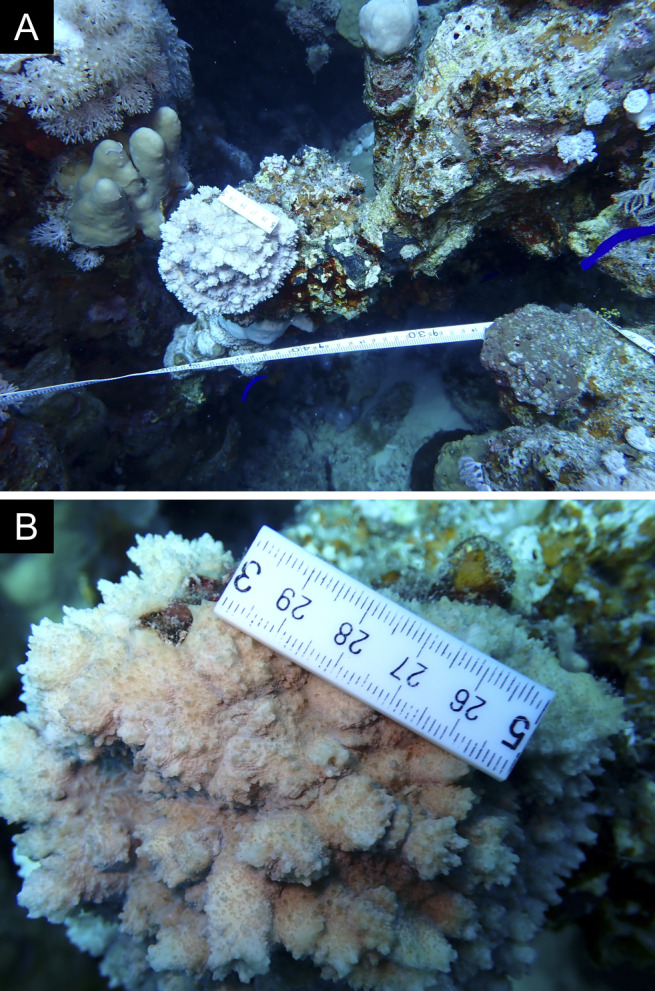
Amongst the *Echinophyllia* cf. colonies identified only to genus level, one morphotype (**A**—**B**) was distinguished. Photo credits: Victor Sebastian Scharnhorst.

Fig. 30; possible alternative: *Paraechinophyllia* sp. Arrigoni, Benzoni & Stolarski, 2019.

#### Oxypora sp.

Saville Kent, 1871

345B6D02-5730-5AE9-AC49-F8302D6B27AC

##### Materials

- **Type status:** Other material. **Occurrence:** occurrenceRemarks: Observed and photographed on a current-exposed deeper reef slope; occurrenceStatus: present; occurrenceID: C7F770A8-F6EE-5FE7-B63E-3C691A15B262; **Taxon:** scientificNameID: urn:lsid:marinespecies.org:taxname:205536; scientificName: *Oxypora* sp.; kingdom: Animalia; phylum: Cnidaria; class: Anthozoa; taxonRank: Genus; **Location:** continent: Africa; waterBody: Red Sea; country: Egypt; stateProvince: Muhafazah al Bahr al Ahmar; locality: Current-exposed coral reef located on the coastline of Egypt (Mangrove Bay), 29 km south of the coastal city of El Qoseir; minimumDepthInMeters: 8.5; maximumDepthInMeters: 8.8; verbatimLatitude: N25°52.209′; verbatimLongitude: E034°25.181′; decimalLatitude: 25.87015; decimalLongitude: 34.4196833; geodeticDatum: WGS84; **Event:** samplingEffort: 3 x 20 m Line Intercept Transects; eventDate: 22-10-2023; year: 2023; habitat: current-exposed deeper reef slope; **Record Level:** institutionCode: UNIVIE; collectionCode: Coral_Reef-Mangrove_Bay-Egypt-2023; basisOfRecord: HumanObservation

##### Conservation status

Unknown

##### Notes

**Figure 31.**
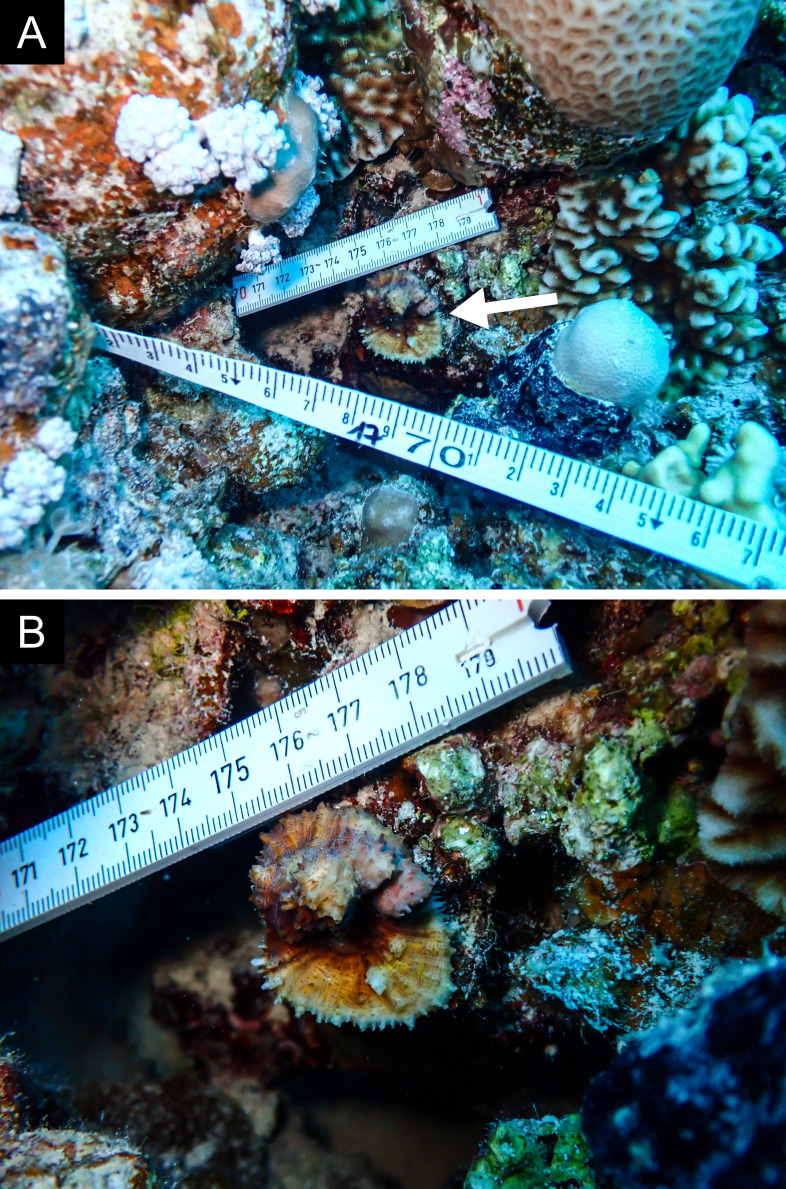
The growth form and appearance of the only *Oxypora* colony found is represented by one morphotype (**A**—**B**). Photo credits: Victor Sebastian Scharnhorst.

Fig. 31; one distinguished morphotype.

#### Merulinidae

Milne Edwards & Haime, 1857

81DBF412-82C8-543B-9FFC-7CCDBB601C0B

#### Cyphastrea chalcidicum

(Forskål, 1775)

305F2CB5-B284-5B33-8ACE-470FD545B3D8

##### Materials

- **Type status:** Other material. **Occurrence:** occurrenceRemarks: Observed and photographed on a current-exposed shallow reef slope; occurrenceStatus: present; occurrenceID: 07904C90-FA9D-5189-BC2B-865FCCAE32BF; **Taxon:** scientificNameID: urn:lsid:marinespecies.org:taxname:207415; scientificName: *Cyphastreachalcidicum*; kingdom: Animalia; phylum: Cnidaria; class: Anthozoa; taxonRank: Species; **Location:** continent: Africa; waterBody: Red Sea; country: Egypt; stateProvince: Muhafazah al Bahr al Ahmar; locality: Current-exposed coral reef located on the coastline of Egypt (Mangrove Bay), 29 km south of the coastal city of El Qoseir; verbatimDepth: 4.3; verbatimLatitude: N25°52.209′; verbatimLongitude: E034°25.181′; decimalLatitude: 25.87015; decimalLongitude: 34.4196833; geodeticDatum: WGS84; **Event:** samplingEffort: 3 x 20 m Line Intercept Transects; eventDate: 22-10-2023; year: 2023; habitat: current-exposed shallow reef slope; **Record Level:** institutionCode: UNIVIE; collectionCode: Coral_Reef-Mangrove_Bay-Egypt-2023; basisOfRecord: HumanObservation
- **Type status:** Other material. **Occurrence:** occurrenceRemarks: Observed and photographed on a current-exposed deeper reef slope; occurrenceStatus: present; occurrenceID: A5EEC132-B1E6-510D-A745-C7C5D5EE4C27; **Taxon:** scientificNameID: urn:lsid:marinespecies.org:taxname:207415; scientificName: *Cyphastreachalcidicum*; kingdom: Animalia; phylum: Cnidaria; class: Anthozoa; taxonRank: Species; **Location:** continent: Africa; waterBody: Red Sea; country: Egypt; stateProvince: Muhafazah al Bahr al Ahmar; locality: Current-exposed coral reef located on the coastline of Egypt (Mangrove Bay), 29 km south of the coastal city of El Qoseir; minimumDepthInMeters: 8.5; maximumDepthInMeters: 8.8; verbatimLatitude: N25°52.209′; verbatimLongitude: E034°25.181′; decimalLatitude: 25.87015; decimalLongitude: 34.4196833; geodeticDatum: WGS84; **Event:** samplingEffort: 3 x 20 m Line Intercept Transects; eventDate: 22-10-2023; year: 2023; habitat: current-exposed deeper reef slope; **Record Level:** institutionCode: UNIVIE; collectionCode: Coral_Reef-Mangrove_Bay-Egypt-2023; basisOfRecord: HumanObservation
- **Type status:** Other material. **Occurrence:** occurrenceRemarks: Observed and photographed on a sheltered reef edge; occurrenceStatus: present; occurrenceID: CCCA5F78-A732-5F82-942F-2B63EBBB33CC; **Taxon:** scientificNameID: urn:lsid:marinespecies.org:taxname:207415; scientificName: *Cyphastreachalcidicum*; kingdom: Animalia; phylum: Cnidaria; class: Anthozoa; taxonRank: Species; **Location:** continent: Africa; waterBody: Red Sea; country: Egypt; stateProvince: Muhafazah al Bahr al Ahmar; locality: Sheltered coral reef located on the coastline of Egypt (Mangrove Bay), 29 km south of the coastal city of El Qoseir; verbatimDepth: 1; verbatimLatitude: N25°52.183′; verbatimLongitude: E034°25.140′; decimalLatitude: 25.8697167; decimalLongitude: 34.419; geodeticDatum: WGS84; **Event:** samplingEffort: 3 x 20 m Line Intercept Transects; eventDate: 20-10-2023; year: 2023; habitat: sheltered reef edge; **Record Level:** institutionCode: UNIVIE; collectionCode: Coral_Reef-Mangrove_Bay-Egypt-2023; basisOfRecord: HumanObservation
- **Type status:** Other material. **Occurrence:** occurrenceRemarks: Observed and photographed on a sheltered shallow reef slope; occurrenceStatus: present; occurrenceID: C552D0C9-0C74-5132-8723-9781807FA103; **Taxon:** scientificNameID: urn:lsid:marinespecies.org:taxname:207415; scientificName: *Cyphastreachalcidicum*; kingdom: Animalia; phylum: Cnidaria; class: Anthozoa; taxonRank: Species; **Location:** continent: Africa; waterBody: Red Sea; country: Egypt; stateProvince: Muhafazah al Bahr al Ahmar; locality: Sheltered coral reef located on the coastline of Egypt (Mangrove Bay), 29 km south of the coastal city of El Qoseir; minimumDepthInMeters: 3.7; maximumDepthInMeters: 4.75; verbatimLatitude: N25°52.183′; verbatimLongitude: E034°25.140′; decimalLatitude: 25.8697167; decimalLongitude: 34.419; geodeticDatum: WGS84; **Event:** samplingEffort: 3 x 20 m Line Intercept Transects; eventDate: 25-10-2023; year: 2023; habitat: sheltered shallow reef slope; **Record Level:** institutionCode: UNIVIE; collectionCode: Coral_Reef-Mangrove_Bay-Egypt-2023; basisOfRecord: HumanObservation

##### Conservation status

Least Concern

##### Notes

Fig. 32 .

#### Cyphastrea kausti

Bouwmeester & Benzoni, 2015

C32D72D6-F517-58AE-855D-C15506B51092

##### Materials

- **Type status:** Other material. **Occurrence:** occurrenceRemarks: Observed and photographed on a current-exposed shallow reef slope; occurrenceStatus: present; occurrenceID: C0E5006C-1C14-5ED3-95C1-E6C348AAD93F; **Taxon:** scientificNameID: urn:lsid:marinespecies.org:taxname:843628; scientificName: *Cyphastreakausti*; kingdom: Animalia; phylum: Cnidaria; class: Anthozoa; taxonRank: Species; **Location:** continent: Africa; waterBody: Red Sea; country: Egypt; stateProvince: Muhafazah al Bahr al Ahmar; locality: Current-exposed coral reef located on the coastline of Egypt (Mangrove Bay), 29 km south of the coastal city of El Qoseir; verbatimDepth: 4.3; verbatimLatitude: N25°52.209′; verbatimLongitude: E034°25.181′; decimalLatitude: 25.87015; decimalLongitude: 34.4196833; geodeticDatum: WGS84; **Event:** samplingEffort: 3 x 20 m Line Intercept Transects; eventDate: 24-10-2023; year: 2023; habitat: current-exposed shallow reef slope; **Record Level:** institutionCode: UNIVIE; collectionCode: Coral_Reef-Mangrove_Bay-Egypt-2023; basisOfRecord: HumanObservation
- **Type status:** Other material. **Occurrence:** occurrenceRemarks: Observed and photographed on a current-exposed deeper reef slope; occurrenceStatus: present; occurrenceID: 7BCB45C0-BDF8-50B5-B7C5-296961953F6E; **Taxon:** scientificNameID: urn:lsid:marinespecies.org:taxname:843628; scientificName: *Cyphastreakausti*; kingdom: Animalia; phylum: Cnidaria; class: Anthozoa; taxonRank: Species; **Location:** continent: Africa; waterBody: Red Sea; country: Egypt; stateProvince: Muhafazah al Bahr al Ahmar; locality: Current-exposed coral reef located on the coastline of Egypt (Mangrove Bay), 29 km south of the coastal city of El Qoseir; minimumDepthInMeters: 8.5; maximumDepthInMeters: 8.8; verbatimLatitude: N25°52.209′; verbatimLongitude: E034°25.181′; decimalLatitude: 25.87015; decimalLongitude: 34.4196833; geodeticDatum: WGS84; **Event:** samplingEffort: 3 x 20 m Line Intercept Transects; eventDate: 22-10-2023; year: 2023; habitat: current-exposed deeper reef slope; **Record Level:** institutionCode: UNIVIE; collectionCode: Coral_Reef-Mangrove_Bay-Egypt-2023; basisOfRecord: HumanObservation
- **Type status:** Other material. **Occurrence:** occurrenceRemarks: Observed and photographed on a sheltered shallow reef slope; occurrenceStatus: present; occurrenceID: 52E00F26-7C3A-58E3-B217-5D450C8F6462; **Taxon:** scientificNameID: urn:lsid:marinespecies.org:taxname:843628; scientificName: *Cyphastreakausti*; kingdom: Animalia; phylum: Cnidaria; class: Anthozoa; taxonRank: Species; **Location:** continent: Africa; waterBody: Red Sea; country: Egypt; stateProvince: Muhafazah al Bahr al Ahmar; locality: Sheltered coral reef located on the coastline of Egypt (Mangrove Bay), 29 km south of the coastal city of El Qoseir; minimumDepthInMeters: 3.7; maximumDepthInMeters: 4.75; verbatimLatitude: N25°52.183′; verbatimLongitude: E034°25.140′; decimalLatitude: 25.8697167; decimalLongitude: 34.419; geodeticDatum: WGS84; **Event:** samplingEffort: 3 x 20 m Line Intercept Transects; eventDate: 25-10-2023; year: 2023; habitat: sheltered shallow reef slope; **Record Level:** institutionCode: UNIVIE; collectionCode: Coral_Reef-Mangrove_Bay-Egypt-2023; basisOfRecord: HumanObservation

##### Conservation status

Unknown

##### Notes

Fig. 33 .

#### Cyphastrea magna

Benzoni & Arrigoni, 2017

D30AA6DA-3B82-5E20-89D2-CB723A2D648C

##### Materials

- **Type status:** Other material. **Occurrence:** occurrenceRemarks: Observed and photographed on a current-exposed reef edge; occurrenceStatus: present; occurrenceID: F017D0CB-9629-5D62-90C5-915E7BC52883; **Taxon:** scientificNameID: urn:lsid:marinespecies.org:taxname:990872; scientificName: *Cyphastreamagna*; kingdom: Animalia; phylum: Cnidaria; class: Anthozoa; taxonRank: Species; **Location:** continent: Africa; waterBody: Red Sea; country: Egypt; stateProvince: Muhafazah al Bahr al Ahmar; locality: Current-exposed coral reef located on the coastline of Egypt (Mangrove Bay), 29 km south of the coastal city of El Qoseir; verbatimDepth: 1; verbatimLatitude: N25°52.209′; verbatimLongitude: E034°25.181′; decimalLatitude: 25.87015; decimalLongitude: 34.4196833; geodeticDatum: WGS84; **Event:** samplingEffort: 3 x 20 m Line Intercept Transects; eventDate: 22-10-2023; year: 2023; habitat: current-exposed reef edge; **Record Level:** institutionCode: UNIVIE; collectionCode: Coral_Reef-Mangrove_Bay-Egypt-2023; basisOfRecord: HumanObservation
- **Type status:** Other material. **Occurrence:** occurrenceRemarks: Observed and photographed on a sheltered shallow reef slope; occurrenceStatus: present; occurrenceID: B72BD2D0-A730-5D77-885C-CBF54D3B1627; **Taxon:** scientificNameID: urn:lsid:marinespecies.org:taxname:990872; scientificName: *Cyphastreamagna*; kingdom: Animalia; phylum: Cnidaria; class: Anthozoa; taxonRank: Species; **Location:** continent: Africa; waterBody: Red Sea; country: Egypt; stateProvince: Muhafazah al Bahr al Ahmar; locality: Sheltered coral reef located on the coastline of Egypt (Mangrove Bay), 29 km south of the coastal city of El Qoseir; minimumDepthInMeters: 3.7; maximumDepthInMeters: 4.75; verbatimLatitude: N25°52.183′; verbatimLongitude: E034°25.140′; decimalLatitude: 25.8697167; decimalLongitude: 34.419; geodeticDatum: WGS84; **Event:** samplingEffort: 3 x 20 m Line Intercept Transects; eventDate: 25-10-2023; year: 2023; habitat: sheltered shallow reef slope; **Record Level:** institutionCode: UNIVIE; collectionCode: Coral_Reef-Mangrove_Bay-Egypt-2023; basisOfRecord: HumanObservation

##### Conservation status

Unknown

##### Notes

Fig. 34 .

#### Cyphastrea microphthalma

(Lamarck, 1816)

7AE9E801-C279-5D64-9969-F3121F985F65

##### Materials

- **Type status:** Other material. **Occurrence:** occurrenceRemarks: Observed and photographed on a current-exposed shallow reef slope; occurrenceStatus: present; occurrenceID: C8244BB3-6017-52A8-A247-6F70DECC9AC0; **Taxon:** scientificNameID: urn:lsid:marinespecies.org:taxname:207416; scientificName: *Cyphastreamicrophthalma*; kingdom: Animalia; phylum: Cnidaria; class: Anthozoa; taxonRank: Species; **Location:** continent: Africa; waterBody: Red Sea; country: Egypt; stateProvince: Muhafazah al Bahr al Ahmar; locality: Current-exposed coral reef located on the coastline of Egypt (Mangrove Bay), 29 km south of the coastal city of El Qoseir; verbatimDepth: 4.3; verbatimLatitude: N25°52.209′; verbatimLongitude: E034°25.181′; decimalLatitude: 25.87015; decimalLongitude: 34.4196833; geodeticDatum: WGS84; **Event:** samplingEffort: 3 x 20 m Line Intercept Transects; eventDate: 24-10-2023; year: 2023; habitat: current-exposed shallow reef slope; **Record Level:** institutionCode: UNIVIE; collectionCode: Coral_Reef-Mangrove_Bay-Egypt-2023; basisOfRecord: HumanObservation
- **Type status:** Other material. **Occurrence:** occurrenceRemarks: Observed and photographed on a current-exposed deeper reef slope; occurrenceStatus: present; occurrenceID: E787782E-A3DF-5446-A940-284B2878DADB; **Taxon:** scientificNameID: urn:lsid:marinespecies.org:taxname:207416; scientificName: *Cyphastreamicrophthalma*; kingdom: Animalia; phylum: Cnidaria; class: Anthozoa; taxonRank: Species; **Location:** continent: Africa; waterBody: Red Sea; country: Egypt; stateProvince: Muhafazah al Bahr al Ahmar; locality: Current-exposed coral reef located on the coastline of Egypt (Mangrove Bay), 29 km south of the coastal city of El Qoseir; minimumDepthInMeters: 8.5; maximumDepthInMeters: 8.8; verbatimLatitude: N25°52.209′; verbatimLongitude: E034°25.181′; decimalLatitude: 25.87015; decimalLongitude: 34.4196833; geodeticDatum: WGS84; **Event:** samplingEffort: 3 x 20 m Line Intercept Transects; eventDate: 22-10-2023; year: 2023; habitat: current-exposed deeper reef slope; **Record Level:** institutionCode: UNIVIE; collectionCode: Coral_Reef-Mangrove_Bay-Egypt-2023; basisOfRecord: HumanObservation
- **Type status:** Other material. **Occurrence:** occurrenceRemarks: Observed and photographed on a sheltered deeper reef slope; occurrenceStatus: present; occurrenceID: 8BE39145-04A0-50F4-8F50-219C9579BF2E; **Taxon:** scientificNameID: urn:lsid:marinespecies.org:taxname:207416; scientificName: *Cyphastreamicrophthalma*; kingdom: Animalia; phylum: Cnidaria; class: Anthozoa; taxonRank: Species; **Location:** continent: Africa; waterBody: Red Sea; country: Egypt; stateProvince: Muhafazah al Bahr al Ahmar; locality: Sheltered coral reef located on the coastline of Egypt (Mangrove Bay), 29 km south of the coastal city of El Qoseir; minimumDepthInMeters: 7.2; maximumDepthInMeters: 9.5; verbatimLatitude: N25°52.183′; verbatimLongitude: E034°25.140′; decimalLatitude: 25.8697167; decimalLongitude: 34.419; geodeticDatum: WGS84; **Event:** samplingEffort: 3 x 20 m Line Intercept Transects; eventDate: 23-10-2023; year: 2023; habitat: sheltered deeper reef slope; **Record Level:** institutionCode: UNIVIE; collectionCode: Coral_Reef-Mangrove_Bay-Egypt-2023; basisOfRecord: HumanObservation

##### Conservation status

Least Concern

##### Notes

Fig. 35 .

#### Cyphastrea spp.

Milne Edwards & Haime, 1849

A4751F6D-DA31-5158-AF25-53FC761F07FA

##### Materials

- **Type status:** Other material. **Occurrence:** occurrenceRemarks: Observed and photographed on a current-exposed shallow reef slope; occurrenceStatus: present; occurrenceID: 36D77519-5E4A-5B71-93E0-77DD3EABC1FB; **Taxon:** scientificNameID: urn:lsid:marinespecies.org:taxname:206488; scientificName: *Cyphastrea* sp.; kingdom: Animalia; phylum: Cnidaria; class: Anthozoa; taxonRank: Genus; **Location:** continent: Africa; waterBody: Red Sea; country: Egypt; stateProvince: Muhafazah al Bahr al Ahmar; locality: Current-exposed coral reef located on the coastline of Egypt (Mangrove Bay), 29 km south of the coastal city of El Qoseir; verbatimDepth: 4.3; verbatimLatitude: N25°52.209′; verbatimLongitude: E034°25.181′; decimalLatitude: 25.87015; decimalLongitude: 34.4196833; geodeticDatum: WGS84; **Event:** samplingEffort: 3 x 20 m Line Intercept Transects; eventDate: 24-10-2023; year: 2023; habitat: current-exposed shallow reef slope; **Record Level:** institutionCode: UNIVIE; collectionCode: Coral_Reef-Mangrove_Bay-Egypt-2023; basisOfRecord: HumanObservation
- **Type status:** Other material. **Occurrence:** occurrenceRemarks: Observed and photographed on a current-exposed deeper reef slope; occurrenceStatus: present; occurrenceID: 7175C2FA-434C-5EE0-B36B-FAEFBDA7769D; **Taxon:** scientificNameID: urn:lsid:marinespecies.org:taxname:206488; scientificName: *Cyphastrea* sp.; kingdom: Animalia; phylum: Cnidaria; class: Anthozoa; taxonRank: Genus; **Location:** continent: Africa; waterBody: Red Sea; country: Egypt; stateProvince: Muhafazah al Bahr al Ahmar; locality: Current-exposed coral reef located on the coastline of Egypt (Mangrove Bay), 29 km south of the coastal city of El Qoseir; minimumDepthInMeters: 8.5; maximumDepthInMeters: 8.8; verbatimLatitude: N25°52.209′; verbatimLongitude: E034°25.181′; decimalLatitude: 25.87015; decimalLongitude: 34.4196833; geodeticDatum: WGS84; **Event:** samplingEffort: 3 x 20 m Line Intercept Transects; eventDate: 22-10-2023; year: 2023; habitat: current-exposed deeper reef slope; **Record Level:** institutionCode: UNIVIE; collectionCode: Coral_Reef-Mangrove_Bay-Egypt-2023; basisOfRecord: HumanObservation

##### Conservation status

Unknown

##### Notes

**Figure 36.**
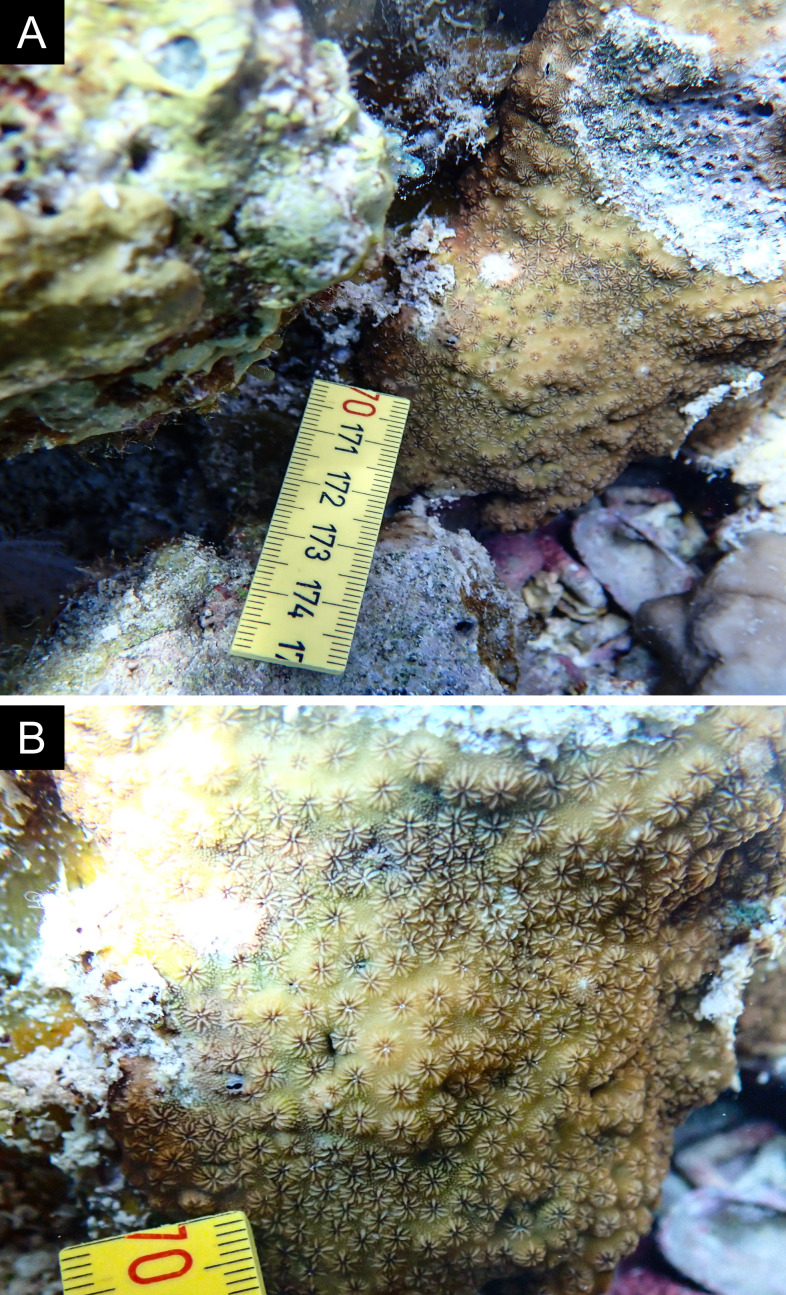
Amongst the *Cyphastrea* colonies identified only to genus level, one morphotype (**A**—**B**) was distinguished. Photo credits: Joseph Wallace Daurella.

Fig. 36; one distinguished morphotype.

#### Dipsastraea danai

(Milne Edwards & Haime, 1857)

A064CC9F-A101-5E05-8E4A-C5B47CE3CD5C

##### Materials

- **Type status:** Other material. **Occurrence:** occurrenceRemarks: Observed and photographed on a current-exposed shallow reef slope; occurrenceStatus: present; occurrenceID: 11FDDE2E-0973-5BE0-B64B-B030A5E29AFF; **Taxon:** scientificNameID: urn:lsid:marinespecies.org:taxname:758238; scientificName: *Dipsastraeadanai*; kingdom: Animalia; phylum: Cnidaria; class: Anthozoa; taxonRank: Species; **Location:** continent: Africa; waterBody: Red Sea; country: Egypt; stateProvince: Muhafazah al Bahr al Ahmar; locality: Current-exposed coral reef located on the coastline of Egypt (Mangrove Bay), 29 km south of the coastal city of El Qoseir; verbatimDepth: 4.3; verbatimLatitude: N25°52.209′; verbatimLongitude: E034°25.181′; decimalLatitude: 25.87015; decimalLongitude: 34.4196833; geodeticDatum: WGS84; **Event:** samplingEffort: 3 x 20 m Line Intercept Transects; eventDate: 24-10-2023; year: 2023; habitat: current-exposed shallow reef slope; **Record Level:** institutionCode: UNIVIE; collectionCode: Coral_Reef-Mangrove_Bay-Egypt-2023; basisOfRecord: HumanObservation

##### Conservation status

Least Concern

##### Notes

Fig. 37 .

#### Dipsastraea faviaformis

(Veron, 2000)

D8FE75D0-5909-5640-994B-52863A6ECDC9

##### Materials

- **Type status:** Other material. **Occurrence:** occurrenceRemarks: Observed and photographed on a current-exposed deeper reef slope; occurrenceStatus: present; occurrenceID: 58917073-E857-5FC9-AEA0-D42C0AFBF37F; **Taxon:** scientificNameID: urn:lsid:marinespecies.org:taxname:888171; scientificName: *Dipsastraeafaviaformis*; kingdom: Animalia; phylum: Cnidaria; class: Anthozoa; taxonRank: Species; **Location:** continent: Africa; waterBody: Red Sea; country: Egypt; stateProvince: Muhafazah al Bahr al Ahmar; locality: Current-exposed coral reef located on the coastline of Egypt (Mangrove Bay), 29 km south of the coastal city of El Qoseir; minimumDepthInMeters: 8.5; maximumDepthInMeters: 8.8; verbatimLatitude: N25°52.209′; verbatimLongitude: E034°25.181′; decimalLatitude: 25.87015; decimalLongitude: 34.4196833; geodeticDatum: WGS84; **Event:** samplingEffort: 3 x 20 m Line Intercept Transects; eventDate: 24-10-2023; year: 2023; habitat: current-exposed deeper reef slope; **Record Level:** institutionCode: UNIVIE; collectionCode: Coral_Reef-Mangrove_Bay-Egypt-2023; basisOfRecord: HumanObservation

##### Conservation status

Vulnerable

##### Notes

Fig. 38 .

#### Dipsastraea laxa

(Klunzinger, 1879)

E651141F-31E6-59B9-A936-27792746460C

##### Materials

- **Type status:** Other material. **Occurrence:** occurrenceRemarks: Observed and photographed on a current-exposed deeper reef slope; occurrenceStatus: present; occurrenceID: 2963DAD9-1AD2-518F-A8C1-6F11F0FB13A3; **Taxon:** scientificNameID: urn:lsid:marinespecies.org:taxname:758235; scientificName: *Dipsastraealaxa*; kingdom: Animalia; phylum: Cnidaria; class: Anthozoa; taxonRank: Species; **Location:** continent: Africa; waterBody: Red Sea; country: Egypt; stateProvince: Muhafazah al Bahr al Ahmar; locality: Current-exposed coral reef located on the coastline of Egypt (Mangrove Bay), 29 km south of the coastal city of El Qoseir; minimumDepthInMeters: 8.5; maximumDepthInMeters: 8.8; verbatimLatitude: N25°52.209′; verbatimLongitude: E034°25.181′; decimalLatitude: 25.87015; decimalLongitude: 34.4196833; geodeticDatum: WGS84; **Event:** samplingEffort: 3 x 20 m Line Intercept Transects; eventDate: 22-10-2023; year: 2023; habitat: current-exposed deeper reef slope; **Record Level:** institutionCode: UNIVIE; collectionCode: Coral_Reef-Mangrove_Bay-Egypt-2023; basisOfRecord: HumanObservation

##### Conservation status

Near Threatened

##### Notes

Fig. 39 .

#### Dipsastraea matthaii

(Vaughan, 1918)

8BA80528-EC78-585C-9AAF-CC3BEEFB6C98

##### Materials

- **Type status:** Other material. **Occurrence:** occurrenceRemarks: Observed and photographed on a current-exposed reef edge; occurrenceStatus: present; occurrenceID: 3E7198E9-9B1E-508F-9FF1-8E1247FFF659; **Taxon:** scientificNameID: urn:lsid:marinespecies.org:taxname:758240; scientificName: *Dipsastraeamatthaii*; kingdom: Animalia; phylum: Cnidaria; class: Anthozoa; taxonRank: Species; **Location:** continent: Africa; waterBody: Red Sea; country: Egypt; stateProvince: Muhafazah al Bahr al Ahmar; locality: Current-exposed coral reef located on the coastline of Egypt (Mangrove Bay), 29 km south of the coastal city of El Qoseir; verbatimDepth: 1; verbatimLatitude: N25°52.209′; verbatimLongitude: E034°25.181′; decimalLatitude: 25.87015; decimalLongitude: 34.4196833; geodeticDatum: WGS84; **Event:** samplingEffort: 3 x 20 m Line Intercept Transects; eventDate: 22-10-2023; year: 2023; habitat: current-exposed reef edge; **Record Level:** institutionCode: UNIVIE; collectionCode: Coral_Reef-Mangrove_Bay-Egypt-2023; basisOfRecord: HumanObservation
- **Type status:** Other material. **Occurrence:** occurrenceRemarks: Observed and photographed on a current-exposed deeper reef slope; occurrenceStatus: present; occurrenceID: A144BC07-D7F1-56BD-A78B-19FE4D8ECBDC; **Taxon:** scientificNameID: urn:lsid:marinespecies.org:taxname:758240; scientificName: *Dipsastraeamatthaii*; kingdom: Animalia; phylum: Cnidaria; class: Anthozoa; taxonRank: Species; **Location:** continent: Africa; waterBody: Red Sea; country: Egypt; stateProvince: Muhafazah al Bahr al Ahmar; locality: Current-exposed coral reef located on the coastline of Egypt (Mangrove Bay), 29 km south of the coastal city of El Qoseir; minimumDepthInMeters: 8.5; maximumDepthInMeters: 8.8; verbatimLatitude: N25°52.209′; verbatimLongitude: E034°25.181′; decimalLatitude: 25.87015; decimalLongitude: 34.4196833; geodeticDatum: WGS84; **Event:** samplingEffort: 3 x 20 m Line Intercept Transects; eventDate: 24-10-2023; year: 2023; habitat: current-exposed deeper reef slope; **Record Level:** institutionCode: UNIVIE; collectionCode: Coral_Reef-Mangrove_Bay-Egypt-2023; basisOfRecord: HumanObservation

##### Conservation status

Near Threatened

##### Notes

Fig. 40 .

#### Dipsastraea pallida

(Dana, 1846)

DE5A854B-7DA6-5E65-8872-03A6E5B5FEA4

##### Materials

- **Type status:** Other material. **Occurrence:** occurrenceRemarks: Observed and photographed on a current-exposed reef edge; occurrenceStatus: present; occurrenceID: 80FB4C4F-85E1-50E4-B547-F6CB34999898; **Taxon:** scientificNameID: urn:lsid:marinespecies.org:taxname:758233; scientificName: *Dipsastraeapallida*; kingdom: Animalia; phylum: Cnidaria; class: Anthozoa; taxonRank: Species; **Location:** continent: Africa; waterBody: Red Sea; country: Egypt; stateProvince: Muhafazah al Bahr al Ahmar; locality: Current-exposed coral reef located on the coastline of Egypt (Mangrove Bay), 29 km south of the coastal city of El Qoseir; verbatimDepth: 1; verbatimLatitude: N25°52.209′; verbatimLongitude: E034°25.181′; decimalLatitude: 25.87015; decimalLongitude: 34.4196833; geodeticDatum: WGS84; **Event:** samplingEffort: 3 x 20 m Line Intercept Transects; eventDate: 23-10-2023; year: 2023; habitat: current-exposed reef edge; **Record Level:** institutionCode: UNIVIE; collectionCode: Coral_Reef-Mangrove_Bay-Egypt-2023; basisOfRecord: HumanObservation
- **Type status:** Other material. **Occurrence:** occurrenceRemarks: Observed and photographed on a current-exposed shallow reef slope; occurrenceStatus: present; occurrenceID: 100477B5-2568-534F-B2B2-C61F21AD7F84; **Taxon:** scientificNameID: urn:lsid:marinespecies.org:taxname:758233; scientificName: *Dipsastraeapallida*; kingdom: Animalia; phylum: Cnidaria; class: Anthozoa; taxonRank: Species; **Location:** continent: Africa; waterBody: Red Sea; country: Egypt; stateProvince: Muhafazah al Bahr al Ahmar; locality: Current-exposed coral reef located on the coastline of Egypt (Mangrove Bay), 29 km south of the coastal city of El Qoseir; verbatimDepth: 4.3; verbatimLatitude: N25°52.209′; verbatimLongitude: E034°25.181′; decimalLatitude: 25.87015; decimalLongitude: 34.4196833; geodeticDatum: WGS84; **Event:** samplingEffort: 3 x 20 m Line Intercept Transects; eventDate: 24-10-2023; year: 2023; habitat: current-exposed shallow reef slope; **Record Level:** institutionCode: UNIVIE; collectionCode: Coral_Reef-Mangrove_Bay-Egypt-2023; basisOfRecord: HumanObservation
- **Type status:** Other material. **Occurrence:** occurrenceRemarks: Observed and photographed on a current-exposed deeper reef slope; occurrenceStatus: present; occurrenceID: 2C45F3FF-FE71-5650-8D42-3FDA6A05B513; **Taxon:** scientificNameID: urn:lsid:marinespecies.org:taxname:758233; scientificName: *Dipsastraeapallida*; kingdom: Animalia; phylum: Cnidaria; class: Anthozoa; taxonRank: Species; **Location:** continent: Africa; waterBody: Red Sea; country: Egypt; stateProvince: Muhafazah al Bahr al Ahmar; locality: Current-exposed coral reef located on the coastline of Egypt (Mangrove Bay), 29 km south of the coastal city of El Qoseir; minimumDepthInMeters: 8.5; maximumDepthInMeters: 8.8; verbatimLatitude: N25°52.209′; verbatimLongitude: E034°25.181′; decimalLatitude: 25.87015; decimalLongitude: 34.4196833; geodeticDatum: WGS84; **Event:** samplingEffort: 3 x 20 m Line Intercept Transects; eventDate: 22-10-2023; year: 2023; habitat: current-exposed deeper reef slope; **Record Level:** institutionCode: UNIVIE; collectionCode: Coral_Reef-Mangrove_Bay-Egypt-2023; basisOfRecord: HumanObservation

##### Conservation status

Least Concern

##### Notes

Fig. 41 .

#### Dipsastraea spp.

Blainville, 1830

7B6540CB-205F-51D1-A70C-2AE2EBB77EC2

##### Materials

- **Type status:** Other material. **Occurrence:** occurrenceRemarks: Observed on a current-exposed shallow reef slope; occurrenceID: DA74606A-C985-5161-A49C-37CADFF8321D; **Taxon:** scientificNameID: urn:lsid:marinespecies.org:taxname:718746; scientificName: *Dipsastraea* spp.; kingdom: Animalia; phylum: Cnidaria; class: Anthozoa; taxonRank: Genus; **Location:** continent: Africa; waterBody: Red Sea; country: Egypt; stateProvince: Muhafazah al Bahr al Ahmar; locality: Current-exposed coral reef located on the coastline of Egypt (Mangrove Bay), 29 km south of the coastal city of El Qoseir; verbatimDepth: 4.3; verbatimLatitude: N25°52.209′; verbatimLongitude: E034°25.181′; decimalLatitude: 25.87015; decimalLongitude: 34.4196833; geodeticDatum: WGS84; **Event:** samplingEffort: 3 x 20 m Line Intercept Transects; eventDate: 22-10-2023; year: 2023; habitat: current-exposed shallow reef slope; **Record Level:** institutionCode: UNIVIE; collectionCode: Coral_Reef-Mangrove_Bay-Egypt-2023; basisOfRecord: HumanObservation

##### Conservation status

Unknown

#### Dipsastraea speciosa

(Dana, 1846)

CD25FF3A-98EF-558B-BAC4-B94ACEFCDE49

##### Materials

- **Type status:** Other material. **Occurrence:** occurrenceRemarks: Observed and photographed on a current-exposed shallow reef slope; occurrenceStatus: present; occurrenceID: FC8191C9-0DB2-5DE8-9E8C-05CF5EBEC853; **Taxon:** scientificNameID: urn:lsid:marinespecies.org:taxname:758219; scientificName: *Dipsastraeaspeciosa*; kingdom: Animalia; phylum: Cnidaria; class: Anthozoa; taxonRank: Species; **Location:** continent: Africa; waterBody: Red Sea; country: Egypt; stateProvince: Muhafazah al Bahr al Ahmar; locality: Current-exposed coral reef located on the coastline of Egypt (Mangrove Bay), 29 km south of the coastal city of El Qoseir; verbatimDepth: 4.3; verbatimLatitude: N25°52.209′; verbatimLongitude: E034°25.181′; decimalLatitude: 25.87015; decimalLongitude: 34.4196833; geodeticDatum: WGS84; **Event:** samplingEffort: 3 x 20 m Line Intercept Transects; eventDate: 22-10-2023; year: 2023; habitat: current-exposed shallow reef slope; **Record Level:** institutionCode: UNIVIE; collectionCode: Coral_Reef-Mangrove_Bay-Egypt-2023; basisOfRecord: HumanObservation
- **Type status:** Other material. **Occurrence:** occurrenceRemarks: Observed and photographed on a current-exposed deeper reef slope; occurrenceStatus: present; occurrenceID: 69850653-3990-5979-950B-565031C4958B; **Taxon:** scientificNameID: urn:lsid:marinespecies.org:taxname:758219; scientificName: *Dipsastraeaspeciosa*; kingdom: Animalia; phylum: Cnidaria; class: Anthozoa; taxonRank: Species; **Location:** continent: Africa; waterBody: Red Sea; country: Egypt; stateProvince: Muhafazah al Bahr al Ahmar; locality: Current-exposed coral reef located on the coastline of Egypt (Mangrove Bay), 29 km south of the coastal city of El Qoseir; minimumDepthInMeters: 8.5; maximumDepthInMeters: 8.8; verbatimLatitude: N25°52.209′; verbatimLongitude: E034°25.181′; decimalLatitude: 25.87015; decimalLongitude: 34.4196833; geodeticDatum: WGS84; **Event:** samplingEffort: 3 x 20 m Line Intercept Transects; eventDate: 22-10-2023; year: 2023; habitat: current-exposed deeper reef slope; **Record Level:** institutionCode: UNIVIE; collectionCode: Coral_Reef-Mangrove_Bay-Egypt-2023; basisOfRecord: HumanObservation

##### Conservation status

Least Concern

##### Notes

Fig. 42 .

#### Echinopora forskaliana

(Milne Edwards & Haime, 1849)

DF2934D8-40D5-52B7-B551-D515487A944C

##### Materials

- **Type status:** Other material. **Occurrence:** occurrenceRemarks: Observed and photographed on a current-exposed shallow reef slope; occurrenceStatus: present; occurrenceID: F9047082-034A-5FDC-BC9A-F99419386507; **Taxon:** scientificNameID: urn:lsid:marinespecies.org:taxname:288341; scientificName: *Echinoporaforskaliana*; kingdom: Animalia; phylum: Cnidaria; class: Anthozoa; taxonRank: Species; **Location:** continent: Africa; waterBody: Red Sea; country: Egypt; stateProvince: Muhafazah al Bahr al Ahmar; locality: Current-exposed coral reef located on the coastline of Egypt (Mangrove Bay), 29 km south of the coastal city of El Qoseir; verbatimDepth: 4.3; verbatimLatitude: N25°52.209′; verbatimLongitude: E034°25.181′; decimalLatitude: 25.87015; decimalLongitude: 34.4196833; geodeticDatum: WGS84; **Event:** samplingEffort: 3 x 20 m Line Intercept Transects; eventDate: 24-10-2023; year: 2023; habitat: current-exposed shallow reef slope; **Record Level:** institutionCode: UNIVIE; collectionCode: Coral_Reef-Mangrove_Bay-Egypt-2023; basisOfRecord: HumanObservation
- **Type status:** Other material. **Occurrence:** occurrenceRemarks: Observed and photographed on a current-exposed deeper reef slope; occurrenceStatus: present; occurrenceID: 2EB0AA27-B4EE-53D8-870A-53B962438719; **Taxon:** scientificNameID: urn:lsid:marinespecies.org:taxname:288341; scientificName: *Echinoporaforskaliana*; kingdom: Animalia; phylum: Cnidaria; class: Anthozoa; taxonRank: Species; **Location:** continent: Africa; waterBody: Red Sea; country: Egypt; stateProvince: Muhafazah al Bahr al Ahmar; locality: Current-exposed coral reef located on the coastline of Egypt (Mangrove Bay), 29 km south of the coastal city of El Qoseir; minimumDepthInMeters: 8.5; maximumDepthInMeters: 8.8; verbatimLatitude: N25°52.209′; verbatimLongitude: E034°25.181′; decimalLatitude: 25.87015; decimalLongitude: 34.4196833; geodeticDatum: WGS84; **Event:** samplingEffort: 3 x 20 m Line Intercept Transects; eventDate: 22-10-2023; year: 2023; habitat: current-exposed deeper reef slope; **Record Level:** institutionCode: UNIVIE; collectionCode: Coral_Reef-Mangrove_Bay-Egypt-2023; basisOfRecord: HumanObservation

##### Conservation status

Near Threatened

##### Notes

Fig. 43 .

#### Echinopora fruticulosa

(Ehrenberg, 1834)

0A432A80-3914-5BD9-8B5B-B5AE8B8A7D55

##### Materials

- **Type status:** Other material. **Occurrence:** occurrenceRemarks: Observed and photographed on a current-exposed deeper reef slope; occurrenceStatus: present; occurrenceID: 7DE2740C-DB21-56E8-B215-3434677945C5; **Taxon:** scientificNameID: urn:lsid:marinespecies.org:taxname:207422; scientificName: *Echinoporafruticulosa*; kingdom: Animalia; phylum: Cnidaria; class: Anthozoa; taxonRank: Species; **Location:** continent: Africa; waterBody: Red Sea; country: Egypt; stateProvince: Muhafazah al Bahr al Ahmar; locality: Current-exposed coral reef located on the coastline of Egypt (Mangrove Bay), 29 km south of the coastal city of El Qoseir; minimumDepthInMeters: 8.5; maximumDepthInMeters: 8.8; verbatimLatitude: N25°52.209′; verbatimLongitude: E034°25.181′; decimalLatitude: 25.87015; decimalLongitude: 34.4196833; geodeticDatum: WGS84; **Event:** samplingEffort: 3 x 20 m Line Intercept Transects; eventDate: 24-10-2023; year: 2023; habitat: current-exposed deeper reef slope; **Record Level:** institutionCode: UNIVIE; collectionCode: Coral_Reef-Mangrove_Bay-Egypt-2023; basisOfRecord: HumanObservation
- **Type status:** Other material. **Occurrence:** occurrenceRemarks: Observed and photographed on a sheltered shallow reef slope; occurrenceStatus: present; occurrenceID: 287157AF-F388-5CBA-9945-EBF729B6379A; **Taxon:** scientificNameID: urn:lsid:marinespecies.org:taxname:207422; scientificName: *Echinoporafruticulosa*; kingdom: Animalia; phylum: Cnidaria; class: Anthozoa; taxonRank: Species; **Location:** continent: Africa; waterBody: Red Sea; country: Egypt; stateProvince: Muhafazah al Bahr al Ahmar; locality: Sheltered coral reef located on the coastline of Egypt (Mangrove Bay), 29 km south of the coastal city of El Qoseir; minimumDepthInMeters: 3.7; maximumDepthInMeters: 4.75; verbatimLatitude: N25°52.183′; verbatimLongitude: E034°25.140′; decimalLatitude: 25.8697167; decimalLongitude: 34.419; geodeticDatum: WGS84; **Event:** samplingEffort: 3 x 20 m Line Intercept Transects; eventDate: 25-10-2023; year: 2023; habitat: sheltered shallow reef slope; **Record Level:** institutionCode: UNIVIE; collectionCode: Coral_Reef-Mangrove_Bay-Egypt-2023; basisOfRecord: HumanObservation

##### Conservation status

Near Threatened

##### Notes

Fig. 44 .

#### Favites abdita

(Ellis & Solander, 1786)

D0A3DD93-C00A-5B14-8932-2FD49D6A995F

##### Materials

- **Type status:** Other material. **Occurrence:** occurrenceRemarks: Observed and photographed on a current-exposed reef edge; occurrenceStatus: present; occurrenceID: D4B73875-DF85-5757-BE5A-EC28F3C95518; **Taxon:** scientificNameID: urn:lsid:marinespecies.org:taxname:207449; scientificName: *Favitesabdita*; kingdom: Animalia; phylum: Cnidaria; class: Anthozoa; taxonRank: Species; **Location:** continent: Africa; waterBody: Red Sea; country: Egypt; stateProvince: Muhafazah al Bahr al Ahmar; locality: Current-exposed coral reef located on the coastline of Egypt (Mangrove Bay), 29 km south of the coastal city of El Qoseir; verbatimDepth: 1; verbatimLatitude: N25°52.209′; verbatimLongitude: E034°25.181′; decimalLatitude: 25.87015; decimalLongitude: 34.4196833; geodeticDatum: WGS84; **Event:** samplingEffort: 3 x 20 m Line Intercept Transects; eventDate: 24-10-2023; year: 2023; habitat: current-exposed reef edge; **Record Level:** institutionCode: UNIVIE; collectionCode: Coral_Reef-Mangrove_Bay-Egypt-2023; basisOfRecord: HumanObservation

##### Conservation status

Near Threatened

##### Notes

Fig. 45 .

#### Favites cf. complanata

(Ehrenberg, 1834)

A0753C64-9559-5D9B-9CC6-48BC63C59553

##### Materials

- **Type status:** Other material. **Occurrence:** occurrenceRemarks: Observed and photographed on a current-exposed deeper reef slope; occurrenceStatus: present; occurrenceID: 04DB58DE-34EA-5570-8691-059057D86722; **Taxon:** scientificNameID: urn:lsid:marinespecies.org:taxname:207455; scientificName: Favitescf.complanata; kingdom: Animalia; phylum: Cnidaria; class: Anthozoa; taxonRank: Species; **Location:** continent: Africa; waterBody: Red Sea; country: Egypt; stateProvince: Muhafazah al Bahr al Ahmar; locality: Current-exposed coral reef located on the coastline of Egypt (Mangrove Bay), 29 km south of the coastal city of El Qoseir; minimumDepthInMeters: 8.5; maximumDepthInMeters: 8.8; verbatimLatitude: N25°52.209′; verbatimLongitude: E034°25.181′; decimalLatitude: 25.87015; decimalLongitude: 34.4196833; geodeticDatum: WGS84; **Event:** samplingEffort: 3 x 20 m Line Intercept Transects; eventDate: 22-10-2023; year: 2023; habitat: current-exposed deeper reef slope; **Record Level:** institutionCode: UNIVIE; collectionCode: Coral_Reef-Mangrove_Bay-Egypt-2023; basisOfRecord: HumanObservation

##### Conservation status

Near Threatened

##### Notes

Fig. 46 .

#### Favites rotundata

Veron, Pichon & Wijsman-Best, 1977

C44C26C1-8027-5818-AD78-A0E22319700D

##### Materials

- **Type status:** Other material. **Occurrence:** occurrenceRemarks: Observed and photographed on a current-exposed deeper reef slope; occurrenceStatus: present; occurrenceID: 044870A2-EEE6-59D6-8AB5-DB3A747D07D7; **Taxon:** scientificNameID: urn:lsid:marinespecies.org:taxname:207445; scientificName: *Favitesrotundata*; kingdom: Animalia; phylum: Cnidaria; class: Anthozoa; taxonRank: Species; **Location:** continent: Africa; waterBody: Red Sea; country: Egypt; stateProvince: Muhafazah al Bahr al Ahmar; locality: Current-exposed coral reef located on the coastline of Egypt (Mangrove Bay), 29 km south of the coastal city of El Qoseir; minimumDepthInMeters: 8.5; maximumDepthInMeters: 8.8; verbatimLatitude: N25°52.209′; verbatimLongitude: E034°25.181′; decimalLatitude: 25.87015; decimalLongitude: 34.4196833; geodeticDatum: WGS84; **Event:** samplingEffort: 3 x 20 m Line Intercept Transects; eventDate: 22-10-2023; year: 2023; habitat: current-exposed deeper reef slope; **Record Level:** institutionCode: UNIVIE; collectionCode: Coral_Reef-Mangrove_Bay-Egypt-2023; basisOfRecord: HumanObservation

##### Conservation status

Near Threatened

##### Notes

Fig. 47 .

#### Favites vasta

(Klunzinger, 1879)

4470F417-E1E2-50BB-A620-7386F313568F

##### Materials

- **Type status:** Other material. **Occurrence:** occurrenceRemarks: Observed and photographed on a current-exposed shallow reef slope; occurrenceStatus: present; occurrenceID: 00C9DE5F-6F84-55D3-A716-63CFAB0D8359; **Taxon:** scientificNameID: urn:lsid:marinespecies.org:taxname:430663; scientificName: *Favitesvasta*; kingdom: Animalia; phylum: Cnidaria; class: Anthozoa; taxonRank: Species; **Location:** continent: Africa; waterBody: Red Sea; country: Egypt; stateProvince: Muhafazah al Bahr al Ahmar; locality: Current-exposed coral reef located on the coastline of Egypt (Mangrove Bay), 29 km south of the coastal city of El Qoseir; verbatimDepth: 4.3; verbatimLatitude: N25°52.209′; verbatimLongitude: E034°25.181′; decimalLatitude: 25.87015; decimalLongitude: 34.4196833; geodeticDatum: WGS84; **Event:** samplingEffort: 3 x 20 m Line Intercept Transects; eventDate: 22-10-2023; year: 2023; habitat: current-exposed shallow reef slope; **Record Level:** institutionCode: UNIVIE; collectionCode: Coral_Reef-Mangrove_Bay-Egypt-2023; basisOfRecord: HumanObservation

##### Conservation status

Near Threatened

##### Notes

Fig. 48 .

#### Goniastrea edwardsi

Chevalier, 1971

EEF72327-B1B4-528A-B3CB-414D9815E811

##### Materials

- **Type status:** Other material. **Occurrence:** occurrenceRemarks: Observed and photographed on a current-exposed reef edge; occurrenceStatus: present; occurrenceID: 72F1393E-E106-58BC-99F4-4DC70F46F70F; **Taxon:** scientificNameID: urn:lsid:marinespecies.org:taxname:207466; scientificName: *Goniastreaedwardsi*; kingdom: Animalia; phylum: Cnidaria; class: Anthozoa; taxonRank: Species; **Location:** continent: Africa; waterBody: Red Sea; country: Egypt; stateProvince: Muhafazah al Bahr al Ahmar; locality: Current-exposed coral reef located on the coastline of Egypt (Mangrove Bay), 29 km south of the coastal city of El Qoseir; verbatimDepth: 1; verbatimLatitude: N25°52.209′; verbatimLongitude: E034°25.181′; decimalLatitude: 25.87015; decimalLongitude: 34.4196833; geodeticDatum: WGS84; **Event:** samplingEffort: 3 x 20 m Line Intercept Transects; eventDate: 22-10-2023; year: 2023; habitat: current-exposed reef edge; **Record Level:** institutionCode: UNIVIE; collectionCode: Coral_Reef-Mangrove_Bay-Egypt-2023; basisOfRecord: HumanObservation
- **Type status:** Other material. **Occurrence:** occurrenceRemarks: Observed and photographed on a current-exposed shallow reef slope; occurrenceStatus: present; occurrenceID: D329FCAF-DCC3-5148-BD7E-15EDAFEED75D; **Taxon:** scientificNameID: urn:lsid:marinespecies.org:taxname:207466; scientificName: *Goniastreaedwardsi*; kingdom: Animalia; phylum: Cnidaria; class: Anthozoa; taxonRank: Species; **Location:** continent: Africa; waterBody: Red Sea; country: Egypt; stateProvince: Muhafazah al Bahr al Ahmar; locality: Current-exposed coral reef located on the coastline of Egypt (Mangrove Bay), 29 km south of the coastal city of El Qoseir; verbatimDepth: 4.3; verbatimLatitude: N25°52.209′; verbatimLongitude: E034°25.181′; decimalLatitude: 25.87015; decimalLongitude: 34.4196833; geodeticDatum: WGS84; **Event:** samplingEffort: 3 x 20 m Line Intercept Transects; eventDate: 22-10-2023; year: 2023; habitat: current-exposed shallow reef slope; **Record Level:** institutionCode: UNIVIE; collectionCode: Coral_Reef-Mangrove_Bay-Egypt-2023; basisOfRecord: HumanObservation
- **Type status:** Other material. **Occurrence:** occurrenceRemarks: Observed and photographed on a current-exposed deeper reef slope; occurrenceStatus: present; occurrenceID: 2BA7F3AD-9A89-58E0-B6C4-65E96BE7DE3D; **Taxon:** scientificNameID: urn:lsid:marinespecies.org:taxname:207466; scientificName: *Goniastreaedwardsi*; kingdom: Animalia; phylum: Cnidaria; class: Anthozoa; taxonRank: Species; **Location:** continent: Africa; waterBody: Red Sea; country: Egypt; stateProvince: Muhafazah al Bahr al Ahmar; locality: Current-exposed coral reef located on the coastline of Egypt (Mangrove Bay), 29 km south of the coastal city of El Qoseir; minimumDepthInMeters: 8.5; maximumDepthInMeters: 8.8; verbatimLatitude: N25°52.209′; verbatimLongitude: E034°25.181′; decimalLatitude: 25.87015; decimalLongitude: 34.4196833; geodeticDatum: WGS84; **Event:** samplingEffort: 3 x 20 m Line Intercept Transects; eventDate: 22-10-2023; year: 2023; habitat: current-exposed deeper reef slope; **Record Level:** institutionCode: UNIVIE; collectionCode: Coral_Reef-Mangrove_Bay-Egypt-2023; basisOfRecord: HumanObservation
- **Type status:** Other material. **Occurrence:** occurrenceRemarks: Observed and photographed on a sheltered reef edge; occurrenceStatus: present; occurrenceID: 67AC9D87-BEC6-56B0-898E-39D474E2C538; **Taxon:** scientificNameID: urn:lsid:marinespecies.org:taxname:207466; scientificName: *Goniastreaedwardsi*; kingdom: Animalia; phylum: Cnidaria; class: Anthozoa; taxonRank: Species; **Location:** continent: Africa; waterBody: Red Sea; country: Egypt; stateProvince: Muhafazah al Bahr al Ahmar; locality: Sheltered coral reef located on the coastline of Egypt (Mangrove Bay), 29 km south of the coastal city of El Qoseir; verbatimDepth: 1; verbatimLatitude: N25°52.183′; verbatimLongitude: E034°25.140′; decimalLatitude: 25.8697167; decimalLongitude: 34.419; geodeticDatum: WGS84; **Event:** samplingEffort: 3 x 20 m Line Intercept Transects; eventDate: 20-10-2023; year: 2023; habitat: sheltered reef edge; **Record Level:** institutionCode: UNIVIE; collectionCode: Coral_Reef-Mangrove_Bay-Egypt-2023; basisOfRecord: HumanObservation
- **Type status:** Other material. **Occurrence:** occurrenceRemarks: Observed and photographed on a sheltered shallow reef slope; occurrenceStatus: present; occurrenceID: DA4EA27B-5D3F-5AB9-BA35-A6DD039D9527; **Taxon:** scientificNameID: urn:lsid:marinespecies.org:taxname:207466; scientificName: *Goniastreaedwardsi*; kingdom: Animalia; phylum: Cnidaria; class: Anthozoa; taxonRank: Species; **Location:** continent: Africa; waterBody: Red Sea; country: Egypt; stateProvince: Muhafazah al Bahr al Ahmar; locality: Sheltered coral reef located on the coastline of Egypt (Mangrove Bay), 29 km south of the coastal city of El Qoseir; minimumDepthInMeters: 3.7; maximumDepthInMeters: 4.75; verbatimLatitude: N25°52.183′; verbatimLongitude: E034°25.140′; decimalLatitude: 25.8697167; decimalLongitude: 34.419; geodeticDatum: WGS84; **Event:** samplingEffort: 3 x 20 m Line Intercept Transects; eventDate: 25-10-2023; year: 2023; habitat: sheltered shallow reef slope; **Record Level:** institutionCode: UNIVIE; collectionCode: Coral_Reef-Mangrove_Bay-Egypt-2023; basisOfRecord: HumanObservation
- **Type status:** Other material. **Occurrence:** occurrenceRemarks: Observed and photographed on a sheltered deeper reef slope; occurrenceStatus: present; occurrenceID: FE40B90E-A9E7-563B-9CEB-8505B6719297; **Taxon:** scientificNameID: urn:lsid:marinespecies.org:taxname:207466; scientificName: *Goniastreaedwardsi*; kingdom: Animalia; phylum: Cnidaria; class: Anthozoa; taxonRank: Species; **Location:** continent: Africa; waterBody: Red Sea; country: Egypt; stateProvince: Muhafazah al Bahr al Ahmar; locality: Sheltered coral reef located on the coastline of Egypt (Mangrove Bay), 29 km south of the coastal city of El Qoseir; minimumDepthInMeters: 7.2; maximumDepthInMeters: 9.5; verbatimLatitude: N25°52.183′; verbatimLongitude: E034°25.140′; decimalLatitude: 25.8697167; decimalLongitude: 34.419; geodeticDatum: WGS84; **Event:** samplingEffort: 3 x 20 m Line Intercept Transects; eventDate: 23-10-2023; year: 2023; habitat: sheltered deeper reef slope; **Record Level:** institutionCode: UNIVIE; collectionCode: Coral_Reef-Mangrove_Bay-Egypt-2023; basisOfRecord: HumanObservation

##### Conservation status

Least Concern

##### Notes

Fig. 49 .

#### Goniastrea pectinata

(Ehrenberg, 1834)

E7E72BD8-A353-50FB-A5DF-BF44F837C366

##### Materials

- **Type status:** Other material. **Occurrence:** occurrenceRemarks: Observed and photographed on a current-exposed shallow reef slope; occurrenceStatus: present; occurrenceID: AC8951A8-66DA-508B-B487-8EB767413B9F; **Taxon:** scientificNameID: urn:lsid:marinespecies.org:taxname:207464; scientificName: *Goniastreapectinata*; kingdom: Animalia; phylum: Cnidaria; class: Anthozoa; taxonRank: Species; **Location:** continent: Africa; waterBody: Red Sea; country: Egypt; stateProvince: Muhafazah al Bahr al Ahmar; locality: Current-exposed coral reef located on the coastline of Egypt (Mangrove Bay), 29 km south of the coastal city of El Qoseir; verbatimDepth: 4.3; verbatimLatitude: N25°52.209′; verbatimLongitude: E034°25.181′; decimalLatitude: 25.87015; decimalLongitude: 34.4196833; geodeticDatum: WGS84; **Event:** samplingEffort: 3 x 20 m Line Intercept Transects; eventDate: 24-10-2023; year: 2023; habitat: current-exposed shallow reef slope; **Record Level:** institutionCode: UNIVIE; collectionCode: Coral_Reef-Mangrove_Bay-Egypt-2023; basisOfRecord: HumanObservation
- **Type status:** Other material. **Occurrence:** occurrenceRemarks: Observed and photographed on a current-exposed deeper reef slope; occurrenceStatus: present; occurrenceID: A1C3D673-B34A-583B-B502-B35075E8779B; **Taxon:** scientificNameID: urn:lsid:marinespecies.org:taxname:207464; scientificName: *Goniastreapectinata*; kingdom: Animalia; phylum: Cnidaria; class: Anthozoa; taxonRank: Species; **Location:** continent: Africa; waterBody: Red Sea; country: Egypt; stateProvince: Muhafazah al Bahr al Ahmar; locality: Current-exposed coral reef located on the coastline of Egypt (Mangrove Bay), 29 km south of the coastal city of El Qoseir; minimumDepthInMeters: 8.5; maximumDepthInMeters: 8.8; verbatimLatitude: N25°52.209′; verbatimLongitude: E034°25.181′; decimalLatitude: 25.87015; decimalLongitude: 34.4196833; geodeticDatum: WGS84; **Event:** samplingEffort: 3 x 20 m Line Intercept Transects; eventDate: 22-10-2023; year: 2023; habitat: current-exposed deeper reef slope; **Record Level:** institutionCode: UNIVIE; collectionCode: Coral_Reef-Mangrove_Bay-Egypt-2023; basisOfRecord: HumanObservation
- **Type status:** Other material. **Occurrence:** occurrenceRemarks: Observed and photographed on a sheltered deeper reef slope; occurrenceStatus: present; occurrenceID: B0F61796-8D9A-5D1F-80D7-90A2C64F1123; **Taxon:** scientificNameID: urn:lsid:marinespecies.org:taxname:207464; scientificName: *Goniastreapectinata*; kingdom: Animalia; phylum: Cnidaria; class: Anthozoa; taxonRank: Species; **Location:** continent: Africa; waterBody: Red Sea; country: Egypt; stateProvince: Muhafazah al Bahr al Ahmar; locality: Sheltered coral reef located on the coastline of Egypt (Mangrove Bay), 29 km south of the coastal city of El Qoseir; minimumDepthInMeters: 7.2; maximumDepthInMeters: 9.5; verbatimLatitude: N25°52.183′; verbatimLongitude: E034°25.140′; decimalLatitude: 25.8697167; decimalLongitude: 34.419; geodeticDatum: WGS84; **Event:** samplingEffort: 3 x 20 m Line Intercept Transects; eventDate: 23-10-2023; year: 2023; habitat: sheltered deeper reef slope; **Record Level:** institutionCode: UNIVIE; collectionCode: Coral_Reef-Mangrove_Bay-Egypt-2023; basisOfRecord: HumanObservation

##### Conservation status

Least Concern

##### Notes

Fig. 50 .

#### Goniastrea retiformis

(Lamarck, 1816)

A2F4C8E0-D6D3-53DE-8143-C85CA9E4AD68

##### Materials

- **Type status:** Other material. **Occurrence:** occurrenceRemarks: Observed and photographed on a current-exposed shallow reef slope; occurrenceStatus: present; occurrenceID: 7CE37B61-180F-5E13-98EE-170101124DE1; **Taxon:** scientificNameID: urn:lsid:marinespecies.org:taxname:207461; scientificName: *Goniastrearetiformis*; kingdom: Animalia; phylum: Cnidaria; class: Anthozoa; taxonRank: Species; **Location:** continent: Africa; waterBody: Red Sea; country: Egypt; stateProvince: Muhafazah al Bahr al Ahmar; locality: Current-exposed coral reef located on the coastline of Egypt (Mangrove Bay), 29 km south of the coastal city of El Qoseir; verbatimDepth: 4.3; verbatimLatitude: N25°52.209′; verbatimLongitude: E034°25.181′; decimalLatitude: 25.87015; decimalLongitude: 34.4196833; geodeticDatum: WGS84; **Event:** samplingEffort: 3 x 20 m Line Intercept Transects; eventDate: 22-10-2023; year: 2023; habitat: current-exposed shallow reef slope; **Record Level:** institutionCode: UNIVIE; collectionCode: Coral_Reef-Mangrove_Bay-Egypt-2023; basisOfRecord: HumanObservation

##### Conservation status

Least Concern

##### Notes

Fig. 51 .

#### Goniastrea sp.

Milne Edwards & Haime, 1848

841744C8-72C6-5A14-8437-6E17D78691C0

##### Materials

- **Type status:** Other material. **Occurrence:** occurrenceRemarks: Observed on a current-exposed shallow reef slope; occurrenceID: 259E8A80-3680-5613-8D94-7EFC4339BDDB; **Taxon:** scientificNameID: urn:lsid:marinespecies.org:taxname:205082; scientificName: *Goniastrea* sp.; kingdom: Animalia; phylum: Cnidaria; class: Anthozoa; taxonRank: Genus; **Location:** continent: Africa; waterBody: Red Sea; country: Egypt; stateProvince: Muhafazah al Bahr al Ahmar; locality: Current-exposed coral reef located on the coastline of Egypt (Mangrove Bay), 29 km south of the coastal city of El Qoseir; verbatimDepth: 4.3; verbatimLatitude: N25°52.209′; verbatimLongitude: E034°25.181′; decimalLatitude: 25.87015; decimalLongitude: 34.4196833; geodeticDatum: WGS84; **Event:** samplingEffort: 3 x 20 m Line Intercept Transects; eventDate: 24-10-2023; year: 2023; habitat: current-exposed shallow reef slope; **Record Level:** institutionCode: UNIVIE; collectionCode: Coral_Reef-Mangrove_Bay-Egypt-2023; basisOfRecord: HumanObservation

##### Conservation status

Unknown

#### Goniastrea stelligera

(Dana, 1846)

9B8D79DE-A399-556E-848B-8160E2801897

##### Materials

- **Type status:** Other material. **Occurrence:** occurrenceRemarks: Observed and photographed on a current-exposed reef edge; occurrenceStatus: present; occurrenceID: 4E5E0805-EFB1-57E1-AF4D-97C1E4801EB3; **Taxon:** scientificNameID: urn:lsid:marinespecies.org:taxname:763067; scientificName: *Goniastreastelligera*; kingdom: Animalia; phylum: Cnidaria; class: Anthozoa; taxonRank: Species; **Location:** continent: Africa; waterBody: Red Sea; country: Egypt; stateProvince: Muhafazah al Bahr al Ahmar; locality: Current-exposed coral reef located on the coastline of Egypt (Mangrove Bay), 29 km south of the coastal city of El Qoseir; verbatimDepth: 1; verbatimLatitude: N25°52.209′; verbatimLongitude: E034°25.181′; decimalLatitude: 25.87015; decimalLongitude: 34.4196833; geodeticDatum: WGS84; **Event:** samplingEffort: 3 x 20 m Line Intercept Transects; eventDate: 24-10-2023; year: 2023; habitat: current-exposed reef edge; **Record Level:** institutionCode: UNIVIE; collectionCode: Coral_Reef-Mangrove_Bay-Egypt-2023; basisOfRecord: HumanObservation

##### Conservation status

Near Threatened

##### Notes

Fig. 52 .

#### Merulina scheeri

Head, 1983

5404EA38-9670-52BF-BDD7-A771CE78B789

##### Materials

- **Type status:** Other material. **Occurrence:** occurrenceRemarks: Observed and photographed on a current-exposed deeper reef slope; occurrenceStatus: present; occurrenceID: 9AAA960D-0583-5AAA-9771-90F5F8BBAD1F; **Taxon:** scientificNameID: urn:lsid:marinespecies.org:taxname:207408; scientificName: *Merulinascheeri*; kingdom: Animalia; phylum: Cnidaria; class: Anthozoa; taxonRank: Species; **Location:** continent: Africa; waterBody: Red Sea; country: Egypt; stateProvince: Muhafazah al Bahr al Ahmar; locality: Current-exposed coral reef located on the coastline of Egypt (Mangrove Bay), 29 km south of the coastal city of El Qoseir; minimumDepthInMeters: 8.5; maximumDepthInMeters: 8.8; verbatimLatitude: N25°52.209′; verbatimLongitude: E034°25.181′; decimalLatitude: 25.87015; decimalLongitude: 34.4196833; geodeticDatum: WGS84; **Event:** samplingEffort: 3 x 20 m Line Intercept Transects; eventDate: 22-10-2023; year: 2023; habitat: current-exposed deeper reef slope; **Record Level:** institutionCode: UNIVIE; collectionCode: Coral_Reef-Mangrove_Bay-Egypt-2023; basisOfRecord: HumanObservation

##### Conservation status

Least Concern

##### Notes

Fig. 53 .

#### Paramontastraea peresi

(Faure & Pichon, 1978)

C644EF21-5E26-5174-8A37-5D0973988237

##### Materials

- **Type status:** Other material. **Occurrence:** occurrenceRemarks: Observed and photographed on a current-exposed deeper reef slope; occurrenceStatus: present; occurrenceID: 6ECF69A3-FCC8-524C-8849-A2CC0DE6EE28; **Taxon:** scientificNameID: urn:lsid:marinespecies.org:taxname:762398; scientificName: *Paramontastraeaperesi*; kingdom: Animalia; phylum: Cnidaria; class: Anthozoa; taxonRank: Species; **Location:** continent: Africa; waterBody: Red Sea; country: Egypt; stateProvince: Muhafazah al Bahr al Ahmar; locality: Current-exposed coral reef located on the coastline of Egypt (Mangrove Bay), 29 km south of the coastal city of El Qoseir; minimumDepthInMeters: 8.5; maximumDepthInMeters: 8.8; verbatimLatitude: N25°52.209′; verbatimLongitude: E034°25.181′; decimalLatitude: 25.87015; decimalLongitude: 34.4196833; geodeticDatum: WGS84; **Event:** samplingEffort: 3 x 20 m Line Intercept Transects; eventDate: 22-10-2023; year: 2023; habitat: current-exposed deeper reef slope; **Record Level:** institutionCode: UNIVIE; collectionCode: Coral_Reef-Mangrove_Bay-Egypt-2023; basisOfRecord: HumanObservation

##### Conservation status

Near Threatened

##### Notes

Fig. 54 .

#### Pachyseridae

Benzoni & Hoeksema, 2023

0CACCDDD-B1DA-5980-BC7A-8534BD917F27

#### Pachyseris speciosa

(Dana, 1846)

58163859-EE96-5E67-886B-DB49A12526A3

##### Materials

- **Type status:** Other material. **Occurrence:** occurrenceRemarks: Observed on a current-exposed shallow reef slope; occurrenceID: 3A768971-23E7-5C36-8967-953CEBE976C1; **Taxon:** scientificNameID: urn:lsid:marinespecies.org:taxname:207293; scientificName: *Pachyserisspeciosa*; kingdom: Animalia; phylum: Cnidaria; class: Anthozoa; taxonRank: Species; **Location:** continent: Africa; waterBody: Red Sea; country: Egypt; stateProvince: Muhafazah al Bahr al Ahmar; locality: Current-exposed coral reef located on the coastline of Egypt (Mangrove Bay), 29 km south of the coastal city of El Qoseir; verbatimDepth: 4.3; verbatimLatitude: N25°52.209′; verbatimLongitude: E034°25.181′; decimalLatitude: 25.87015; decimalLongitude: 34.4196833; geodeticDatum: WGS84; **Event:** samplingEffort: 3 x 20 m Line Intercept Transects; eventDate: 22-10-2023; year: 2023; habitat: current-exposed shallow reef slope; **Record Level:** institutionCode: UNIVIE; collectionCode: Coral_Reef-Mangrove_Bay-Egypt-2023; basisOfRecord: HumanObservation

##### Conservation status

Least Concern

#### Pocilloporidae

Gray, 1840

13A4529C-C19B-5C21-899B-C54F14D05196

#### Pocillopora damicornis

(Linnaeus, 1758)

60BD726F-7C92-5C88-ABE9-B3CDB5DDA828

##### Materials

- **Type status:** Other material. **Occurrence:** occurrenceRemarks: Observed and photographed on a current-exposed shallow reef slope; occurrenceStatus: present; occurrenceID: 8944B391-0D83-5712-B799-E993F4C395CB; **Taxon:** scientificNameID: urn:lsid:marinespecies.org:taxname:206953; scientificName: *Pocilloporadamicornis*; kingdom: Animalia; phylum: Cnidaria; class: Anthozoa; taxonRank: Species; **Location:** continent: Africa; waterBody: Red Sea; country: Egypt; stateProvince: Muhafazah al Bahr al Ahmar; locality: Current-exposed coral reef located on the coastline of Egypt (Mangrove Bay), 29 km south of the coastal city of El Qoseir; verbatimDepth: 4.3; verbatimLatitude: N25°52.209′; verbatimLongitude: E034°25.181′; decimalLatitude: 25.87015; decimalLongitude: 34.4196833; geodeticDatum: WGS84; **Event:** samplingEffort: 3 x 20 m Line Intercept Transects; eventDate: 22-10-2023; year: 2023; habitat: current-exposed shallow reef slope; **Record Level:** institutionCode: UNIVIE; collectionCode: Coral_Reef-Mangrove_Bay-Egypt-2023; basisOfRecord: HumanObservation
- **Type status:** Other material. **Occurrence:** occurrenceRemarks: Observed and photographed on a current-exposed deeper reef slope; occurrenceStatus: present; occurrenceID: 273F8FFD-FC8E-5812-A673-94C97C178EFF; **Taxon:** scientificNameID: urn:lsid:marinespecies.org:taxname:206953; scientificName: *Pocilloporadamicornis*; kingdom: Animalia; phylum: Cnidaria; class: Anthozoa; taxonRank: Species; **Location:** continent: Africa; waterBody: Red Sea; country: Egypt; stateProvince: Muhafazah al Bahr al Ahmar; locality: Current-exposed coral reef located on the coastline of Egypt (Mangrove Bay), 29 km south of the coastal city of El Qoseir; minimumDepthInMeters: 8.5; maximumDepthInMeters: 8.8; verbatimLatitude: N25°52.209′; verbatimLongitude: E034°25.181′; decimalLatitude: 25.87015; decimalLongitude: 34.4196833; geodeticDatum: WGS84; **Event:** samplingEffort: 3 x 20 m Line Intercept Transects; eventDate: 22-10-2023; year: 2023; habitat: current-exposed deeper reef slope; **Record Level:** institutionCode: UNIVIE; collectionCode: Coral_Reef-Mangrove_Bay-Egypt-2023; basisOfRecord: HumanObservation
- **Type status:** Other material. **Occurrence:** occurrenceRemarks: Observed and photographed on a sheltered reef edge; occurrenceStatus: present; occurrenceID: 8B4CF67F-4F1B-5EC6-94CA-E0105534355B; **Taxon:** scientificNameID: urn:lsid:marinespecies.org:taxname:206953; scientificName: *Pocilloporadamicornis*; kingdom: Animalia; phylum: Cnidaria; class: Anthozoa; taxonRank: Species; **Location:** continent: Africa; waterBody: Red Sea; country: Egypt; stateProvince: Muhafazah al Bahr al Ahmar; locality: Sheltered coral reef located on the coastline of Egypt (Mangrove Bay), 29 km south of the coastal city of El Qoseir; verbatimDepth: 1; verbatimLatitude: N25°52.183′; verbatimLongitude: E034°25.140′; decimalLatitude: 25.8697167; decimalLongitude: 34.419; geodeticDatum: WGS84; **Event:** samplingEffort: 3 x 20 m Line Intercept Transects; eventDate: 19-10-2023; year: 2023; habitat: sheltered reef edge; **Record Level:** institutionCode: UNIVIE; collectionCode: Coral_Reef-Mangrove_Bay-Egypt-2023; basisOfRecord: HumanObservation
- **Type status:** Other material. **Occurrence:** occurrenceRemarks: Observed and photographed on a sheltered shallow reef slope; occurrenceStatus: present; occurrenceID: 901CC9D9-D981-5A3E-A30C-DECEF3DE2D9C; **Taxon:** scientificNameID: urn:lsid:marinespecies.org:taxname:206953; scientificName: *Pocilloporadamicornis*; kingdom: Animalia; phylum: Cnidaria; class: Anthozoa; taxonRank: Species; **Location:** continent: Africa; waterBody: Red Sea; country: Egypt; stateProvince: Muhafazah al Bahr al Ahmar; locality: Sheltered coral reef located on the coastline of Egypt (Mangrove Bay), 29 km south of the coastal city of El Qoseir; minimumDepthInMeters: 3.7; maximumDepthInMeters: 4.75; verbatimLatitude: N25°52.183′; verbatimLongitude: E034°25.140′; decimalLatitude: 25.8697167; decimalLongitude: 34.419; geodeticDatum: WGS84; **Event:** samplingEffort: 3 x 20 m Line Intercept Transects; eventDate: 25-10-2023; year: 2023; habitat: sheltered shallow reef slope; **Record Level:** institutionCode: UNIVIE; collectionCode: Coral_Reef-Mangrove_Bay-Egypt-2023; basisOfRecord: HumanObservation

##### Conservation status

Least Concern

##### Notes

Fig. 55 .

#### Pocillopora spp.

Lamarck, 1816

6024244E-1C67-5DEF-842B-76062B6ADECA

##### Materials

- **Type status:** Other material. **Occurrence:** occurrenceRemarks: Observed and photographed on a current-exposed shallow reef slope; occurrenceStatus: present; occurrenceID: 2A1865D3-7947-50F8-AD24-4376BC19B8C9; **Taxon:** scientificNameID: urn:lsid:marinespecies.org:taxname:206938; scientificName: *Pocillopora* sp.; kingdom: Animalia; phylum: Cnidaria; class: Anthozoa; taxonRank: Genus; **Location:** continent: Africa; waterBody: Red Sea; country: Egypt; stateProvince: Muhafazah al Bahr al Ahmar; locality: Current-exposed coral reef located on the coastline of Egypt (Mangrove Bay), 29 km south of the coastal city of El Qoseir; verbatimDepth: 4.3; verbatimLatitude: N25°52.209′; verbatimLongitude: E034°25.181′; decimalLatitude: 25.87015; decimalLongitude: 34.4196833; geodeticDatum: WGS84; **Event:** samplingEffort: 3 x 20 m Line Intercept Transects; eventDate: 24-10-2023; year: 2023; habitat: current-exposed shallow reef slope; **Record Level:** institutionCode: UNIVIE; collectionCode: Coral_Reef-Mangrove_Bay-Egypt-2023; basisOfRecord: HumanObservation
- **Type status:** Other material. **Occurrence:** occurrenceRemarks: Observed and photographed on a current-exposed deeper reef slope; occurrenceStatus: present; occurrenceID: 54DC187B-C2A6-513B-A523-92FB9DB45903; **Taxon:** scientificNameID: urn:lsid:marinespecies.org:taxname:206938; scientificName: *Pocillopora* sp.; kingdom: Animalia; phylum: Cnidaria; class: Anthozoa; taxonRank: Genus; **Location:** continent: Africa; waterBody: Red Sea; country: Egypt; stateProvince: Muhafazah al Bahr al Ahmar; locality: Current-exposed coral reef located on the coastline of Egypt (Mangrove Bay), 29 km south of the coastal city of El Qoseir; minimumDepthInMeters: 8.5; maximumDepthInMeters: 8.8; verbatimLatitude: N25°52.209′; verbatimLongitude: E034°25.181′; decimalLatitude: 25.87015; decimalLongitude: 34.4196833; geodeticDatum: WGS84; **Event:** samplingEffort: 3 x 20 m Line Intercept Transects; eventDate: 22-10-2023; year: 2023; habitat: current-exposed deeper reef slope; **Record Level:** institutionCode: UNIVIE; collectionCode: Coral_Reef-Mangrove_Bay-Egypt-2023; basisOfRecord: HumanObservation
- **Type status:** Other material. **Occurrence:** occurrenceRemarks: Observed and photographed on a sheltered reef edge; occurrenceStatus: present; occurrenceID: 44BEB770-3F8C-5692-9340-D500D18BD8AD; **Taxon:** scientificNameID: urn:lsid:marinespecies.org:taxname:206938; scientificName: *Pocillopora* spp.; kingdom: Animalia; phylum: Cnidaria; class: Anthozoa; taxonRank: Genus; **Location:** continent: Africa; waterBody: Red Sea; country: Egypt; stateProvince: Muhafazah al Bahr al Ahmar; locality: Sheltered coral reef located on the coastline of Egypt (Mangrove Bay), 29 km south of the coastal city of El Qoseir; verbatimDepth: 1; verbatimLatitude: N25°52.183′; verbatimLongitude: E034°25.140′; decimalLatitude: 25.8697167; decimalLongitude: 34.419; geodeticDatum: WGS84; **Event:** samplingEffort: 3 x 20 m Line Intercept Transects; eventDate: 20-10-2023; year: 2023; habitat: sheltered reef edge; **Record Level:** institutionCode: UNIVIE; collectionCode: Coral_Reef-Mangrove_Bay-Egypt-2023; basisOfRecord: HumanObservation
- **Type status:** Other material. **Occurrence:** occurrenceRemarks: Observed and photographed on a sheltered shallow reef slope; occurrenceStatus: present; occurrenceID: DB92653B-5101-5E51-A81E-FBC97A29C659; **Taxon:** scientificNameID: urn:lsid:marinespecies.org:taxname:206938; scientificName: *Pocillopora* spp.; kingdom: Animalia; phylum: Cnidaria; class: Anthozoa; taxonRank: Genus; **Location:** continent: Africa; waterBody: Red Sea; country: Egypt; stateProvince: Muhafazah al Bahr al Ahmar; locality: Sheltered coral reef located on the coastline of Egypt (Mangrove Bay), 29 km south of the coastal city of El Qoseir; minimumDepthInMeters: 3.7; maximumDepthInMeters: 4.75; verbatimLatitude: N25°52.183′; verbatimLongitude: E034°25.140′; decimalLatitude: 25.8697167; decimalLongitude: 34.419; geodeticDatum: WGS84; **Event:** samplingEffort: 3 x 20 m Line Intercept Transects; eventDate: 23-10-2023; year: 2023; habitat: sheltered shallow reef slope; **Record Level:** institutionCode: UNIVIE; collectionCode: Coral_Reef-Mangrove_Bay-Egypt-2023; basisOfRecord: HumanObservation
- **Type status:** Other material. **Occurrence:** occurrenceRemarks: Observed and photographed on a sheltered deeper reef slope; occurrenceStatus: present; occurrenceID: 5C43708F-388A-5B6E-A154-30EF4C0F089B; **Taxon:** scientificNameID: urn:lsid:marinespecies.org:taxname:206938; scientificName: *Pocillopora* sp.; kingdom: Animalia; phylum: Cnidaria; class: Anthozoa; taxonRank: Genus; **Location:** continent: Africa; waterBody: Red Sea; country: Egypt; stateProvince: Muhafazah al Bahr al Ahmar; locality: Sheltered coral reef located on the coastline of Egypt (Mangrove Bay), 29 km south of the coastal city of El Qoseir; minimumDepthInMeters: 7.2; maximumDepthInMeters: 9.5; verbatimLatitude: N25°52.183′; verbatimLongitude: E034°25.140′; decimalLatitude: 25.8697167; decimalLongitude: 34.419; geodeticDatum: WGS84; **Event:** samplingEffort: 3 x 20 m Line Intercept Transects; eventDate: 23-10-2023; year: 2023; habitat: sheltered deeper reef slope; **Record Level:** institutionCode: UNIVIE; collectionCode: Coral_Reef-Mangrove_Bay-Egypt-2023; basisOfRecord: HumanObservation

##### Conservation status

Unknown

##### Notes

**Figure 56.**
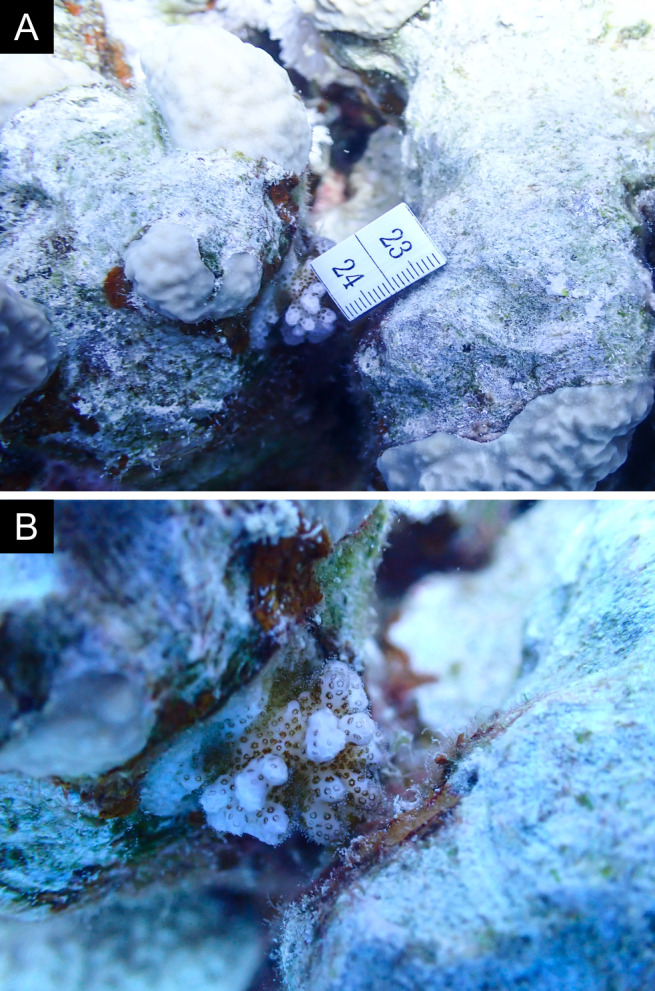
The growth form and appearance of a small *Pocillopora* sp. in the reef are shown in (**A**), while (**B**) provides a close-up of the colony. Photo credits: Lewis Alan Jones.

Fig. 56; one distinguished morphotype.

#### Pocillopora verrucosa

(Ellis & Solander, 1786)

7F2DB783-DF4D-5F32-8357-6AAC6BEE19F1

##### Materials

- **Type status:** Other material. **Occurrence:** occurrenceRemarks: Observed and photographed on a current-exposed reef edge; occurrenceStatus: present; occurrenceID: 8BD2833D-5FE9-56EA-8924-0C7F5F750448; **Taxon:** scientificNameID: urn:lsid:marinespecies.org:taxname:206954; scientificName: *Pocilloporaverrucosa*; kingdom: Animalia; phylum: Cnidaria; class: Anthozoa; taxonRank: Species; **Location:** continent: Africa; waterBody: Red Sea; country: Egypt; stateProvince: Muhafazah al Bahr al Ahmar; locality: Current-exposed coral reef located on the coastline of Egypt (Mangrove Bay), 29 km south of the coastal city of El Qoseir; verbatimDepth: 1; verbatimLatitude: N25°52.209′; verbatimLongitude: E034°25.181′; decimalLatitude: 25.87015; decimalLongitude: 34.4196833; geodeticDatum: WGS84; **Event:** samplingEffort: 3 x 20 m Line Intercept Transects; eventDate: 22-10-2023; year: 2023; habitat: current-exposed reef edge; **Record Level:** institutionCode: UNIVIE; collectionCode: Coral_Reef-Mangrove_Bay-Egypt-2023; basisOfRecord: HumanObservation
- **Type status:** Other material. **Occurrence:** occurrenceRemarks: Observed and photographed on a current-exposed shallow reef slope; occurrenceStatus: present; occurrenceID: 9021FBEF-A721-5117-A0C1-9800CD3C96E3; **Taxon:** scientificNameID: urn:lsid:marinespecies.org:taxname:206954; scientificName: *Pocilloporaverrucosa*; kingdom: Animalia; phylum: Cnidaria; class: Anthozoa; taxonRank: Species; **Location:** continent: Africa; waterBody: Red Sea; country: Egypt; stateProvince: Muhafazah al Bahr al Ahmar; locality: Current-exposed coral reef located on the coastline of Egypt (Mangrove Bay), 29 km south of the coastal city of El Qoseir; verbatimDepth: 4.3; verbatimLatitude: N25°52.209′; verbatimLongitude: E034°25.181′; decimalLatitude: 25.87015; decimalLongitude: 34.4196833; geodeticDatum: WGS84; **Event:** samplingEffort: 3 x 20 m Line Intercept Transects; eventDate: 22-10-2023; year: 2023; habitat: current-exposed shallow reef slope; **Record Level:** institutionCode: UNIVIE; collectionCode: Coral_Reef-Mangrove_Bay-Egypt-2023; basisOfRecord: HumanObservation
- **Type status:** Other material. **Occurrence:** occurrenceRemarks: Observed and photographed on a current-exposed deeper reef slope; occurrenceStatus: present; occurrenceID: 457DA6B4-B72A-54F7-811D-C15B2EE87333; **Taxon:** scientificNameID: urn:lsid:marinespecies.org:taxname:206954; scientificName: *Pocilloporaverrucosa*; kingdom: Animalia; phylum: Cnidaria; class: Anthozoa; taxonRank: Species; **Location:** continent: Africa; waterBody: Red Sea; country: Egypt; stateProvince: Muhafazah al Bahr al Ahmar; locality: Current-exposed coral reef located on the coastline of Egypt (Mangrove Bay), 29 km south of the coastal city of El Qoseir; minimumDepthInMeters: 8.5; maximumDepthInMeters: 8.8; verbatimLatitude: N25°52.209′; verbatimLongitude: E034°25.181′; decimalLatitude: 25.87015; decimalLongitude: 34.4196833; geodeticDatum: WGS84; **Event:** samplingEffort: 3 x 20 m Line Intercept Transects; eventDate: 22-10-2023; year: 2023; habitat: current-exposed deeper reef slope; **Record Level:** institutionCode: UNIVIE; collectionCode: Coral_Reef-Mangrove_Bay-Egypt-2023; basisOfRecord: HumanObservation
- **Type status:** Other material. **Occurrence:** occurrenceRemarks: Observed and photographed on a sheltered reef edge; occurrenceStatus: present; occurrenceID: C992AD78-EB12-5C04-B3BA-2E64DA7DBE1A; **Taxon:** scientificNameID: urn:lsid:marinespecies.org:taxname:206954; scientificName: *Pocilloporaverrucosa*; kingdom: Animalia; phylum: Cnidaria; class: Anthozoa; taxonRank: Species; **Location:** continent: Africa; waterBody: Red Sea; country: Egypt; stateProvince: Muhafazah al Bahr al Ahmar; locality: Sheltered coral reef located on the coastline of Egypt (Mangrove Bay), 29 km south of the coastal city of El Qoseir; verbatimDepth: 1; verbatimLatitude: N25°52.183′; verbatimLongitude: E034°25.140′; decimalLatitude: 25.8697167; decimalLongitude: 34.419; geodeticDatum: WGS84; **Event:** samplingEffort: 3 x 20 m Line Intercept Transects; eventDate: 19-10-2023; year: 2023; habitat: sheltered reef edge; **Record Level:** institutionCode: UNIVIE; collectionCode: Coral_Reef-Mangrove_Bay-Egypt-2023; basisOfRecord: HumanObservation
- **Type status:** Other material. **Occurrence:** occurrenceRemarks: Observed and photographed on a sheltered shallow reef slope; occurrenceStatus: present; occurrenceID: 6DF9B283-01B4-532C-81E2-DE5D77C8559A; **Taxon:** scientificNameID: urn:lsid:marinespecies.org:taxname:206954; scientificName: *Pocilloporaverrucosa*; kingdom: Animalia; phylum: Cnidaria; class: Anthozoa; taxonRank: Species; **Location:** continent: Africa; waterBody: Red Sea; country: Egypt; stateProvince: Muhafazah al Bahr al Ahmar; locality: Sheltered coral reef located on the coastline of Egypt (Mangrove Bay), 29 km south of the coastal city of El Qoseir; minimumDepthInMeters: 3.7; maximumDepthInMeters: 4.75; verbatimLatitude: N25°52.183′; verbatimLongitude: E034°25.140′; decimalLatitude: 25.8697167; decimalLongitude: 34.419; geodeticDatum: WGS84; **Event:** samplingEffort: 3 x 20 m Line Intercept Transects; eventDate: 23-10-2023; year: 2023; habitat: sheltered shallow reef slope; **Record Level:** institutionCode: UNIVIE; collectionCode: Coral_Reef-Mangrove_Bay-Egypt-2023; basisOfRecord: HumanObservation
- **Type status:** Other material. **Occurrence:** occurrenceRemarks: Observed and photographed on a sheltered deeper reef slope; occurrenceStatus: present; occurrenceID: E9E7737D-0034-5402-809C-91BDD70D54C3; **Taxon:** scientificNameID: urn:lsid:marinespecies.org:taxname:206954; scientificName: *Pocilloporaverrucosa*; kingdom: Animalia; phylum: Cnidaria; class: Anthozoa; taxonRank: Species; **Location:** continent: Africa; waterBody: Red Sea; country: Egypt; stateProvince: Muhafazah al Bahr al Ahmar; locality: Sheltered coral reef located on the coastline of Egypt (Mangrove Bay), 29 km south of the coastal city of El Qoseir; minimumDepthInMeters: 7.2; maximumDepthInMeters: 9.5; verbatimLatitude: N25°52.183′; verbatimLongitude: E034°25.140′; decimalLatitude: 25.8697167; decimalLongitude: 34.419; geodeticDatum: WGS84; **Event:** samplingEffort: 3 x 20 m Line Intercept Transects; eventDate: 23-10-2023; year: 2023; habitat: sheltered deeper reef slope; **Record Level:** institutionCode: UNIVIE; collectionCode: Coral_Reef-Mangrove_Bay-Egypt-2023; basisOfRecord: HumanObservation

##### Conservation status

Least Concern

##### Notes

Fig. 57 .

#### Seriatopora caliendrum

Ehrenberg, 1834

CEEFA91B-EC50-5909-83AC-C824532851E4

##### Materials

- **Type status:** Other material. **Occurrence:** occurrenceRemarks: Observed and photographed on a current-exposed shallow reef slope; occurrenceStatus: present; occurrenceID: 73D92B49-615A-5879-B4E3-8CEA672CDAD3; **Taxon:** scientificNameID: urn:lsid:marinespecies.org:taxname:206969; scientificName: *Seriatoporacaliendrum*; kingdom: Animalia; phylum: Cnidaria; class: Anthozoa; taxonRank: Species; **Location:** continent: Africa; waterBody: Red Sea; country: Egypt; stateProvince: Muhafazah al Bahr al Ahmar; locality: Current-exposed coral reef located on the coastline of Egypt (Mangrove Bay), 29 km south of the coastal city of El Qoseir; verbatimDepth: 4.3; verbatimLatitude: N25°52.209′; verbatimLongitude: E034°25.181′; decimalLatitude: 25.87015; decimalLongitude: 34.4196833; geodeticDatum: WGS84; **Event:** samplingEffort: 3 x 20 m Line Intercept Transects; eventDate: 24-10-2023; year: 2023; habitat: current-exposed shallow reef slope; **Record Level:** institutionCode: UNIVIE; collectionCode: Coral_Reef-Mangrove_Bay-Egypt-2023; basisOfRecord: HumanObservation
- **Type status:** Other material. **Occurrence:** occurrenceRemarks: Observed and photographed on a current-exposed deeper reef slope; occurrenceStatus: present; occurrenceID: 5CA8A815-65F4-5EE2-8FD1-2D7F647D9F1E; **Taxon:** scientificNameID: urn:lsid:marinespecies.org:taxname:206969; scientificName: *Seriatoporacaliendrum*; kingdom: Animalia; phylum: Cnidaria; class: Anthozoa; taxonRank: Species; **Location:** continent: Africa; waterBody: Red Sea; country: Egypt; stateProvince: Muhafazah al Bahr al Ahmar; locality: Current-exposed coral reef located on the coastline of Egypt (Mangrove Bay), 29 km south of the coastal city of El Qoseir; minimumDepthInMeters: 8.5; maximumDepthInMeters: 8.8; verbatimLatitude: N25°52.209′; verbatimLongitude: E034°25.181′; decimalLatitude: 25.87015; decimalLongitude: 34.4196833; geodeticDatum: WGS84; **Event:** samplingEffort: 3 x 20 m Line Intercept Transects; eventDate: 24-10-2023; year: 2023; habitat: current-exposed deeper reef slope; **Record Level:** institutionCode: UNIVIE; collectionCode: Coral_Reef-Mangrove_Bay-Egypt-2023; basisOfRecord: HumanObservation
- **Type status:** Other material. **Occurrence:** occurrenceRemarks: Observed and photographed on a sheltered deeper reef slope; occurrenceStatus: present; occurrenceID: 6655F17E-185C-5246-878F-3CB14913BF09; **Taxon:** scientificNameID: urn:lsid:marinespecies.org:taxname:206969; scientificName: *Seriatoporacaliendrum*; kingdom: Animalia; phylum: Cnidaria; class: Anthozoa; taxonRank: Species; **Location:** continent: Africa; waterBody: Red Sea; country: Egypt; stateProvince: Muhafazah al Bahr al Ahmar; locality: Sheltered coral reef located on the coastline of Egypt (Mangrove Bay), 29 km south of the coastal city of El Qoseir; minimumDepthInMeters: 7.2; maximumDepthInMeters: 9.5; verbatimLatitude: N25°52.183′; verbatimLongitude: E034°25.140′; decimalLatitude: 25.8697167; decimalLongitude: 34.419; geodeticDatum: WGS84; **Event:** samplingEffort: 3 x 20 m Line Intercept Transects; eventDate: 23-10-2023; year: 2023; habitat: sheltered deeper reef slope; **Record Level:** institutionCode: UNIVIE; collectionCode: Coral_Reef-Mangrove_Bay-Egypt-2023; basisOfRecord: HumanObservation

##### Conservation status

Near Threatened

##### Notes

Fig. 58 .

#### Seriatopora hystrix

Dana, 1846

484E0FB6-0B01-50D7-9C97-66BDC283BE4B

##### Materials

- **Type status:** Other material. **Occurrence:** occurrenceRemarks: Observed and photographed on a current-exposed shallow reef slope; occurrenceStatus: present; occurrenceID: FD87CA1E-191C-575F-B37F-E07E68738B02; **Taxon:** scientificNameID: urn:lsid:marinespecies.org:taxname:206973; scientificName: *Seriatoporahystrix*; kingdom: Animalia; phylum: Cnidaria; class: Anthozoa; taxonRank: Species; **Location:** continent: Africa; waterBody: Red Sea; country: Egypt; stateProvince: Muhafazah al Bahr al Ahmar; locality: Current-exposed coral reef located on the coastline of Egypt (Mangrove Bay), 29 km south of the coastal city of El Qoseir; verbatimDepth: 4.3; verbatimLatitude: N25°52.209′; verbatimLongitude: E034°25.181′; decimalLatitude: 25.87015; decimalLongitude: 34.4196833; geodeticDatum: WGS84; **Event:** samplingEffort: 3 x 20 m Line Intercept Transects; eventDate: 24-10-2023; year: 2023; habitat: current-exposed shallow reef slope; **Record Level:** institutionCode: UNIVIE; collectionCode: Coral_Reef-Mangrove_Bay-Egypt-2023; basisOfRecord: HumanObservation

##### Conservation status

Least Concern

##### Notes

Fig. 59 .

#### Stylophora cf. kuehlmanni

Scheer & Pillai, 1983

F91B937A-B0D5-536C-8CF2-8DC9019B3456

##### Materials

- **Type status:** Other material. **Occurrence:** occurrenceRemarks: Observed and photographed on a sheltered shallow reef slope; occurrenceStatus: present; occurrenceID: 74DB88D8-0130-5811-90C3-A935DA8AFABD; **Taxon:** scientificNameID: urn:lsid:marinespecies.org:taxname:206985; scientificName: Stylophoracf.kuehlmanni; kingdom: Animalia; phylum: Cnidaria; class: Anthozoa; taxonRank: Species; **Location:** continent: Africa; waterBody: Red Sea; country: Egypt; stateProvince: Muhafazah al Bahr al Ahmar; locality: Sheltered coral reef located on the coastline of Egypt (Mangrove Bay), 29 km south of the coastal city of El Qoseir; minimumDepthInMeters: 3.7; maximumDepthInMeters: 4.75; verbatimLatitude: N25°52.183′; verbatimLongitude: E034°25.140′; decimalLatitude: 25.8697167; decimalLongitude: 34.419; geodeticDatum: WGS84; **Event:** samplingEffort: 3 x 20 m Line Intercept Transects; eventDate: 25-10-2023; year: 2023; habitat: sheltered shallow reef slope; **Record Level:** institutionCode: UNIVIE; collectionCode: Coral_Reef-Mangrove_Bay-Egypt-2023; basisOfRecord: HumanObservation

##### Conservation status

Least Concern

##### Notes

**Figure 60.**
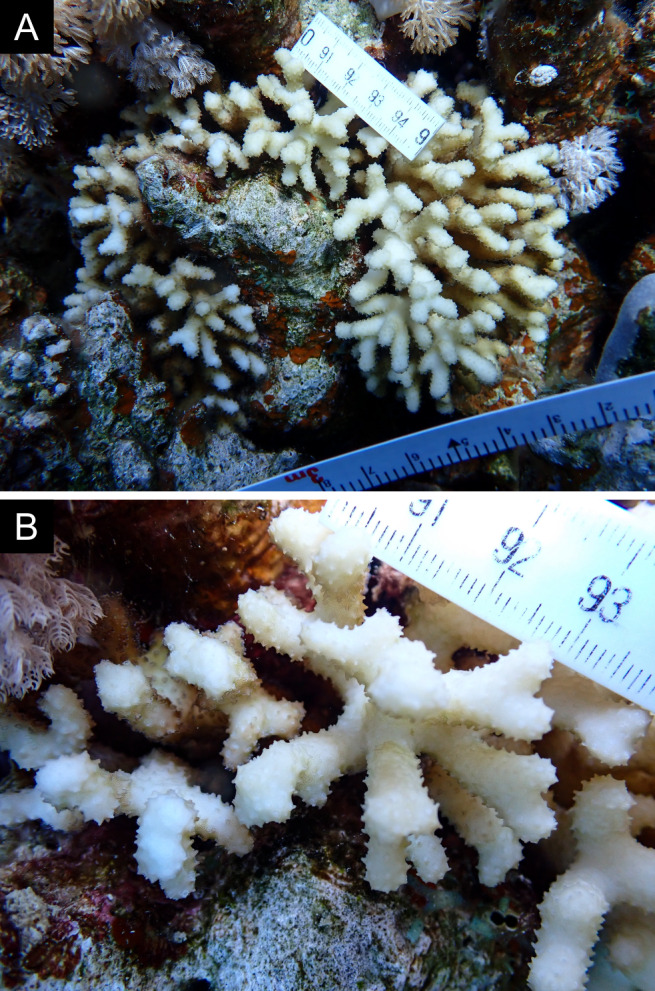
The growth form and appearance of Stylophoracf.kuehlmanni (bleached) in the reef are shown in (**A**), while (**B**) provides a close-up of the colony. Photo credits: Joseph Wallace Daurella.

Fig. 60; possible alternative: *Stylophorapistillata* (Esper, 1792).

#### Stylophora pistillata

(Esper, 1792)

771B005C-2E10-5D04-936E-C3383C0E85EB

##### Materials

- **Type status:** Other material. **Occurrence:** occurrenceRemarks: Observed and photographed on a current-exposed reef edge; occurrenceStatus: present; occurrenceID: 7626B972-1299-56B7-8B02-EEA1A13E7C09; **Taxon:** scientificNameID: urn:lsid:marinespecies.org:taxname:206982; scientificName: *Stylophorapistillata*; kingdom: Animalia; phylum: Cnidaria; class: Anthozoa; taxonRank: Species; **Location:** continent: Africa; waterBody: Red Sea; country: Egypt; stateProvince: Muhafazah al Bahr al Ahmar; locality: Current-exposed coral reef located on the coastline of Egypt (Mangrove Bay), 29 km south of the coastal city of El Qoseir; verbatimDepth: 1; verbatimLatitude: N25°52.209′; verbatimLongitude: E034°25.181′; decimalLatitude: 25.87015; decimalLongitude: 34.4196833; geodeticDatum: WGS84; **Event:** samplingEffort: 3 x 20 m Line Intercept Transects; eventDate: 24-10-2023; year: 2023; habitat: current-exposed reef edge; **Record Level:** institutionCode: UNIVIE; collectionCode: Coral_Reef-Mangrove_Bay-Egypt-2023; basisOfRecord: HumanObservation
- **Type status:** Other material. **Occurrence:** occurrenceRemarks: Observed and photographed on a current-exposed shallow reef slope; occurrenceStatus: present; occurrenceID: 04797C93-7863-5C08-B3A1-7782E17D8ECF; **Taxon:** scientificNameID: urn:lsid:marinespecies.org:taxname:206982; scientificName: *Stylophorapistillata*; kingdom: Animalia; phylum: Cnidaria; class: Anthozoa; taxonRank: Species; **Location:** continent: Africa; waterBody: Red Sea; country: Egypt; stateProvince: Muhafazah al Bahr al Ahmar; locality: Current-exposed coral reef located on the coastline of Egypt (Mangrove Bay), 29 km south of the coastal city of El Qoseir; verbatimDepth: 4.3; verbatimLatitude: N25°52.209′; verbatimLongitude: E034°25.181′; decimalLatitude: 25.87015; decimalLongitude: 34.4196833; geodeticDatum: WGS84; **Event:** samplingEffort: 3 x 20 m Line Intercept Transects; eventDate: 24-10-2023; year: 2023; habitat: current-exposed shallow reef slope; **Record Level:** institutionCode: UNIVIE; collectionCode: Coral_Reef-Mangrove_Bay-Egypt-2023; basisOfRecord: HumanObservation
- **Type status:** Other material. **Occurrence:** occurrenceRemarks: Observed and photographed on a sheltered shallow reef slope; occurrenceStatus: present; occurrenceID: FC495889-314F-5878-A039-6AAA81F9F1A7; **Taxon:** scientificNameID: urn:lsid:marinespecies.org:taxname:206982; scientificName: *Stylophorapistillata*; kingdom: Animalia; phylum: Cnidaria; class: Anthozoa; taxonRank: Species; **Location:** continent: Africa; waterBody: Red Sea; country: Egypt; stateProvince: Muhafazah al Bahr al Ahmar; locality: Sheltered coral reef located on the coastline of Egypt (Mangrove Bay), 29 km south of the coastal city of El Qoseir; minimumDepthInMeters: 3.7; maximumDepthInMeters: 4.75; verbatimLatitude: N25°52.183′; verbatimLongitude: E034°25.140′; decimalLatitude: 25.8697167; decimalLongitude: 34.419; geodeticDatum: WGS84; **Event:** samplingEffort: 3 x 20 m Line Intercept Transects; eventDate: 25-10-2023; year: 2023; habitat: sheltered shallow reef slope; **Record Level:** institutionCode: UNIVIE; collectionCode: Coral_Reef-Mangrove_Bay-Egypt-2023; basisOfRecord: HumanObservation
- **Type status:** Other material. **Occurrence:** occurrenceRemarks: Observed and photographed on a sheltered deeper reef slope; occurrenceStatus: present; occurrenceID: 1942CC36-5BBF-5263-93EA-7BE2A0AF84DF; **Taxon:** scientificNameID: urn:lsid:marinespecies.org:taxname:206982; scientificName: *Stylophorapistillata*; kingdom: Animalia; phylum: Cnidaria; class: Anthozoa; taxonRank: Species; **Location:** continent: Africa; waterBody: Red Sea; country: Egypt; stateProvince: Muhafazah al Bahr al Ahmar; locality: Sheltered coral reef located on the coastline of Egypt (Mangrove Bay), 29 km south of the coastal city of El Qoseir; minimumDepthInMeters: 7.2; maximumDepthInMeters: 9.5; verbatimLatitude: N25°52.183′; verbatimLongitude: E034°25.140′; decimalLatitude: 25.8697167; decimalLongitude: 34.419; geodeticDatum: WGS84; **Event:** samplingEffort: 3 x 20 m Line Intercept Transects; eventDate: 23-10-2023; year: 2023; habitat: sheltered deeper reef slope; **Record Level:** institutionCode: UNIVIE; collectionCode: Coral_Reef-Mangrove_Bay-Egypt-2023; basisOfRecord: HumanObservation

##### Conservation status

Near Threatened

##### Notes

Fig. 61 .

#### Stylophora spp.

Schweigger, 1820

8529F964-3E6A-5E1A-AEBA-F4A34D371CEA

##### Materials

- **Type status:** Other material. **Occurrence:** occurrenceRemarks: Observed and photographed on a sheltered shallow reef slope; occurrenceStatus: present; occurrenceID: 69AB4EB1-F811-559B-AEEE-C6FBCE3F29A0; **Taxon:** scientificNameID: urn:lsid:marinespecies.org:taxname:204068; scientificName: *Stylophora* spp.; kingdom: Animalia; phylum: Cnidaria; class: Anthozoa; taxonRank: Genus; **Location:** continent: Africa; waterBody: Red Sea; country: Egypt; stateProvince: Muhafazah al Bahr al Ahmar; locality: Sheltered coral reef located on the coastline of Egypt (Mangrove Bay), 29 km south of the coastal city of El Qoseir; minimumDepthInMeters: 3.7; maximumDepthInMeters: 4.75; verbatimLatitude: N25°52.183′; verbatimLongitude: E034°25.140′; decimalLatitude: 25.8697167; decimalLongitude: 34.419; geodeticDatum: WGS84; **Event:** samplingEffort: 3 x 20 m Line Intercept Transects; eventDate: 25-10-2023; year: 2023; habitat: sheltered shallow reef slope; **Record Level:** institutionCode: UNIVIE; collectionCode: Coral_Reef-Mangrove_Bay-Egypt-2023; basisOfRecord: HumanObservation
- **Type status:** Other material. **Occurrence:** occurrenceRemarks: Observed and photographed on a sheltered deeper reef slope; occurrenceStatus: present; occurrenceID: 6A348F78-CE51-546F-A539-B976FCCBE39E; **Taxon:** scientificNameID: urn:lsid:marinespecies.org:taxname:204068; scientificName: *Stylophora* sp.; kingdom: Animalia; phylum: Cnidaria; class: Anthozoa; taxonRank: Genus; **Location:** continent: Africa; waterBody: Red Sea; country: Egypt; stateProvince: Muhafazah al Bahr al Ahmar; locality: Sheltered coral reef located on the coastline of Egypt (Mangrove Bay), 29 km south of the coastal city of El Qoseir; minimumDepthInMeters: 7.2; maximumDepthInMeters: 9.5; verbatimLatitude: N25°52.183′; verbatimLongitude: E034°25.140′; decimalLatitude: 25.8697167; decimalLongitude: 34.419; geodeticDatum: WGS84; **Event:** samplingEffort: 3 x 20 m Line Intercept Transects; eventDate: 25-10-2023; year: 2023; habitat: sheltered deeper reef slope; **Record Level:** institutionCode: UNIVIE; collectionCode: Coral_Reef-Mangrove_Bay-Egypt-2023; basisOfRecord: HumanObservation

##### Conservation status

Unknown

##### Notes

Fig. 62; one distinguished morphotype.

#### Stylophora subseriata

(Ehrenberg, 1834)

01CE090A-70D1-530F-B6CF-D456B9C5C3DD

##### Materials

- **Type status:** Other material. **Occurrence:** occurrenceRemarks: Observed and photographed on a current-exposed reef edge; occurrenceStatus: present; occurrenceID: 7E79C371-D733-5FAA-8BFD-55BEC7CF8112; **Taxon:** scientificNameID: urn:lsid:marinespecies.org:taxname:206980; scientificName: *Stylophorasubseriata*; kingdom: Animalia; phylum: Cnidaria; class: Anthozoa; taxonRank: Species; **Location:** continent: Africa; waterBody: Red Sea; country: Egypt; stateProvince: Muhafazah al Bahr al Ahmar; locality: Current-exposed coral reef located on the coastline of Egypt (Mangrove Bay), 29 km south of the coastal city of El Qoseir; verbatimDepth: 1; verbatimLatitude: N25°52.209′; verbatimLongitude: E034°25.181′; decimalLatitude: 25.87015; decimalLongitude: 34.4196833; geodeticDatum: WGS84; **Event:** samplingEffort: 3 x 20 m Line Intercept Transects; eventDate: 24-10-2023; year: 2023; habitat: current-exposed reef edge; **Record Level:** institutionCode: UNIVIE; collectionCode: Coral_Reef-Mangrove_Bay-Egypt-2023; basisOfRecord: HumanObservation
- **Type status:** Other material. **Occurrence:** occurrenceRemarks: Observed and photographed on a current-exposed deeper reef slope; occurrenceStatus: present; occurrenceID: 19A420FA-59A0-5482-8717-8BA813543381; **Taxon:** scientificNameID: urn:lsid:marinespecies.org:taxname:206980; scientificName: *Stylophorasubseriata*; kingdom: Animalia; phylum: Cnidaria; class: Anthozoa; taxonRank: Species; **Location:** continent: Africa; waterBody: Red Sea; country: Egypt; stateProvince: Muhafazah al Bahr al Ahmar; locality: Current-exposed coral reef located on the coastline of Egypt (Mangrove Bay), 29 km south of the coastal city of El Qoseir; minimumDepthInMeters: 8.5; maximumDepthInMeters: 8.8; verbatimLatitude: N25°52.209′; verbatimLongitude: E034°25.181′; decimalLatitude: 25.87015; decimalLongitude: 34.4196833; geodeticDatum: WGS84; **Event:** samplingEffort: 3 x 20 m Line Intercept Transects; eventDate: 22-10-2023; year: 2023; habitat: current-exposed deeper reef slope; **Record Level:** institutionCode: UNIVIE; collectionCode: Coral_Reef-Mangrove_Bay-Egypt-2023; basisOfRecord: HumanObservation

##### Conservation status

Least Concern

##### Notes

Fig. 63 .

#### Poritidae

Gray, 1840

0EEEB5DB-9B93-5D62-B0B6-8D5D07CDCAC8

#### Porites rus

(Forskål, 1775)

054BE2DB-7132-56A3-8C18-A3771BB9115E

##### Materials

- **Type status:** Other material. **Occurrence:** occurrenceRemarks: Observed and photographed on a current-exposed reef edge; occurrenceStatus: present; occurrenceID: 723910B3-124D-5523-8DCC-FC4AB8A548DC; **Taxon:** scientificNameID: urn:lsid:marinespecies.org:taxname:207231; scientificName: *Poritesrus*; kingdom: Animalia; phylum: Cnidaria; class: Anthozoa; taxonRank: Species; **Location:** continent: Africa; waterBody: Red Sea; country: Egypt; stateProvince: Muhafazah al Bahr al Ahmar; locality: Current-exposed coral reef located on the coastline of Egypt (Mangrove Bay), 29 km south of the coastal city of El Qoseir; verbatimDepth: 1; verbatimLatitude: N25°52.209′; verbatimLongitude: E034°25.181′; decimalLatitude: 25.87015; decimalLongitude: 34.4196833; geodeticDatum: WGS84; **Event:** samplingEffort: 3 x 20 m Line Intercept Transects; eventDate: 24-10-2023; year: 2023; habitat: current-exposed reef edge; **Record Level:** institutionCode: UNIVIE; collectionCode: Coral_Reef-Mangrove_Bay-Egypt-2023; basisOfRecord: HumanObservation
- **Type status:** Other material. **Occurrence:** occurrenceRemarks: Observed and photographed on a current-exposed shallow reef slope; occurrenceStatus: present; occurrenceID: 11B77B77-255E-516C-BF7D-E2E198ED30DD; **Taxon:** scientificNameID: urn:lsid:marinespecies.org:taxname:207231; scientificName: *Poritesrus*; kingdom: Animalia; phylum: Cnidaria; class: Anthozoa; taxonRank: Species; **Location:** continent: Africa; waterBody: Red Sea; country: Egypt; stateProvince: Muhafazah al Bahr al Ahmar; locality: Current-exposed coral reef located on the coastline of Egypt (Mangrove Bay), 29 km south of the coastal city of El Qoseir; verbatimDepth: 4.3; verbatimLatitude: N25°52.209′; verbatimLongitude: E034°25.181′; decimalLatitude: 25.87015; decimalLongitude: 34.4196833; geodeticDatum: WGS84; **Event:** samplingEffort: 3 x 20 m Line Intercept Transects; eventDate: 24-10-2023; year: 2023; habitat: current-exposed shallow reef slope; **Record Level:** institutionCode: UNIVIE; collectionCode: Coral_Reef-Mangrove_Bay-Egypt-2023; basisOfRecord: HumanObservation
- **Type status:** Other material. **Occurrence:** occurrenceRemarks: Observed and photographed on a current-exposed deeper reef slope; occurrenceStatus: present; occurrenceID: 1CE202A1-387E-55AC-9B96-42AB11BDA1CD; **Taxon:** scientificNameID: urn:lsid:marinespecies.org:taxname:207231; scientificName: *Poritesrus*; kingdom: Animalia; phylum: Cnidaria; class: Anthozoa; taxonRank: Species; **Location:** continent: Africa; waterBody: Red Sea; country: Egypt; stateProvince: Muhafazah al Bahr al Ahmar; locality: Current-exposed coral reef located on the coastline of Egypt (Mangrove Bay), 29 km south of the coastal city of El Qoseir; minimumDepthInMeters: 8.5; maximumDepthInMeters: 8.8; verbatimLatitude: N25°52.209′; verbatimLongitude: E034°25.181′; decimalLatitude: 25.87015; decimalLongitude: 34.4196833; geodeticDatum: WGS84; **Event:** samplingEffort: 3 x 20 m Line Intercept Transects; eventDate: 22-10-2023; year: 2023; habitat: current-exposed deeper reef slope; **Record Level:** institutionCode: UNIVIE; collectionCode: Coral_Reef-Mangrove_Bay-Egypt-2023; basisOfRecord: HumanObservation
- **Type status:** Other material. **Occurrence:** occurrenceRemarks: Observed and photographed on a sheltered reef edge; occurrenceStatus: present; occurrenceID: B9390B3A-C4FB-5967-B30B-B47B73B0E6C0; **Taxon:** scientificNameID: urn:lsid:marinespecies.org:taxname:207231; scientificName: *Poritesrus*; kingdom: Animalia; phylum: Cnidaria; class: Anthozoa; taxonRank: Species; **Location:** continent: Africa; waterBody: Red Sea; country: Egypt; stateProvince: Muhafazah al Bahr al Ahmar; locality: Sheltered coral reef located on the coastline of Egypt (Mangrove Bay), 29 km south of the coastal city of El Qoseir; verbatimDepth: 1; verbatimLatitude: N25°52.183′; verbatimLongitude: E034°25.140′; decimalLatitude: 25.8697167; decimalLongitude: 34.419; geodeticDatum: WGS84; **Event:** samplingEffort: 3 x 20 m Line Intercept Transects; eventDate: 20-10-2023; year: 2023; habitat: sheltered reef edge; **Record Level:** institutionCode: UNIVIE; collectionCode: Coral_Reef-Mangrove_Bay-Egypt-2023; basisOfRecord: HumanObservation
- **Type status:** Other material. **Occurrence:** occurrenceRemarks: Observed and photographed on a sheltered shallow reef slope; occurrenceStatus: present; occurrenceID: 5F8EEFED-9A77-5E76-9ACC-387F138767F7; **Taxon:** scientificNameID: urn:lsid:marinespecies.org:taxname:207231; scientificName: *Poritesrus*; kingdom: Animalia; phylum: Cnidaria; class: Anthozoa; taxonRank: Species; **Location:** continent: Africa; waterBody: Red Sea; country: Egypt; stateProvince: Muhafazah al Bahr al Ahmar; locality: Sheltered coral reef located on the coastline of Egypt (Mangrove Bay), 29 km south of the coastal city of El Qoseir; minimumDepthInMeters: 3.7; maximumDepthInMeters: 4.75; verbatimLatitude: N25°52.183′; verbatimLongitude: E034°25.140′; decimalLatitude: 25.8697167; decimalLongitude: 34.419; geodeticDatum: WGS84; **Event:** samplingEffort: 3 x 20 m Line Intercept Transects; eventDate: 23-10-2023; year: 2023; habitat: sheltered shallow reef slope; **Record Level:** institutionCode: UNIVIE; collectionCode: Coral_Reef-Mangrove_Bay-Egypt-2023; basisOfRecord: HumanObservation
- **Type status:** Other material. **Occurrence:** occurrenceRemarks: Observed and photographed on a sheltered deeper reef slope; occurrenceStatus: present; occurrenceID: F69D6064-B51C-5343-A8E5-528626AAF490; **Taxon:** scientificNameID: urn:lsid:marinespecies.org:taxname:207231; scientificName: *Poritesrus*; kingdom: Animalia; phylum: Cnidaria; class: Anthozoa; taxonRank: Species; **Location:** continent: Africa; waterBody: Red Sea; country: Egypt; stateProvince: Muhafazah al Bahr al Ahmar; locality: Sheltered coral reef located on the coastline of Egypt (Mangrove Bay), 29 km south of the coastal city of El Qoseir; minimumDepthInMeters: 7.2; maximumDepthInMeters: 9.5; verbatimLatitude: N25°52.183′; verbatimLongitude: E034°25.140′; decimalLatitude: 25.8697167; decimalLongitude: 34.419; geodeticDatum: WGS84; **Event:** samplingEffort: 3 x 20 m Line Intercept Transects; eventDate: 23-10-2023; year: 2023; habitat: sheltered deeper reef slope; **Record Level:** institutionCode: UNIVIE; collectionCode: Coral_Reef-Mangrove_Bay-Egypt-2023; basisOfRecord: HumanObservation

##### Conservation status

Least Concern

##### Notes

Fig. 64 .

#### Porites spp.

Link, 1807

9CAA29F5-0B4E-5963-A8DB-74097420E31F

##### Materials

- **Type status:** Other material. **Occurrence:** occurrenceRemarks: Observed and photographed on a current-exposed reef edge; occurrenceStatus: present; occurrenceID: 6029AA53-7CFC-56B1-9652-4C0E6AB5B56E; **Taxon:** scientificNameID: urn:lsid:marinespecies.org:taxname:206485; scientificName: *Porites* spp.; kingdom: Animalia; phylum: Cnidaria; class: Anthozoa; taxonRank: Genus; **Location:** continent: Africa; waterBody: Red Sea; country: Egypt; stateProvince: Muhafazah al Bahr al Ahmar; locality: Current-exposed coral reef located on the coastline of Egypt (Mangrove Bay), 29 km south of the coastal city of El Qoseir; verbatimDepth: 1; verbatimLatitude: N25°52.209′; verbatimLongitude: E034°25.181′; decimalLatitude: 25.87015; decimalLongitude: 34.4196833; geodeticDatum: WGS84; **Event:** samplingEffort: 3 x 20 m Line Intercept Transects; eventDate: 22-10-2023; year: 2023; habitat: current-exposed reef edge; **Record Level:** institutionCode: UNIVIE; collectionCode: Coral_Reef-Mangrove_Bay-Egypt-2023; basisOfRecord: HumanObservation
- **Type status:** Other material. **Occurrence:** occurrenceRemarks: Observed and photographed on a current-exposed shallow reef slope; occurrenceStatus: present; occurrenceID: 13966F0F-B746-5AF7-9BD4-4EA9060B6EAA; **Taxon:** scientificNameID: urn:lsid:marinespecies.org:taxname:206485; scientificName: *Porites* spp.; kingdom: Animalia; phylum: Cnidaria; class: Anthozoa; taxonRank: Genus; **Location:** continent: Africa; waterBody: Red Sea; country: Egypt; stateProvince: Muhafazah al Bahr al Ahmar; locality: Current-exposed coral reef located on the coastline of Egypt (Mangrove Bay), 29 km south of the coastal city of El Qoseir; verbatimDepth: 4.3; verbatimLatitude: N25°52.209′; verbatimLongitude: E034°25.181′; decimalLatitude: 25.87015; decimalLongitude: 34.4196833; geodeticDatum: WGS84; **Event:** samplingEffort: 3 x 20 m Line Intercept Transects; eventDate: 22-10-2023; year: 2023; habitat: current-exposed shallow reef slope; **Record Level:** institutionCode: UNIVIE; collectionCode: Coral_Reef-Mangrove_Bay-Egypt-2023; basisOfRecord: HumanObservation
- **Type status:** Other material. **Occurrence:** occurrenceRemarks: Observed and photographed on a current-exposed deeper reef slope; occurrenceStatus: present; occurrenceID: 551F0ED2-48C7-5CB1-9DE1-9D31D4F30AD6; **Taxon:** scientificNameID: urn:lsid:marinespecies.org:taxname:206485; scientificName: *Porites* spp.; kingdom: Animalia; phylum: Cnidaria; class: Anthozoa; taxonRank: Genus; **Location:** continent: Africa; waterBody: Red Sea; country: Egypt; stateProvince: Muhafazah al Bahr al Ahmar; locality: Current-exposed coral reef located on the coastline of Egypt (Mangrove Bay), 29 km south of the coastal city of El Qoseir; minimumDepthInMeters: 8.5; maximumDepthInMeters: 8.8; verbatimLatitude: N25°52.209′; verbatimLongitude: E034°25.181′; decimalLatitude: 25.87015; decimalLongitude: 34.4196833; geodeticDatum: WGS84; **Event:** samplingEffort: 3 x 20 m Line Intercept Transects; eventDate: 22-10-2023; year: 2023; habitat: current-exposed deeper reef slope; **Record Level:** institutionCode: UNIVIE; collectionCode: Coral_Reef-Mangrove_Bay-Egypt-2023; basisOfRecord: HumanObservation
- **Type status:** Other material. **Occurrence:** occurrenceRemarks: Observed and photographed on a sheltered reef edge; occurrenceStatus: present; occurrenceID: 60A58FBC-57DF-5944-9469-D5B5B144DCA5; **Taxon:** scientificNameID: urn:lsid:marinespecies.org:taxname:206485; scientificName: *Porites* spp.; kingdom: Animalia; phylum: Cnidaria; class: Anthozoa; taxonRank: Genus; **Location:** continent: Africa; waterBody: Red Sea; country: Egypt; stateProvince: Muhafazah al Bahr al Ahmar; locality: Sheltered coral reef located on the coastline of Egypt (Mangrove Bay), 29 km south of the coastal city of El Qoseir; verbatimDepth: 1; verbatimLatitude: N25°52.183′; verbatimLongitude: E034°25.140′; decimalLatitude: 25.8697167; decimalLongitude: 34.419; geodeticDatum: WGS84; **Event:** samplingEffort: 3 x 20 m Line Intercept Transects; eventDate: 19-10-2023; year: 2023; habitat: sheltered reef edge; **Record Level:** institutionCode: UNIVIE; collectionCode: Coral_Reef-Mangrove_Bay-Egypt-2023; basisOfRecord: HumanObservation
- **Type status:** Other material. **Occurrence:** occurrenceRemarks: Observed and photographed on a sheltered shallow reef slope; occurrenceStatus: present; occurrenceID: 239B0E32-5A53-5EB1-BA54-C9C8FD270439; **Taxon:** scientificNameID: urn:lsid:marinespecies.org:taxname:206485; scientificName: *Porites* spp.; kingdom: Animalia; phylum: Cnidaria; class: Anthozoa; taxonRank: Genus; **Location:** continent: Africa; waterBody: Red Sea; country: Egypt; stateProvince: Muhafazah al Bahr al Ahmar; locality: Sheltered coral reef located on the coastline of Egypt (Mangrove Bay), 29 km south of the coastal city of El Qoseir; minimumDepthInMeters: 3.7; maximumDepthInMeters: 4.75; verbatimLatitude: N25°52.183′; verbatimLongitude: E034°25.140′; decimalLatitude: 25.8697167; decimalLongitude: 34.419; geodeticDatum: WGS84; **Event:** samplingEffort: 3 x 20 m Line Intercept Transects; eventDate: 23-10-2023; year: 2023; habitat: sheltered shallow reef slope; **Record Level:** institutionCode: UNIVIE; collectionCode: Coral_Reef-Mangrove_Bay-Egypt-2023; basisOfRecord: HumanObservation
- **Type status:** Other material. **Occurrence:** occurrenceRemarks: Observed and photographed on a sheltered deeper reef slope; occurrenceStatus: present; occurrenceID: C745D3A2-8AED-5F33-94EC-827970DD19A6; **Taxon:** scientificNameID: urn:lsid:marinespecies.org:taxname:206485; scientificName: *Porites* spp.; kingdom: Animalia; phylum: Cnidaria; class: Anthozoa; taxonRank: Genus; **Location:** continent: Africa; waterBody: Red Sea; country: Egypt; stateProvince: Muhafazah al Bahr al Ahmar; locality: Sheltered coral reef located on the coastline of Egypt (Mangrove Bay), 29 km south of the coastal city of El Qoseir; minimumDepthInMeters: 7.2; maximumDepthInMeters: 9.5; verbatimLatitude: N25°52.183′; verbatimLongitude: E034°25.140′; decimalLatitude: 25.8697167; decimalLongitude: 34.419; geodeticDatum: WGS84; **Event:** samplingEffort: 3 x 20 m Line Intercept Transects; eventDate: 23-10-2023; year: 2023; habitat: sheltered deeper reef slope; **Record Level:** institutionCode: UNIVIE; collectionCode: Coral_Reef-Mangrove_Bay-Egypt-2023; basisOfRecord: HumanObservation

##### Conservation status

Unknown

##### Notes

Fig. 65; one distinguished morphotype.

#### Psammocoridae

Chevalier & L. Beauvais, 1987

633B0DB3-8D3A-5FF5-B1E8-4991BC6124FE

#### Psammocora profundacella

Gardiner, 1898

2C7E2446-37DB-53EF-BE9D-ECA9E5B08FA4

##### Materials

- **Type status:** Other material. **Occurrence:** occurrenceRemarks: Observed and photographed on a current-exposed deeper reef slope; occurrenceStatus: present; occurrenceID: 5F2A2A2C-5B7D-505C-98A1-36C1F7F9A216; **Taxon:** scientificNameID: urn:lsid:marinespecies.org:taxname:207271; scientificName: *Psammocoraprofundacella*; kingdom: Animalia; phylum: Cnidaria; class: Anthozoa; taxonRank: Species; **Location:** continent: Africa; waterBody: Red Sea; country: Egypt; stateProvince: Muhafazah al Bahr al Ahmar; locality: Current-exposed coral reef located on the coastline of Egypt (Mangrove Bay), 29 km south of the coastal city of El Qoseir; minimumDepthInMeters: 8.5; maximumDepthInMeters: 8.8; verbatimLatitude: N25°52.209′; verbatimLongitude: E034°25.181′; decimalLatitude: 25.87015; decimalLongitude: 34.4196833; geodeticDatum: WGS84; **Event:** samplingEffort: 3 x 20 m Line Intercept Transects; eventDate: 22-10-2023; year: 2023; habitat: current-exposed deeper reef slope; **Record Level:** institutionCode: UNIVIE; collectionCode: Coral_Reef-Mangrove_Bay-Egypt-2023; basisOfRecord: HumanObservation
- **Type status:** Other material. **Occurrence:** occurrenceRemarks: Observed and photographed on a sheltered deeper reef slope; occurrenceStatus: present; occurrenceID: 70CE6CA1-CBD7-5B36-A199-C3C573E0E829; **Taxon:** scientificNameID: urn:lsid:marinespecies.org:taxname:207271; scientificName: *Psammocoraprofundacella*; kingdom: Animalia; phylum: Cnidaria; class: Anthozoa; taxonRank: Species; **Location:** continent: Africa; waterBody: Red Sea; country: Egypt; stateProvince: Muhafazah al Bahr al Ahmar; locality: Sheltered coral reef located on the coastline of Egypt (Mangrove Bay), 29 km south of the coastal city of El Qoseir; minimumDepthInMeters: 7.2; maximumDepthInMeters: 9.5; verbatimLatitude: N25°52.183′; verbatimLongitude: E034°25.140′; decimalLatitude: 25.8697167; decimalLongitude: 34.419; geodeticDatum: WGS84; **Event:** samplingEffort: 3 x 20 m Line Intercept Transects; eventDate: 25-10-2023; year: 2023; habitat: sheltered deeper reef slope; **Record Level:** institutionCode: UNIVIE; collectionCode: Coral_Reef-Mangrove_Bay-Egypt-2023; basisOfRecord: HumanObservation

##### Conservation status

Least Concern

##### Notes

Fig. 66 .

#### Psammocora sp.

Dana, 1846

A09A1802-765F-5845-BA77-3A80EAE7D5E6

##### Materials

- **Type status:** Other material. **Occurrence:** occurrenceRemarks: Observed on a sheltered shallow reef slope; occurrenceID: C76AFA44-6297-551A-926A-D2A8DD25EFB4; **Taxon:** scientificNameID: urn:lsid:marinespecies.org:taxname:204148; scientificName: *Psammocora* sp.; kingdom: Animalia; phylum: Cnidaria; class: Anthozoa; taxonRank: Genus; **Location:** continent: Africa; waterBody: Red Sea; country: Egypt; stateProvince: Muhafazah al Bahr al Ahmar; locality: Sheltered coral reef located on the coastline of Egypt (Mangrove Bay), 29 km south of the coastal city of El Qoseir; minimumDepthInMeters: 3.7; maximumDepthInMeters: 4.75; verbatimLatitude: N25°52.183′; verbatimLongitude: E034°25.140′; decimalLatitude: 25.8697167; decimalLongitude: 34.419; geodeticDatum: WGS84; **Event:** samplingEffort: 3 x 20 m Line Intercept Transects; eventDate: 25-10-2023; year: 2023; habitat: sheltered shallow reef slope; **Record Level:** institutionCode: UNIVIE; collectionCode: Coral_Reef-Mangrove_Bay-Egypt-2023; basisOfRecord: HumanObservation

##### Conservation status

Unknown

## Analysis

We observed and identified a total of 68 scleractinian coral species belonging to 29 genera and 14 families (Table 2). Amongst these, the families with the highest number of observed species were Merulinidae (25 species) and Acroporidae (11 species). Scleractinian coral colonies from the genera *Acropora* and *Porites*, as well as the species *Goniastreaedwardsi*, *Pocilloporaverrucosa* and *Poritesrus*, were found in all six habitats. Coral colonies described as *Porites* sp. dominated at the sheltered site across all depths and at the deeper reef slope of the current-exposed site, while *Pocilloporaverrucosa* was the most abundant coral species at the current-exposed site's reef edge and shallow slope. At all depths, the current-exposed site was more species-rich than the sheltered site, with the deeper reef slope hosting 43 species, the highest richness observed, while the sheltered reef edge had the lowest, with 12 species (Fig. 67).

The distribution of coral growth forms across the sheltered and current-exposed sites is depicted in Fig. 68 and Table 3. At the sheltered reef edge, submassive corals were most prevalent, followed by branching and encrusting growth forms. On the sheltered shallow reef slope, submassive corals remained dominant, followed by encrusting and branching corals, branching and digitate *Acropora* and free-living corals. At the sheltered deeper reef slope, massive corals became dominant, followed by encrusting, submassive, and branching corals, branching *Acropora* and free-living corals.

At the current-exposed reef edge, branching corals were most common, followed by submassive and massive corals, branching *Acropora* and encrusting corals. On the current-exposed shallow reef slope, branching corals remained dominant, followed by encrusting, massive and submassive corals, branching and digitate *Acropora*, free-living and foliaceous corals. At the deeper reef slope, massive corals were most abundant, followed by encrusting corals, branching *Acropora*, submassive and branching corals, digitate *Acropora* and foliaceous corals (Fig. 68; Table 3).

In addition to examining coral diversity, distribution and coral morphology, we considered the conservation status of the identified coral species. According to the latest IUCN Red List assessment from November 2024, which evaluated 892 warm-water reef-building coral species, 44% are classified as threatened (International Union for Conservation of Nature (IUCN) 2024). In our evaluation of 68 coral species, we found that 29 species (43%) are categorised as "Least Concern", 18 species (26%) as "Near Threatened", six species (9%) as "Vulnerable" and 15 (22%) were of unknown conservation status. The species classified as "Vulnerable" include *Acroporahemprichii*, *Montiporamaeandrina*, *Acanthastreahemprichii*, *Dipsastraeafaviaformis*, *Pavonadiffluens* and *Turbinariamesenterina*.

The relative percentage of biogenic and abiogenic components also showed variations across all six habitats (Fig. 69; Table 4). At the sheltered reef edge, algal cover was most prevalent, followed by scleractinian coral cover, *Millepora* cover, dead scleractinian coral cover, other benthic fauna cover and soft coral cover. On both the sheltered shallow and deeper reef slope, algal cover remained dominant, followed by, scleractinian coral cover, soft coral cover, dead scleractinian coral cover, other benthic fauna cover, bare substrate cover and *Millepora* cover.

At the current-exposed reef edge, algal cover was the most common, followed by *Millepora* cover, scleractinian coral cover, other benthic fauna cover and dead scleractinian coral cover. On the current-exposed shallow reef slope, scleractinian coral cover was most abundant, followed by algal cover, bare substrate cover, soft coral cover, dead scleractinian coral cover, other benthic fauna cover and *Millepora* cover. On the current-exposed deeper reef slope, algal cover was dominant, followed by scleractinian coral cover, soft coral cover, bare substrate cover, dead scleractinian coral cover, other benthic fauna cover and *Millepora* cover (Fig. 69; Table 4).

### Taxonomic Uncertainties

Species identification was primarily based on photographic evidence, which, though generally reliable, lacks the precision of genetic analysis. In our transects, we identified coral colonies from the genus *Stylocoeniella* (Fig. 19), which includes two recognised species in the Indo-Pacific: *S.guentheri* and *S.armata* (Veron and Pichon 1976, Scheer and Pillai 1983, Sheppard and Sheppard 1991). Both species display encrusting growth forms with widely-spaced corallites. To distinguish between the two species, one should examine the size of the two septa cycles. In *S.guentheri*, the two septa cycles are unequal in size, whereas in *S.armata*, they are equal in size (Scheer and Pillai 1983, Sheppard and Sheppard 1991). Based on our photographs, we identified the colonies as S.cf.guentheri, acknowledging *S.armata* as a possible alternative identification, based on the morphological traits.

Furthermore, we recorded colonies from the genus *Ctenactis* (Fig. 24). We considered two possible species: *C.crassa* and *C.echinata*, both of which are free-living corals with elongated polyps and septal teeth (Veron and Pichon 1979, Hoeksema 1989). They primarily differ in stomatism, with *C.crassa* being polystomatous and *C.echinata* monostomatous. Based on the absence of multiple polyp mouths observed in the images, we tentatively identified the colonies as *C.cf.crassa*, though *C.echinata* remains a plausible alternative.

*Paraechinophyllia*, a genus first described by Arrigoni et al. (2019), shares many morphological similarities with *Echinophyllia*, particularly in terms of its encrusting growth form and corallite structure. Molecular analysis is required to definitively distinguish between these genera, as traditional morphological traits may not provide sufficient resolution. Therefore, it is possible that some colonies we identified as *Echinophyllia* (Fig. 30) could belong to the genus *Paraechinophyllia*.

**Figure 61.**
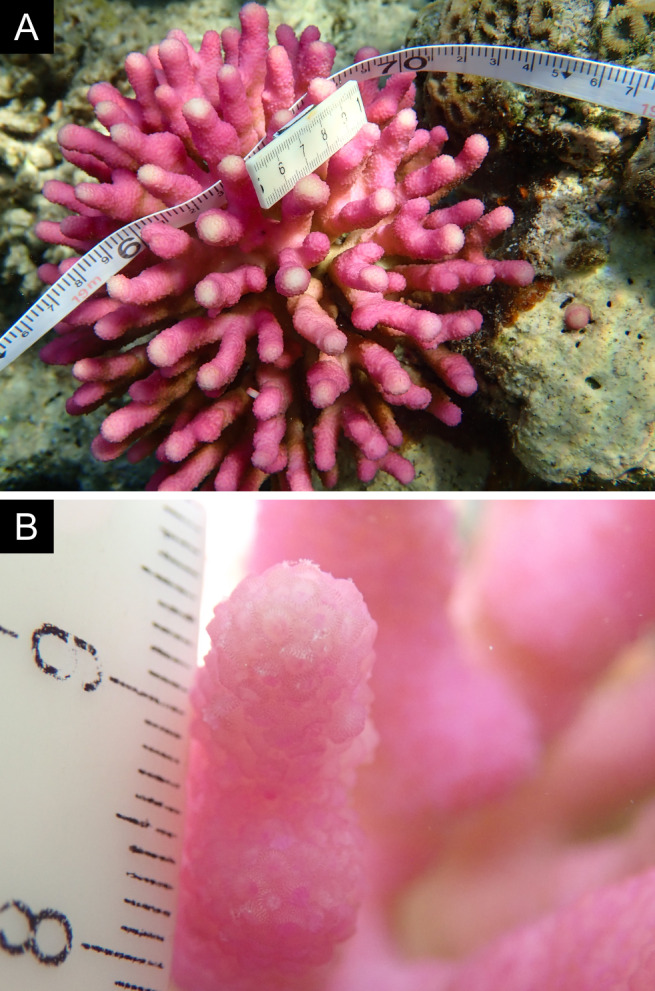
The growth form and appearance of *Stylophorapistillata* in the reef are shown in (**A**), while (**B**) provides a close-up of the colony. Photo credits: Antonia Auer, Theda Schöchtner, Gözde Özer.

**Figure 62.**
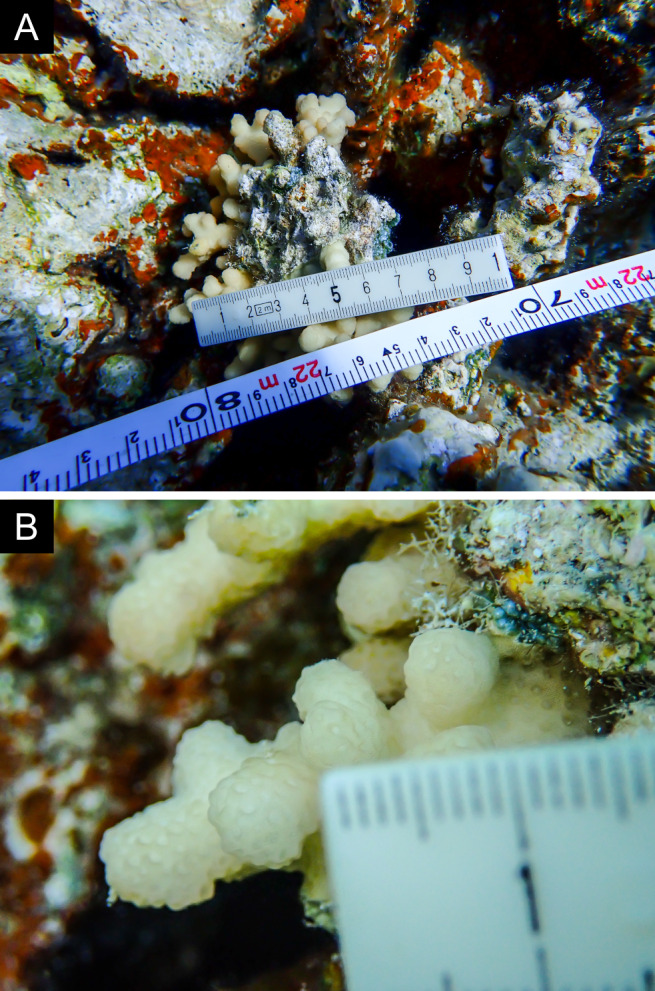
Amongst the *Stylophora* colonies identified only to genus level, one morphotype (**A**—**B**) was distinguished. Photo credits: Theres Koch.

**Figure 63.**
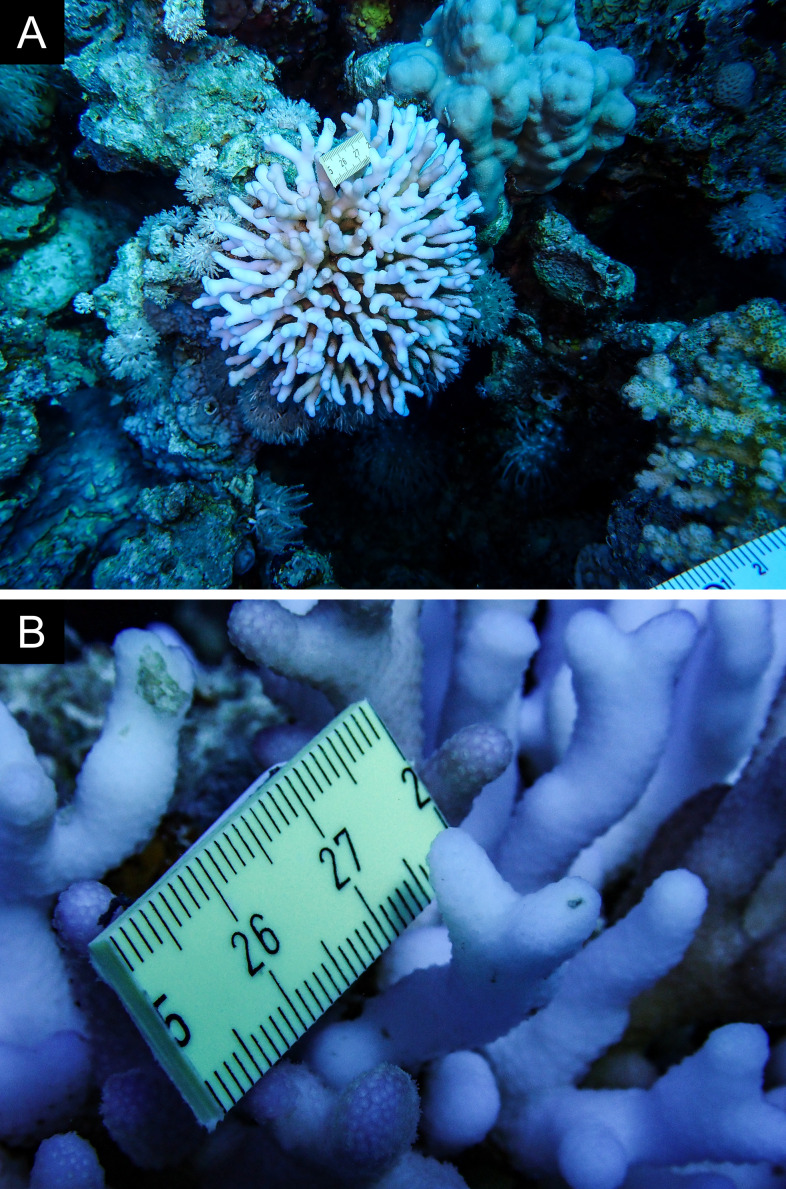
The growth form and appearance of *Stylophorasubseriata* in the reef are shown in (**A**), while (**B**) provides a close-up of the colony. Photo credits: Theres Koch.

**Figure 65.**
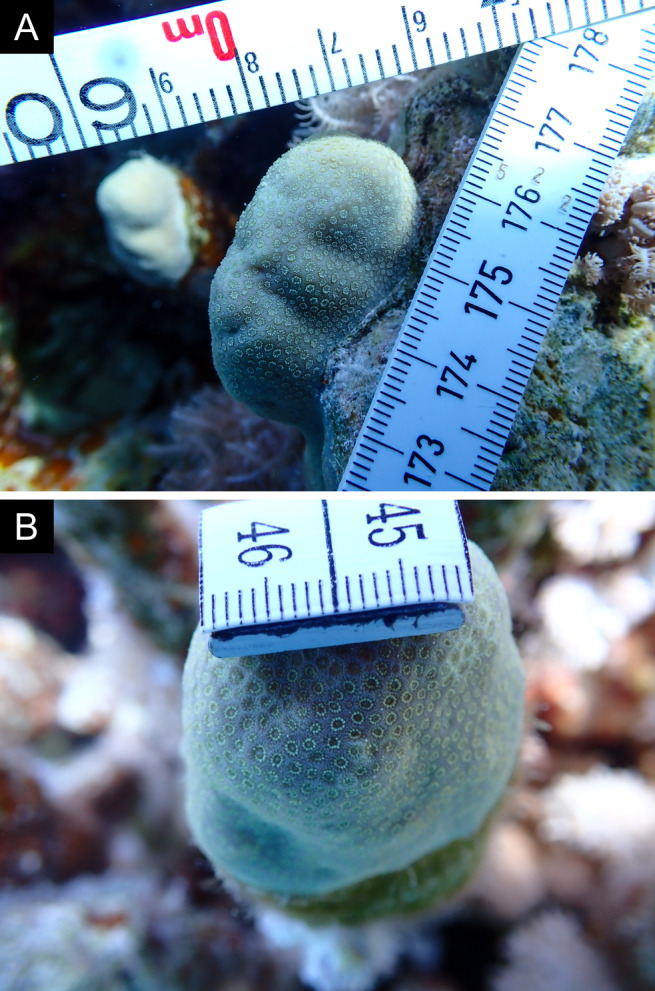
Amongst the *Porites* colonies identified only to genus level, one morphotype (**A**—**B**) was distinguished. Photo credits: Joseph Wallace Daurella.

We observed colonies from the genus *Stylophora*, identifying multiple species (Figs 60, 61, 63). *Stylophora* species in the Red Sea and the Indian Ocean display significant morphological variability, with six regional ecotypes (Scheer and Pillai 1983, Arrigoni et al. 2016a), including *S.kuehlmanni* (Fig. 60). *S.kuehlmanni* typically forms prostrate colonies with thin, irregular, anastomosing branches and tubular corallites, giving the branches a spiny appearance, while *S.pistillata* features thicker branches and primarily conical or hooded corallites (Fig. 61). Based on the thinner branches and prominent corallite hoods in our observations, we identified some colonies of Stylophora as S.cf.kuehlmanni, though we also recognise *S.pistillata* as a plausible alternative for these colonies given their morphological characteristics.

## Discussion

This study focused on the coral diversity and coral growth form distribution at Mangrove Bay, a reef system along the Egyptian coastline of the northern Red Sea. Through comprehensive site surveys, we identified a total of 68 hard coral species, highlighting the ecological and taxonomic richness of this region. Notably, the genera *Acropora*, *Goniastrea*, *Pocillopora* and *Porites* were amongst the most frequently observed.

**Figure 67.**
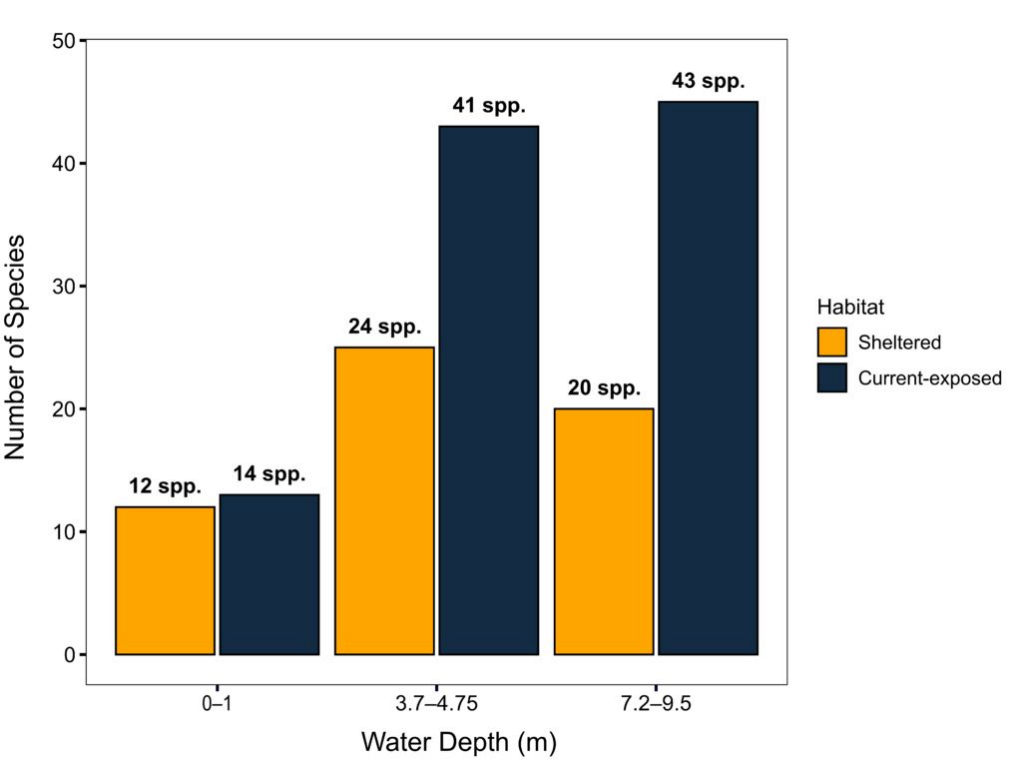
The number of scleractinian coral species across the six different habitats at the study sites at Mangrove Bay.

The overall trend showed that, at both the sheltered and current-exposed sites, scleractinian coral diversity and cover were lower at the reef edge than at the deeper reef slope (Fig. 67, Fig. 69, Table 4). In shallower waters, environmental conditions pose challenges for corals. High turbulence, stronger wave or current action, intense sunlight exposure and elevated water temperatures limit coral growth, whereas rapidly growing species, such as algae (e.g. coralline algae or macro algae), are less impacted by such conditions (Sheppard 1980, Bang et al. 2021). Amongst the species adapted to these more challenging conditions are Hydrozoans of the genus *Millepora*. The three species observed in our study (*Milleporadichotoma*, *Milleporaexaesa* and *Milleporaplatyphylla*) were notably abundant at the reef edge, particularly on the current-exposed side, where high light availability and strong currents provide a favourable habitat (Dubé et al. 2017).

At depths of 7–9.5 m, environmental conditions generally stabilise, characterised by reduced wave energy, diminished sunlight intensity and consistent light availability (Sheppard 1980). These factors promote coral growth and coverage, enabling the development of larger coral colonies (Chadwick and Morrow 2011, Doropoulos et al. 2020). When examining the coverage of dead corals, we found that it peaked at intermediate depths at both study sites, with the shallow reef slopes showing a higher cover of dead corals than the reef edges (Fig. 69; Table 4).

Soft coral cover increased with depth at both study sites. No soft coral cover was observed at the reef edge (Fig. 69; Table 4). Amongst the octocorals identified to the genus level, most belonged to the genus *Heteroxenia*. These corals were particularly abundant on the deeper reef slopes. This distribution can likely be explained by the preference of soft corals from the family *Xeniidae*, such as *Heteroxenia*, for areas with moderate current exposure (e.g. the deeper reef slope) as described by Fabricius and Alderslade (2001).

To thoroughly understand reef ecosystems, we aimed to not only quantify coral diversity, but also areas covered only by bare substrate such as sand patches or rock. The proportions of substrate coverage play an important role in the survival and growth of benthic organisms, influencing species distributions and community dynamics (Kramer et al. 2020). In our case, bare sand and rock surfaces were most prevalent at intermediate depths and completely absent at both reef edges (Fig. 69; Table 4). This suggests that the shallow reef slope experienced weaker currents than the reef edge, which allowed bare substrate to persist (Heyward and Radford 2019). In contrast, at greater depths, environmental conditions become more stable. Consequently, exposed substrate diminishes as the biotic cover, such as coral growth, increases (Chadwick and Morrow 2011, Doropoulos et al. 2020, Bang et al. 2021).

Including substrate coverage as an abiogenic component in assessments of coral diversity across different reef habitats provides a more comprehensive characterisation of reef ecosystems and it serves as a valuable descriptor. It enables researchers to contextualise diversity data and supports further investigations, such as evaluating habitat suitability for specific species or analysing the structural contributions of abiogenic features (Fisher et al. 2015, Roberts et al. 2015). For instance, the amount of substrate coverage can influence the survival and growth of benthic organisms, such as corals, which, in turn, affects species distribution (Duckworth 2015, Sheppard et al. 2017).

**Figure 68.**
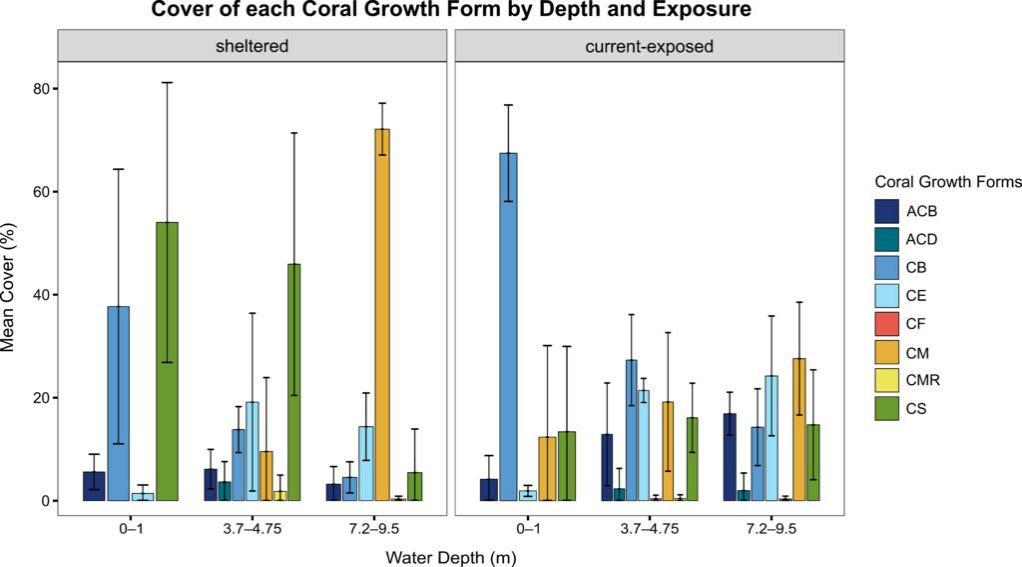
Coral growth form distribution across six different habitats. The percentage of each growth form was calculated, based on its contribution to the total transect length for each habitat. Abbreviations: **ACB** (*Acropora* Coral Branching), **ACD** (*Acropora* Coral Digitate), **CB** (Coral Branching), **CE** (Coral Encrusting), **CF** (Coral Foliaceous), **CM** (Coral Massive), **CMR** (Coral Mushroom) and **CS** (Coral Submassive). Bars display the mean ± standard deviation. Error bars extending 0 were set to 0.

**Figure 69.**
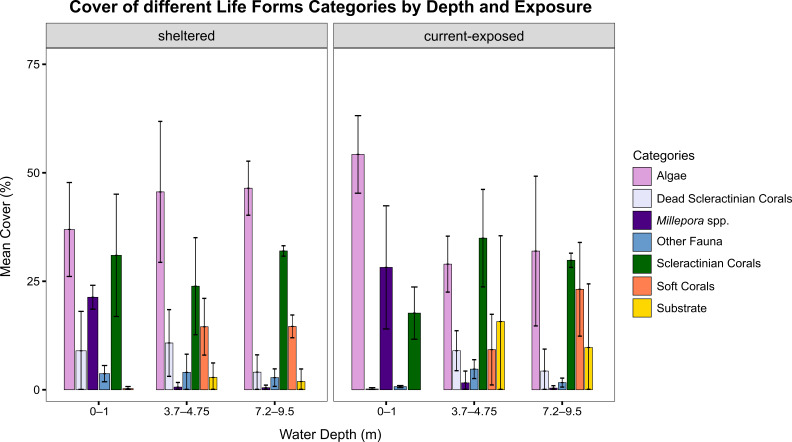
Distribution of various life form categories across the six habitats. Percentages were calculated, based on each category's contribution to the total transect length per habitat (excluding gaps). The "**Algae**" category comprises algae assemblages, coralline algae, macroalgae and turf algae. The "**Dead Scleractinian Coral**" category includes dead corals as well as dead corals overgrown with algae. The "***Millepora* spp**." category encompasses *Milleporadichotoma*, *Milleporaexaesa* and *Milleporaplatyphylla*. The "**Benthic Fauna**" category combines other species, such as molluscs, echinoderms and sponges. The "**Scleractinian Corals**" category includes all living hard corals. The "**Soft Corals**" category encompasses all octocorals that intersected the transect line, including those identified to the genus level, such as *Heteroxenia* and *Rhytisma*. The "**Substrate**" category comprises coral rubble, rock and sand. Bars display the mean ± standard deviation. Error bars extending 0 were set to 0.

Light availability, as well as wave and current exposure, not only influence coral abundance and diversity, but also coral morphology and the distribution of coral growth forms along a reef depth gradient (Halid et al. 2016, Chow et al. 2019, Kramer et al. 2020). In our study, branching corals, such as those from the genera *Acropora* and *Pocillopora*, were observed at all depths, but were particularly abundant at the reef edge, where they accounted for over two-thirds of the total scleractinian coral cover at the current-exposed site (Fig. 68; Table 3). Encrusting corals, including those of the genera *Cyphastrea* and *Montipora*, as well as massive corals like the genus *Goniastrea*, exhibit a more flattened morphology, likely to optimise light capture in low-light environments (Kahng et al. 2010, Roberts et al. 2015), became increasingly common on both shallow and deeper reef slopes. On the deeper reef slope at the sheltered site, massive corals accounted for nearly three-quarters of all living corals. Submassive corals, primarily from the genus *Porites*, were especially abundant at the reef edge of the sheltered site, where they constituted over 50% of the live scleractinian coral cover. This genus is recognised for its ability to thrive under the abundant sunlight available at shallower depths and shows greater tolerance to stressors like thermal stress, particularly at the reef edge (Bruckner and Dempsey 2015).

Comparing our data to species lists by Ammar (2011), Mohammed (2012), Berumen et al. (2019) and Hussein et al. (2023), it becomes clear that the species present in Mangrove Bay are a typical representation of Red Sea coral communities. However, there are some distinct differences between previous checklists and ours. Some species, or even genera, found in Mangrove Bay, such as the genus *Dipsastraea* or the species *Goniastreaedwardsi*, were not listed by Ammar (2011), Mohammed (2012) or Hussein et al. (2023). Conversely, certain species and genera recorded in these previous checklists, such as the genera *Alveopora*, *Euphyllia*, *Fungia* and *Mycedium* and the species *Lobophylliacorymbosa*, were not present in our checklist. These observed differences may be the result of the different sampling protocols employed and habitats investigated. For example, Ammar (2011) conducted a survey across a wide range of depths (1–50 m); Mohammed (2012) employed direct coral sampling and laboratory analysis and included surveying reef flats and sandy patches; Hussein et al. (2023) focused on the impact of urbanisation on coral species diversity by investigating sites affected by human activities. However, the presence and absence of these taxa may also reflect genuine differences, potentially influenced by other factors, such as local environmental conditions.

The data from our study indicate the frequent presence of hard coral species across habitats within Mangrove Bay. Most species were found in both current-exposed and sheltered reef edges, demonstrating their tolerance to various environmental conditions. However, 26 species were found in only one of the six habitats. *Gardineroserisplanulata*, *Leptoserismycetoseroides*, Stylophoracf.kuehlmanni and *Psammocora* sp. were found exclusively at the sheltered shallow reef slope. *Favitesabdita* and *Goniastreastelligera* were found exclusively at the current-exposed reef edge. Montiporacf.grisea, *Leptastreainaequalis*, *Echinophyllia* spp., *Dipsastraeadanai*, *Dipsastraea* spp., *Favitesvasta*, *Goniastrearetiformis*, *Goniastrea* sp., *Pachyserisspeciosa* and *Seriatoporahystrix* were detected only at the current-exposed shallow reef slope. *Montiporacrypta*, *Montiporadanae*, *Turbinariamesenterina*, *Gyrosmiliainterrupta*, *Leptastreabottae*, *Acanthastreahemprichii*, *Oxypora* sp., *Dipsastraeafaviaformis*, *Dipsastraealaxa*, Favitescf.complanata, *Favitesrotundata*, *Merulinascheeri* and *Paramontastraeaperesi* were confined to the current-exposed deeper reef slope.

### Conclusion

Mangrove Bay’s reefs appear to be in relatively good condition and serve as an important site for studying coral reef biodiversity. In this initial survey, we identified 68 scleractinian coral species across six habitats at two reef locations, using Line Intercept Transects and photography, resulting in the first comprehensive record of coral diversity at Mangrove Bay. Genera such as *Acropora*, *Porites* and *Pocillopora* dominated multiple habitats. The observed species richness peaked at the deeper current-exposed reef slope, with 43 coral species and was the lowest at the sheltered reef edge, with 12 species. Habitat-specific patterns in coral growth forms were observed, with branching corals being particularly abundant at reef edges, while encrusting and massive-growing corals became more prevalent with increasing depth. Assessment of benthic abiogenic and biogenic reef components showed widespread algal cover, while *Millepora* coverage peaked at the reef edge, soft coral cover increased with depth and bare substrate was most common at intermediate depths. Conservation evaluations indicated that 43% of coral species are classified as "Least Concern" and 9% are classified as "Vulnerable". Future studies should aim to survey additional habitats within the Mangrove Bay area to provide a more complete picture of local biodiversity. These studies should also include associated fauna and flora, such as reef fish diversity, to gain a holistic understanding of the ecosystem. This report establishes a baseline for monitoring coral species diversity along the Egyptian coastline, thereby supporting long-term conservation initiatives. By providing a detailed overview of the coral species present, this study offers essential data for policy-makers to develop strategies to protect these ecosystems. As highlighted by Reyserhove et al. (2020), species checklists, including the one presented in this study, are vital for understanding biological changes and for designing effective conservation strategies, as they offer detailed species data and associated metadata.

## Supplementary Material

XML Treatment for Acroporidae

XML Treatment for Acropora digitifera

XML Treatment for Acropora hemprichii

XML Treatment for Acropora spp. f. spp.

XML Treatment for Astreopora myriophthalma

XML Treatment for Montipora crypta

XML Treatment for Montipora danae

XML Treatment for Montipora efflorescens

XML Treatment for Montipora cf. grisea

XML Treatment for Montipora maeandrina

XML Treatment for Montipora spp.

XML Treatment for Montipora tuberculosa

XML Treatment for Agariciidae

XML Treatment for Gardineroseris planulata

XML Treatment for Leptoseris mycetoseroides

XML Treatment for Pavona diffluens

XML Treatment for Pavona spp.

XML Treatment for Pavona varians

XML Treatment for Astrocoeniidae

XML Treatment for Stylocoeniella cf. guentheri

XML Treatment for Coscinaraeidae

XML Treatment for Coscinaraea monile

XML Treatment for Dendrophylliidae

XML Treatment for Turbinaria mesenterina

XML Treatment for Euphylliidae

XML Treatment for Galaxea fascicularis

XML Treatment for Gyrosmilia interrupta

XML Treatment for Fungiidae

XML Treatment for Ctenactis cf. crassa

XML Treatment for Leptastreidae

XML Treatment for Leptastrea bottae

XML Treatment for Leptastrea inaequalis

XML Treatment for Leptastrea transversa

XML Treatment for Lobophylliidae

XML Treatment for Acanthastrea hemprichii

XML Treatment for Echinophyllia spp.

XML Treatment for Echinophyllia cf. spp.

XML Treatment for Oxypora sp.

XML Treatment for Merulinidae

XML Treatment for Cyphastrea chalcidicum

XML Treatment for Cyphastrea kausti

XML Treatment for Cyphastrea magna

XML Treatment for Cyphastrea microphthalma

XML Treatment for Cyphastrea spp.

XML Treatment for Dipsastraea danai

XML Treatment for Dipsastraea faviaformis

XML Treatment for Dipsastraea laxa

XML Treatment for Dipsastraea matthaii

XML Treatment for Dipsastraea pallida

XML Treatment for Dipsastraea spp.

XML Treatment for Dipsastraea speciosa

XML Treatment for Echinopora forskaliana

XML Treatment for Echinopora fruticulosa

XML Treatment for Favites abdita

XML Treatment for Favites cf. complanata

XML Treatment for Favites rotundata

XML Treatment for Favites vasta

XML Treatment for Goniastrea edwardsi

XML Treatment for Goniastrea pectinata

XML Treatment for Goniastrea retiformis

XML Treatment for Goniastrea sp.

XML Treatment for Goniastrea stelligera

XML Treatment for Merulina scheeri

XML Treatment for Paramontastraea peresi

XML Treatment for Pachyseridae

XML Treatment for Pachyseris speciosa

XML Treatment for Pocilloporidae

XML Treatment for Pocillopora damicornis

XML Treatment for Pocillopora spp.

XML Treatment for Pocillopora verrucosa

XML Treatment for Seriatopora caliendrum

XML Treatment for Seriatopora hystrix

XML Treatment for Stylophora cf. kuehlmanni

XML Treatment for Stylophora pistillata

XML Treatment for Stylophora spp.

XML Treatment for Stylophora subseriata

XML Treatment for Poritidae

XML Treatment for Porites rus

XML Treatment for Porites spp.

XML Treatment for Psammocoridae

XML Treatment for Psammocora profundacella

XML Treatment for Psammocora sp.

## Figures and Tables

**Figure 3.**
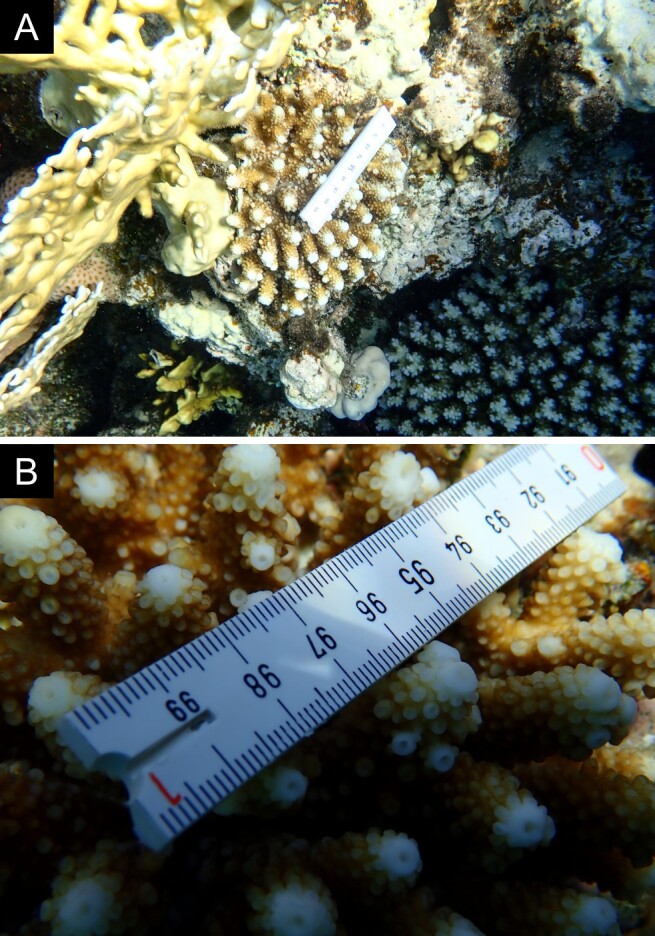
The growth form and appearance of *Acroporadigitifera* in the reef are shown in (**A**), while (**B**) provides a close-up of the colony. Photo credits: Antonia Auer, Theda Schöchtner, Gözde Özer.

**Figure 4.**
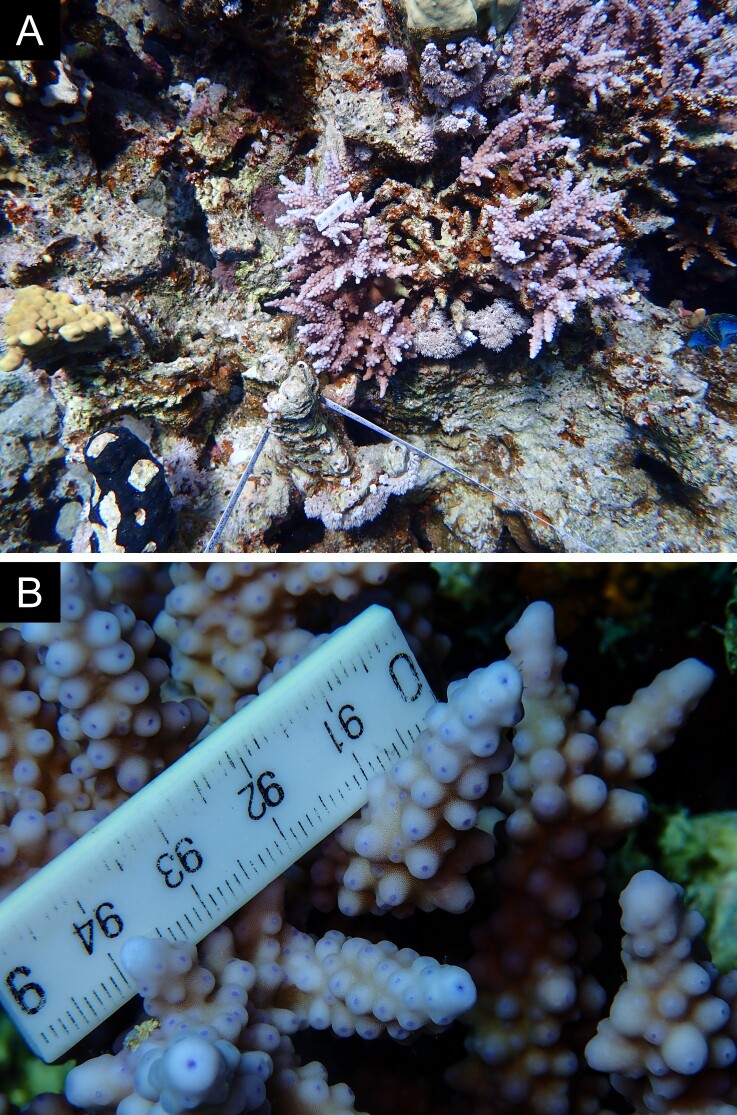
The growth form and appearance of *Acroporahemprichii* in the reef are shown in (**A**), while (**B**) provides a close-up of the colony. Photo credits: Theres Koch.

**Figure 6.**
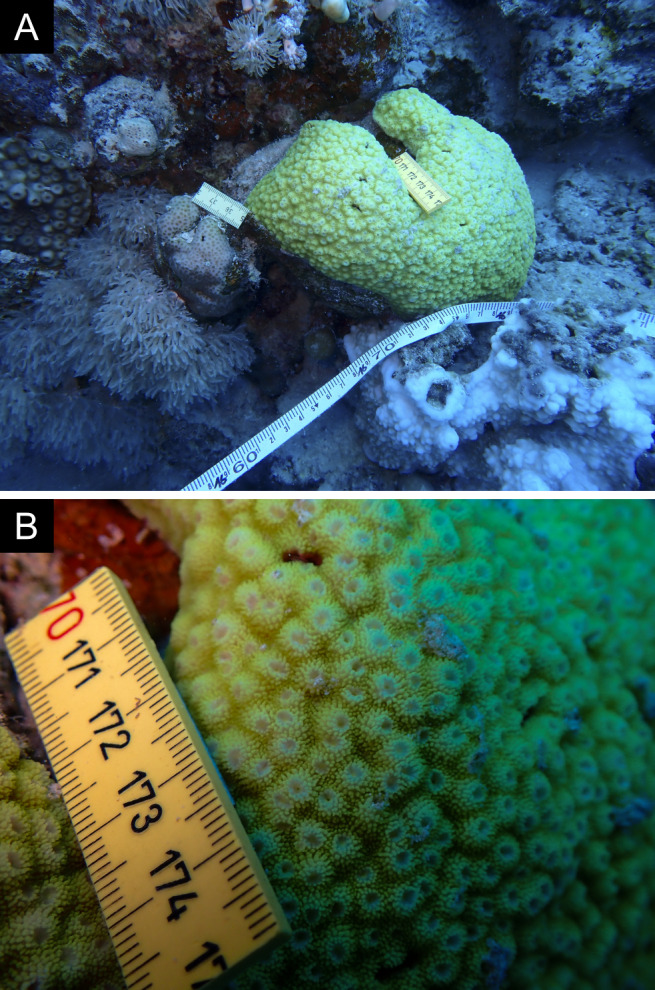
The growth form and appearance of *Astreoporamyriophthalma* in the reef are shown in (**A**), while (**B**) provides a close-up of the colony. Photo credits: Victor Sebastian Scharnhorst.

**Figure 7.**
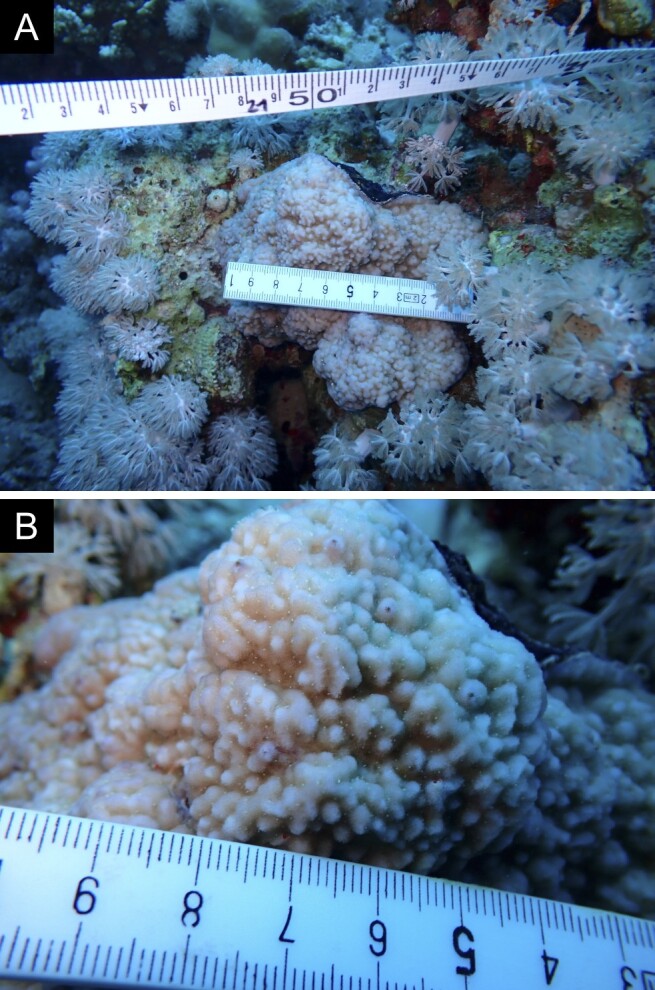
The growth form and appearance of *Montiporacrypta* in the reef are shown in (**A**), while (**B**) provides a close-up of the colony. Photo credits: Victor Sebastian Scharnhorst.

**Figure 8.**
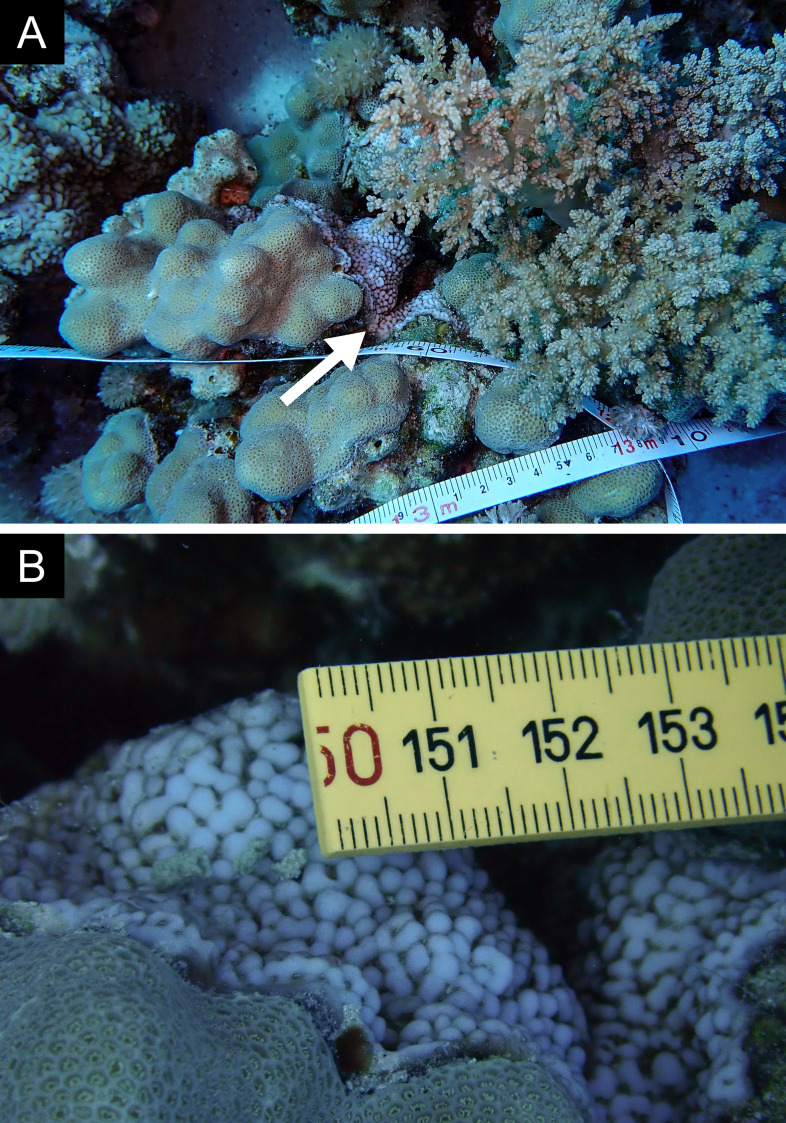
The growth form and appearance of *Montiporadanae* (bleached) in the reef are shown in (**A**), while (**B**) provides a close-up of the colony. Photo credits: Theres Koch.

**Figure 9.**
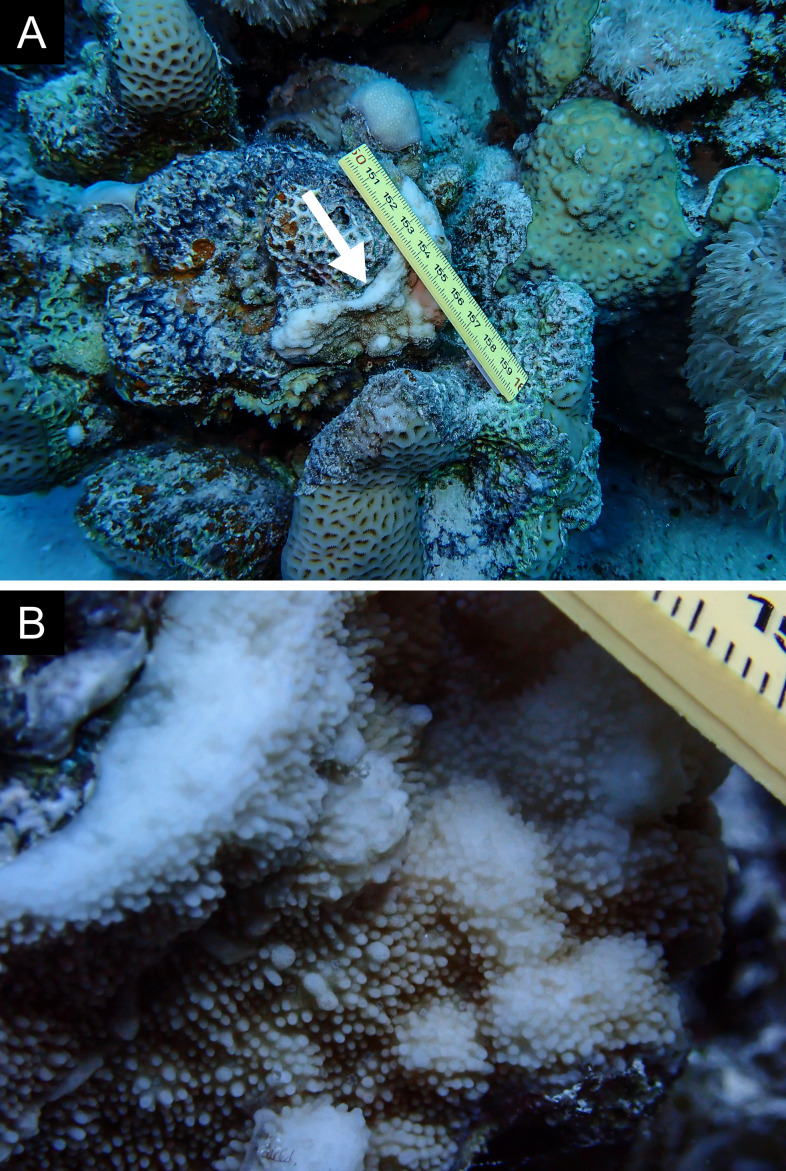
The growth form and appearance of *Montiporaefflorescens* (bleached) in the reef are shown in (**A**), while (**B**) provides a close-up of the colony. Photo credits: Theres Koch.

**Figure 10.**
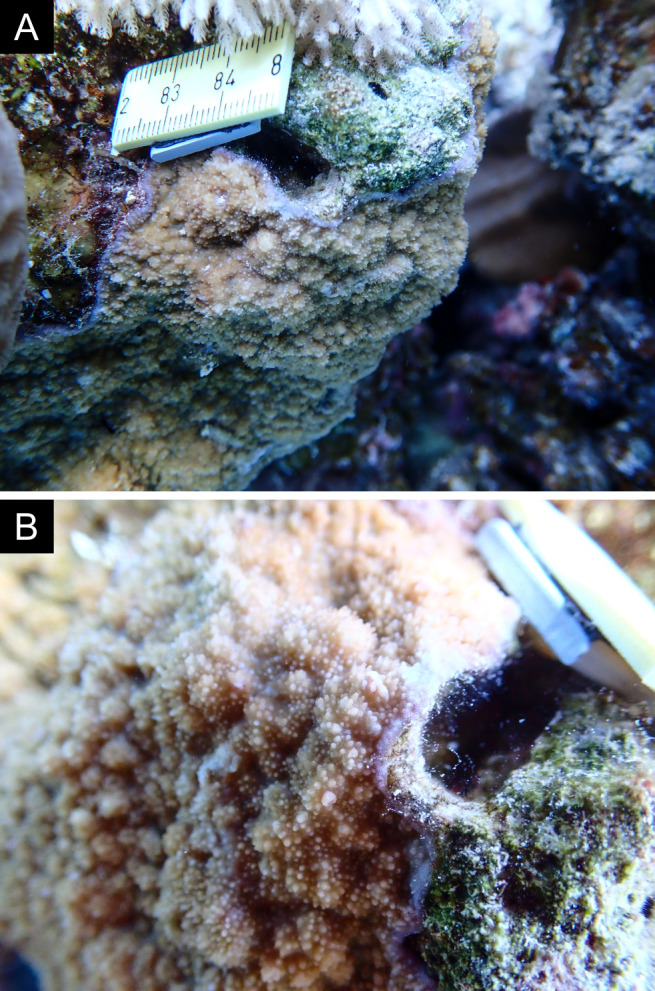
The growth form and appearance of Montiporacf.grisea in the reef are shown in (**A**), while (**B**) provides a close-up of the colony. Photo credits: Joseph Wallace Daurella.

**Figure 11.**
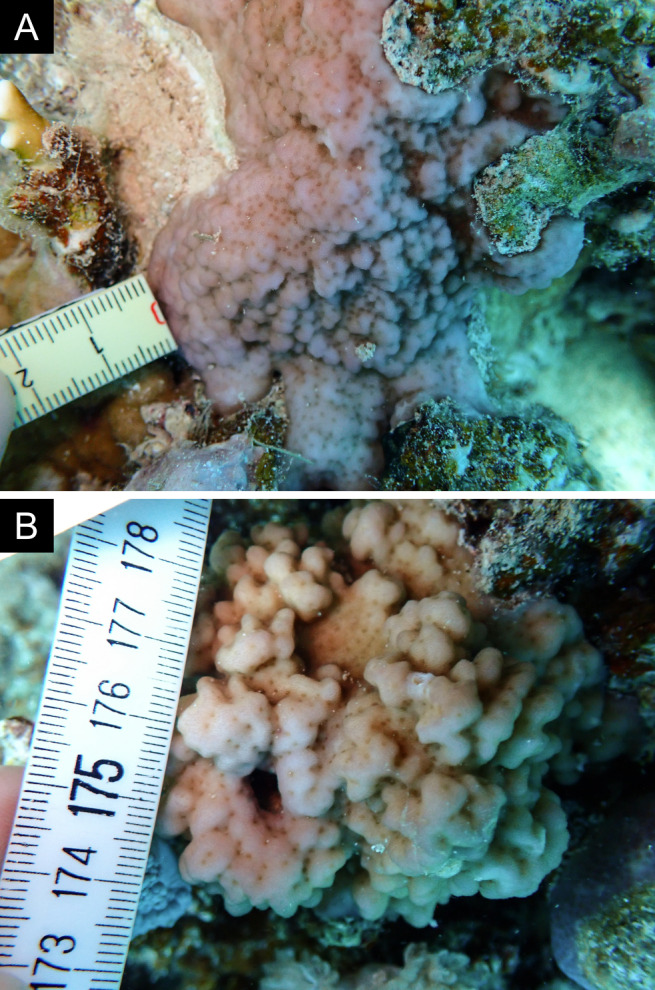
The growth form and appearance of *Montiporamaeandrina* in the reef are shown in (**A**), while (**B**) provides a close-up of another colony. Photo credits: Victor Sebastian Scharnhorst.

**Figure 13.**
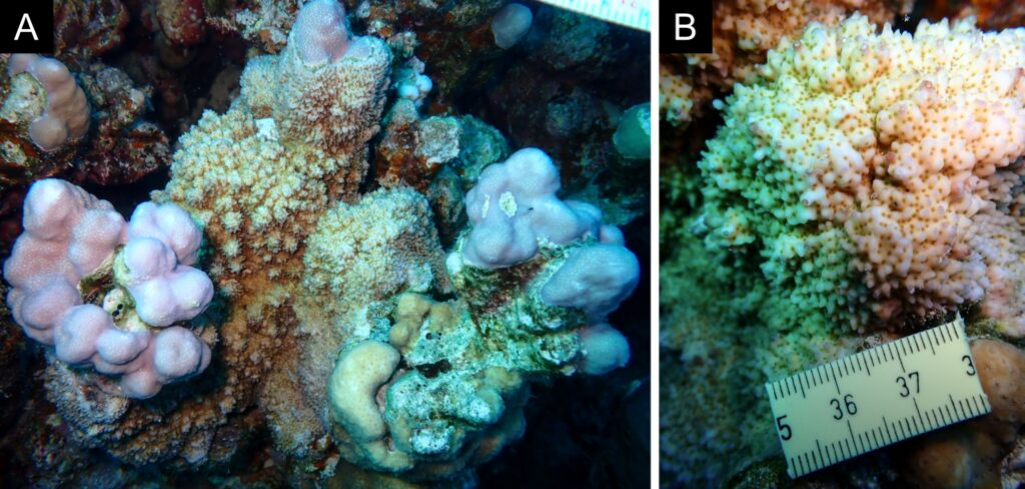
The growth form and appearance of *Montiporatuberculosa* in the reef are shown in (**A**), while (**B**) provides a close-up of the colony. Photo credits: Lewis Alan Jones.

**Figure 14.**
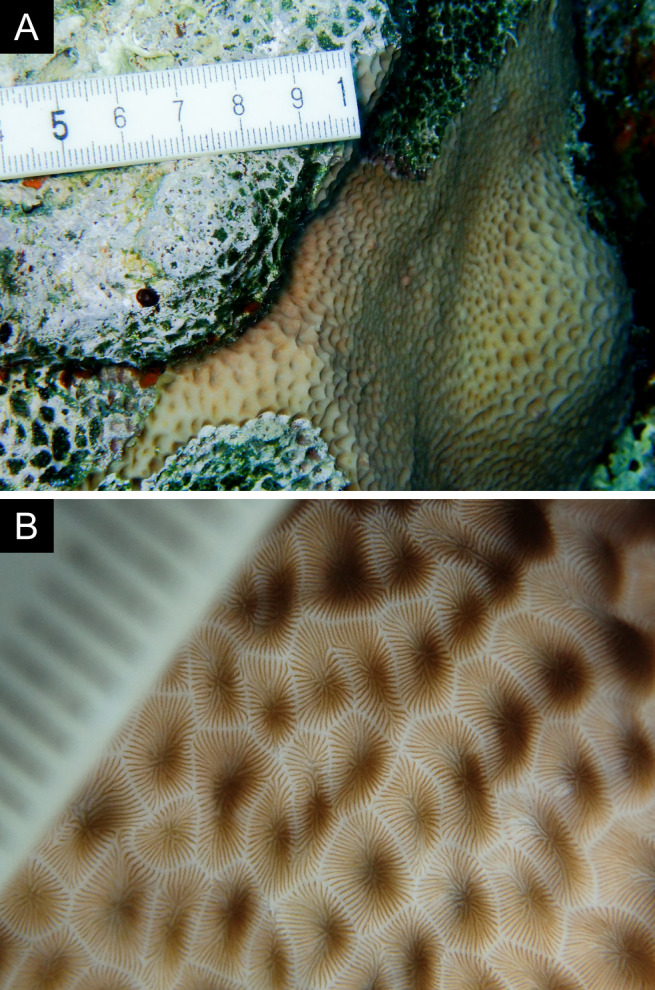
The growth form and appearance of *Gardineroserisplanulata* in the reef are shown in (**A**), while (**B**) provides a close-up of the colony. Photo credits: Theres Koch.

**Figure 15.**
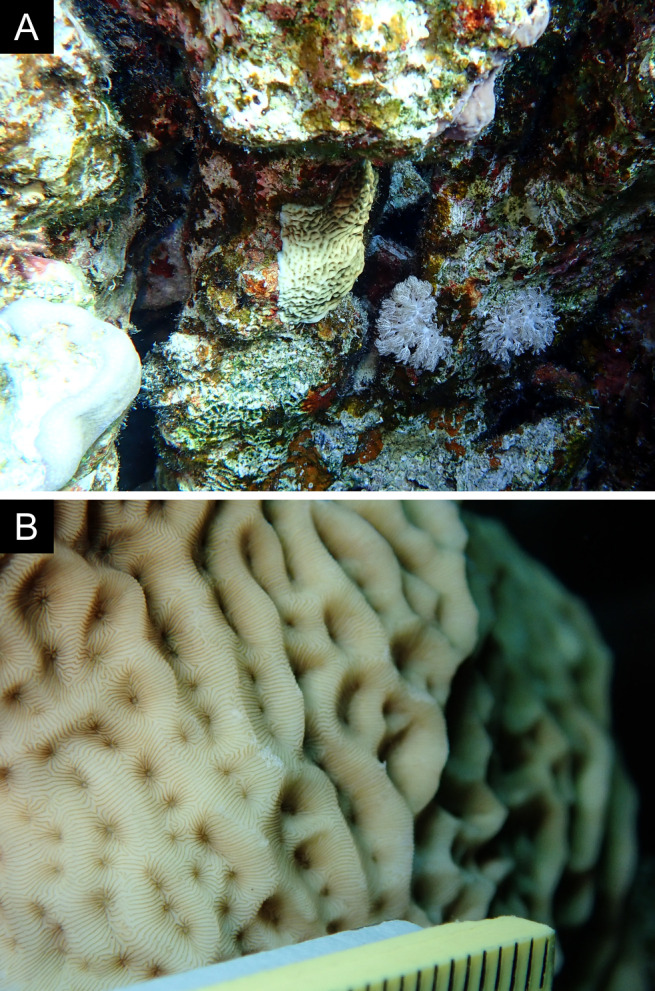
The growth form and appearance of *Leptoserismycetoseroides* in the reef are shown in (**A**), while (**B**) provides a close-up of the colony. Photo credits: Theres Koch.

**Figure 16.**
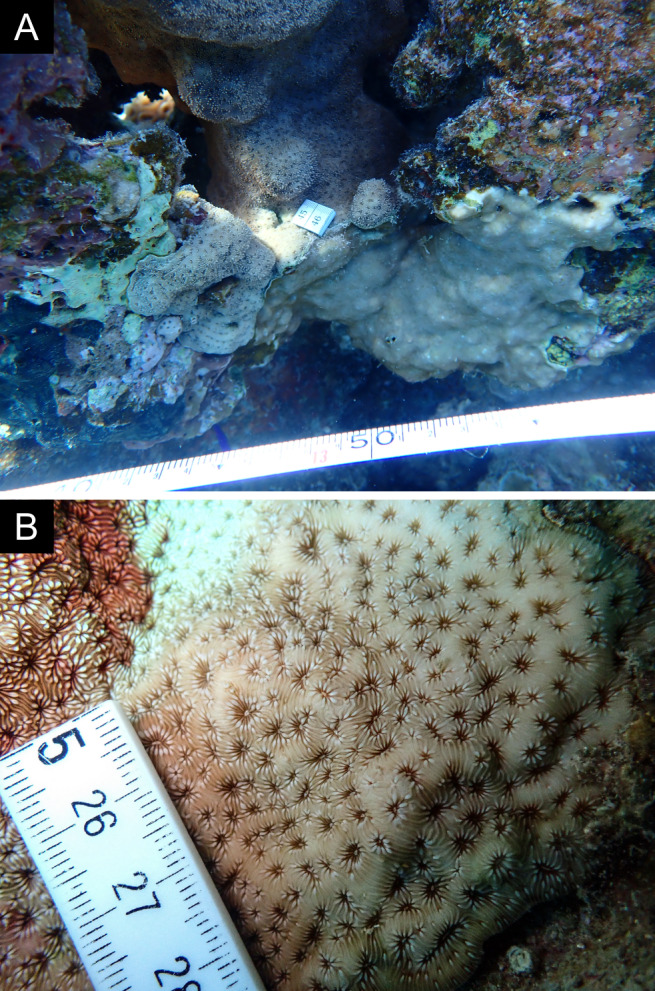
The growth form and appearance of *Pavonadiffluens* in the reef are shown in (**A**), while (**B**) provides a close-up of the colony. Photo credits: (**A**) Lewis Alan Jones; (**B**) Victor Sebastian Scharnhorst.

**Figure 18.**
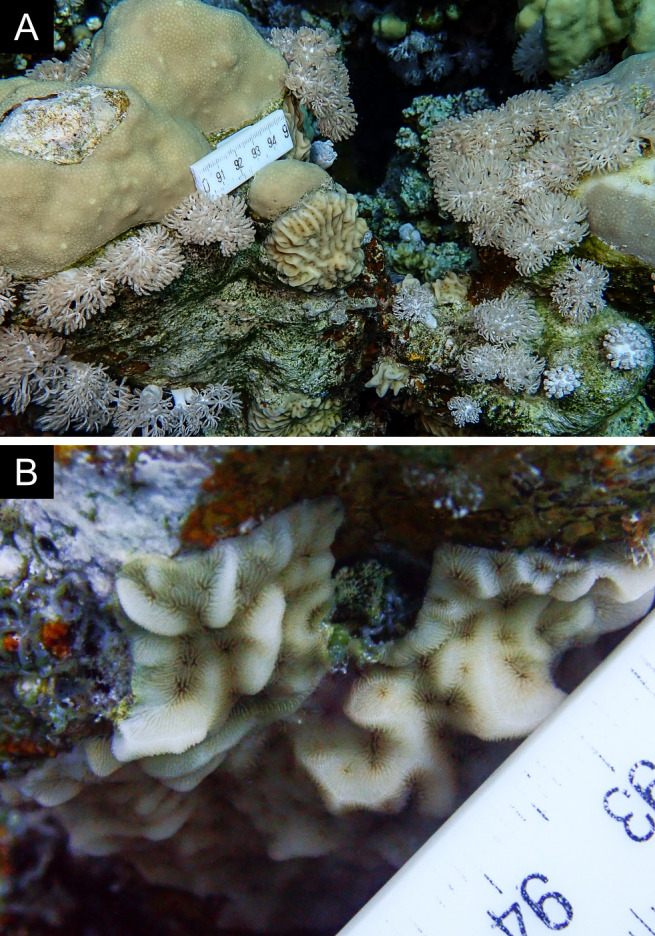
The growth form and appearance of *Pavonavarians* in the reef are shown in (**A**), while (**B**) provides a close-up of another colony. Photo credits: Theres Koch.

**Figure 20.**
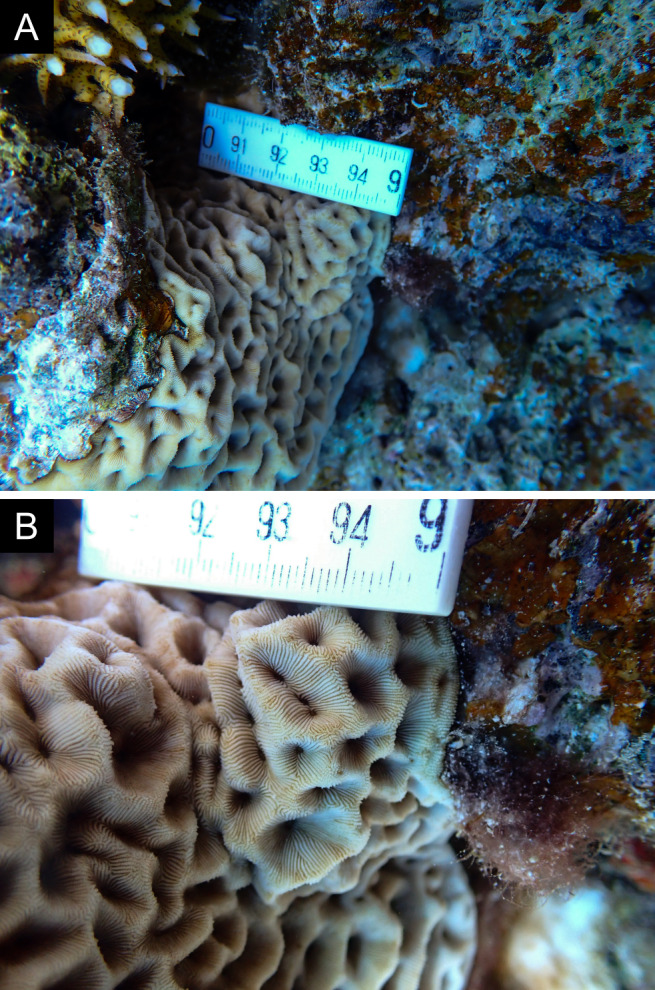
The growth form and appearance of *Coscinaraeamonile* in the reef are shown in (**A**), while (**B**) provides a close-up of the colony. Photo credits: Joseph Wallace Daurella.

**Figure 21.**
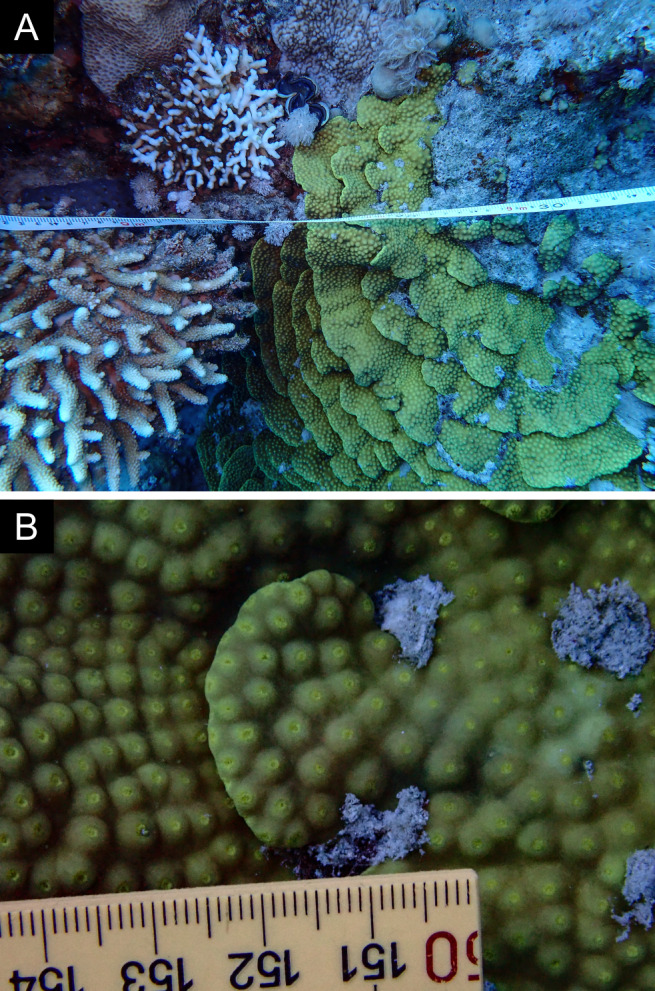
The growth form and appearance of *Turbinariamesenterina* in the reef are shown in (**A**), while (**B**) provides a close-up of the colony. Photo credits: Theres Koch.

**Figure 22.**
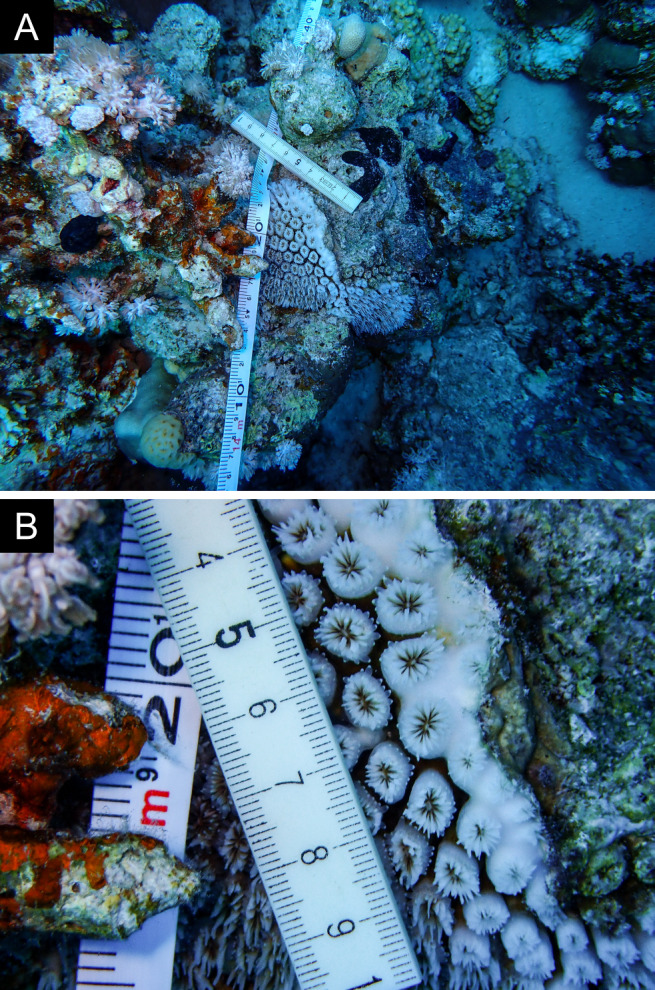
The growth form and appearance of *Galaxeafascicularis* (bleached) in the reef are shown in (**A**), while (**B**) provides a close-up of the colony. Photo credits: Theres Koch.

**Figure 23.**
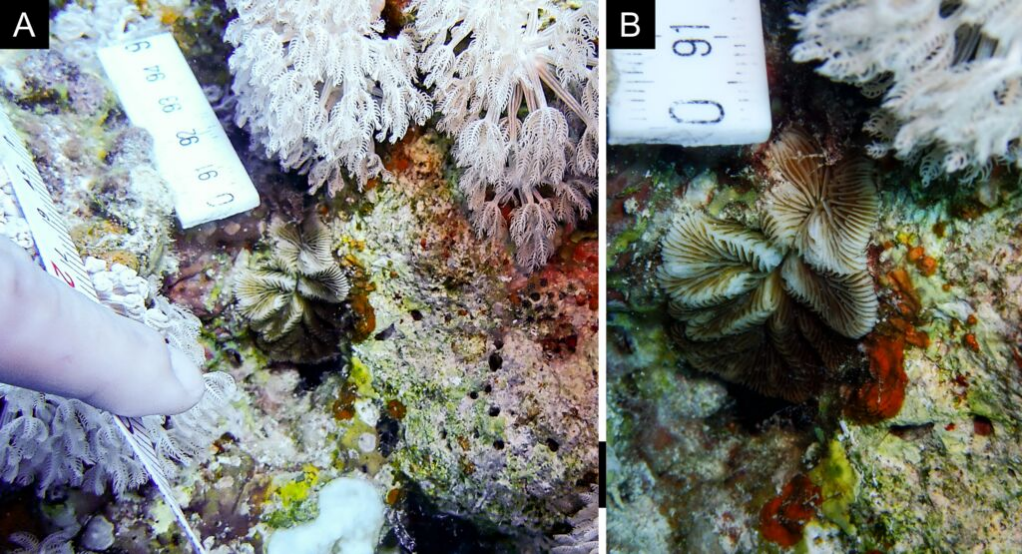
The growth form and appearance of *Gyrosmiliainterrupta* in the reef are shown in (**A**), while (**B**) provides a close-up of the colony. Photo credits: Theres Koch.

**Figure 25.**
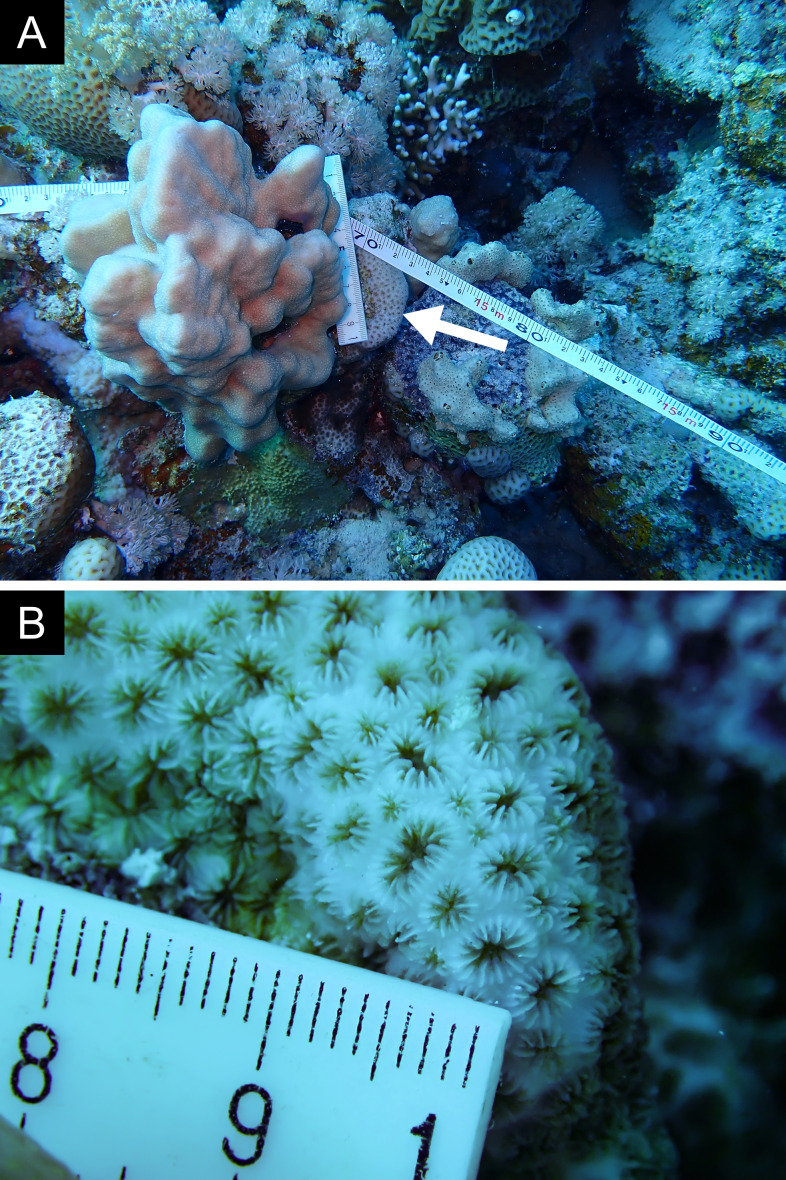
The growth form and appearance of *Leptastreabottae* (bleached) in the reef are shown in (**A**), with an arrow indicating the colony while (**B**) provides a close-up of the colony. Photo credits: Theres Koch.

**Figure 26.**
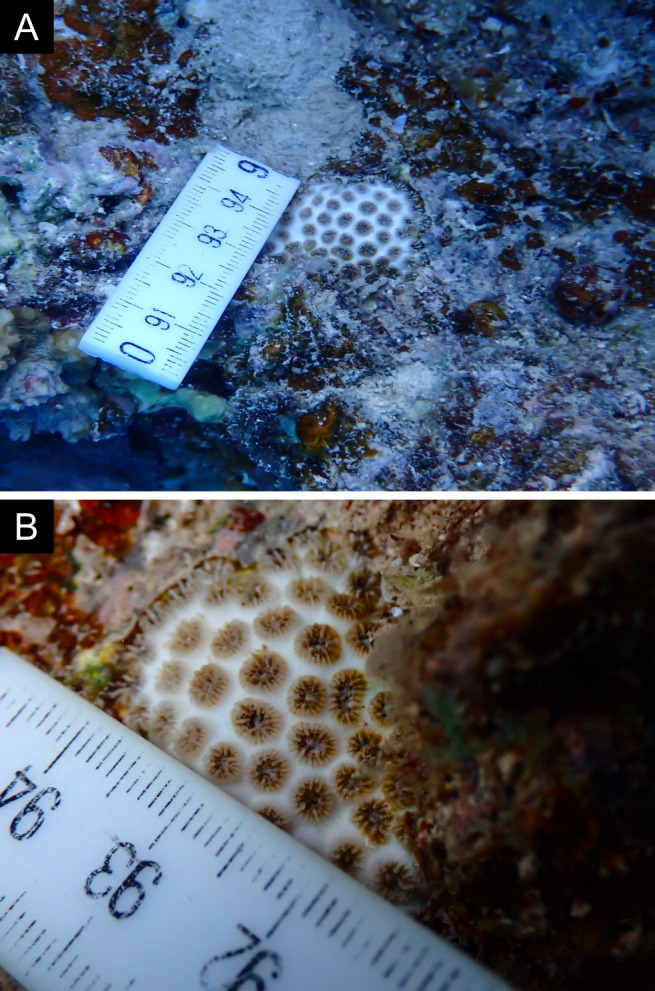
The growth form and appearance of *Leptastreainaequalis* (bleached) in the reef are shown in (**A**), while (**B**) provides a close-up of the colony. Photo credits: Lewis Alan Jones.

**Figure 27.**
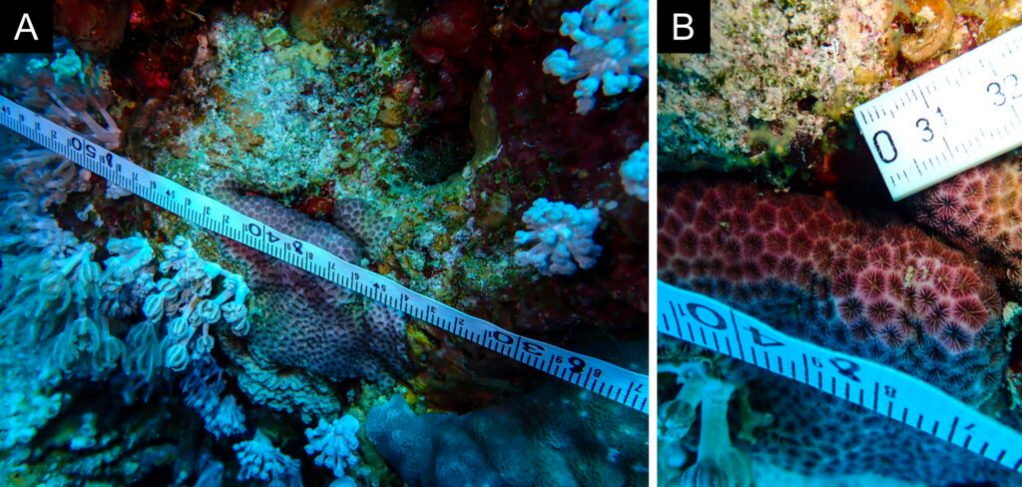
The growth form and appearance of *Leptastreatransversa* in the reef are shown in (**A**), while (**B**) provides a close-up of the colony. Photo credits: Victor Sebastian Scharnhorst.

**Figure 28.**
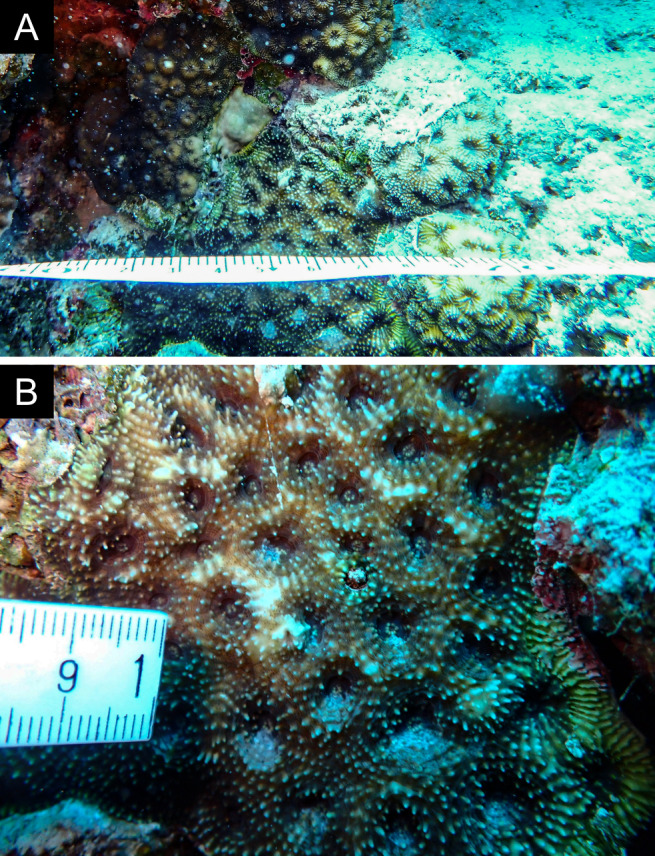
The growth form and appearance of *Acanthastreahemprichii* in the reef are shown in (**A**), while (**B**) provides a close-up of the colony. Photo credits: Victor Sebastian Scharnhorst.

**Figure 32.**
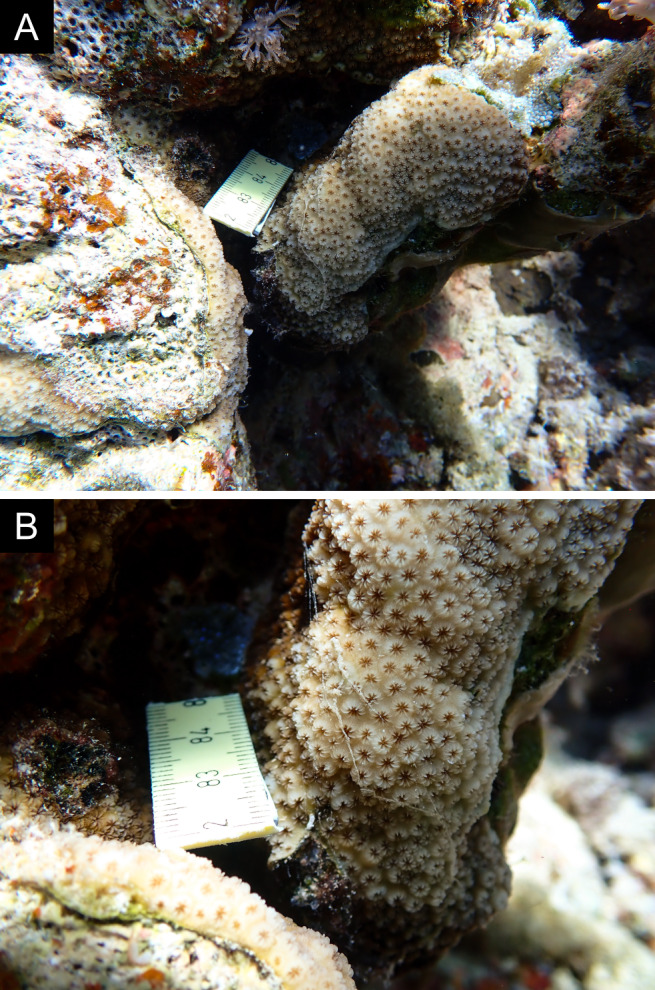
The growth form and appearance of *Cyphastreachalcidicum* in the reef are shown in (**A**), while (**B**) provides a close-up of the colony. Photo credits: Joseph Wallace Daurella.

**Figure 33.**
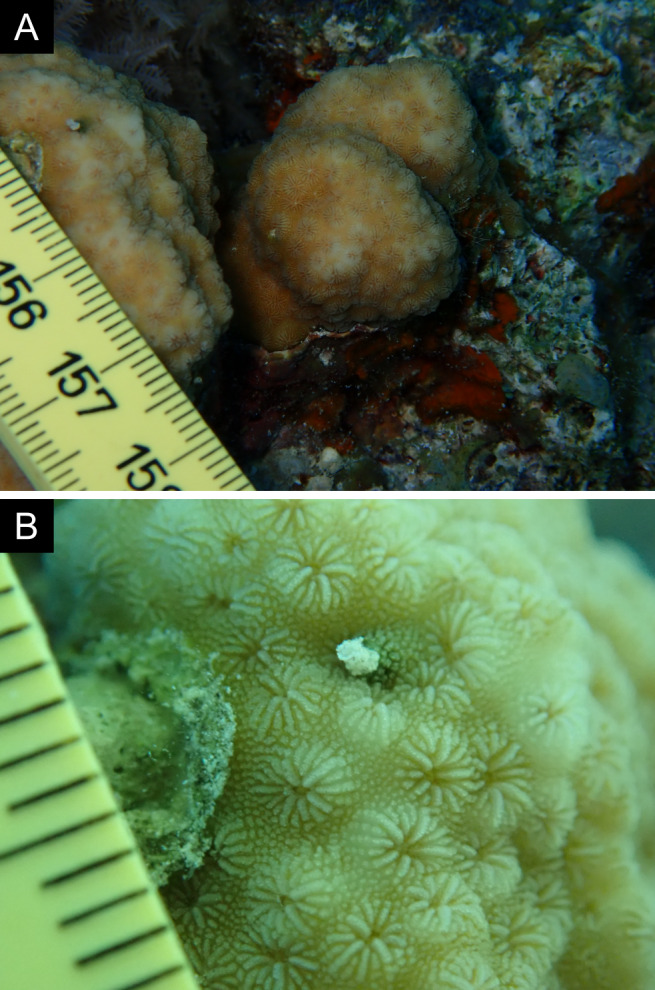
The growth form and appearance of *Cyphastreakausti* in the reef are shown in (**A**), while (**B**) provides a close-up of the colony. Photo credits: Theres Koch.

**Figure 34.**
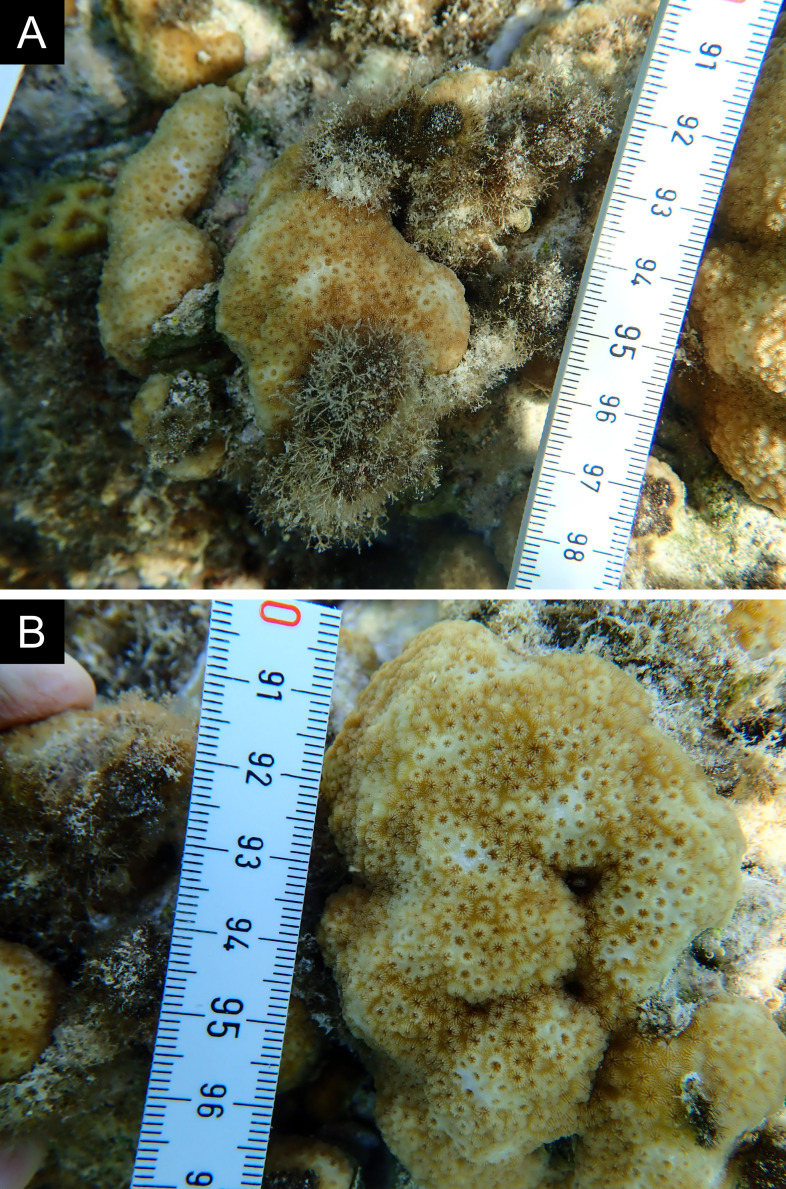
The growth form and appearance of *Cyphastreamagna* in the reef are shown in (**A**), while (**B**) provides a close-up of the colony. Photo credits: Antonia Auer, Theda Schöchtner, Gözde Özer.

**Figure 35.**
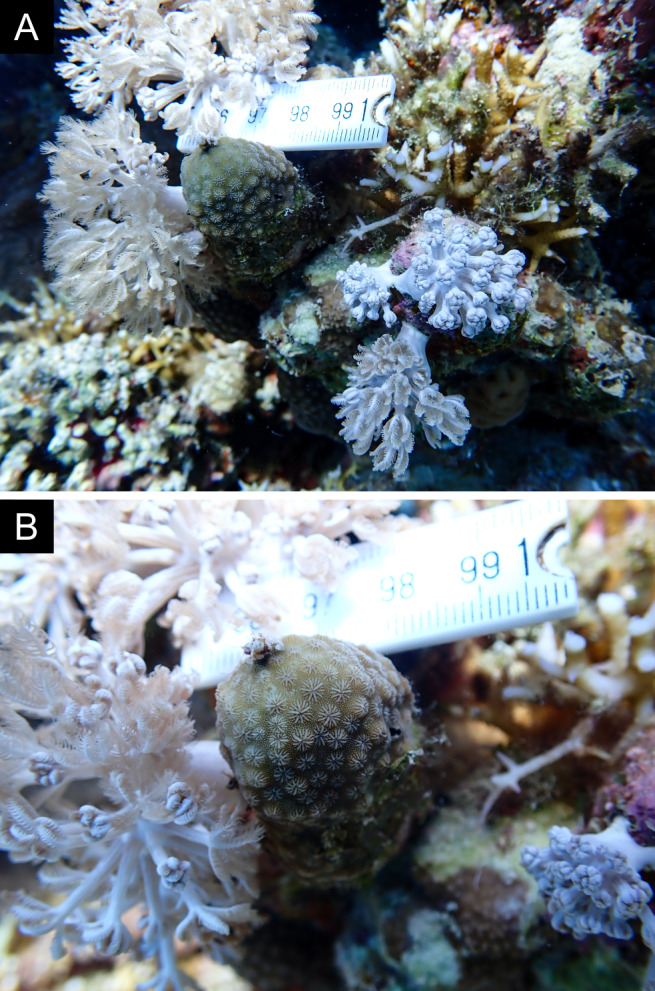
The growth form and appearance of *Cyphastreamicrophthalma* in the reef are shown in (**A**), while (**B**) provides a close-up of the colony. Photo credits: Joseph Wallace Daurella.

**Figure 37.**
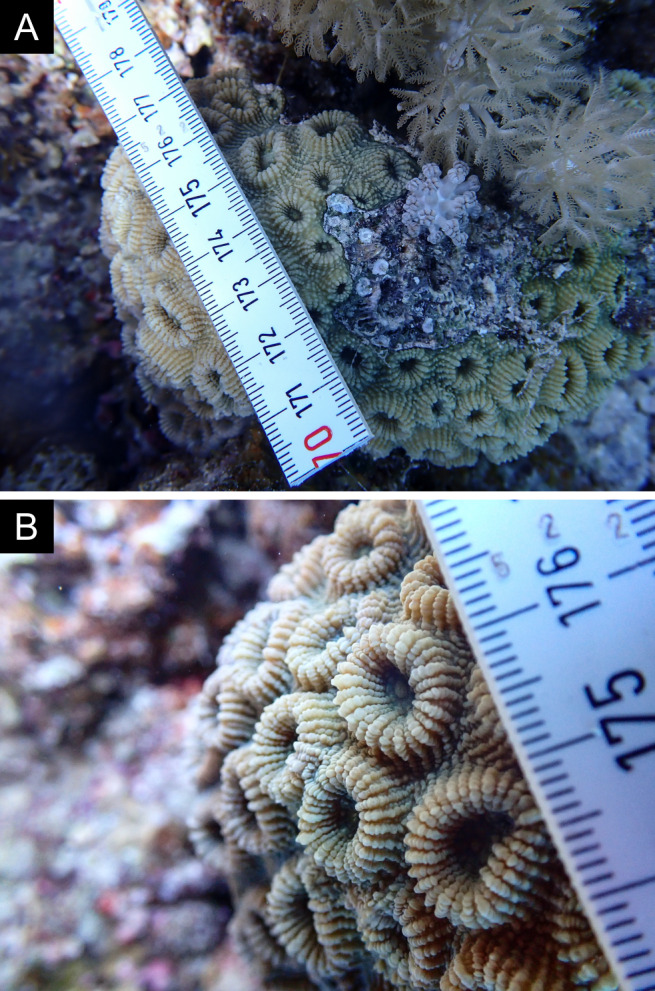
The growth form and appearance of *Dipsastraeadanai* in the reef are shown in (**A**), while (**B**) provides a close-up of the colony. Photo credits: Joseph Wallace Daurella.

**Figure 38.**
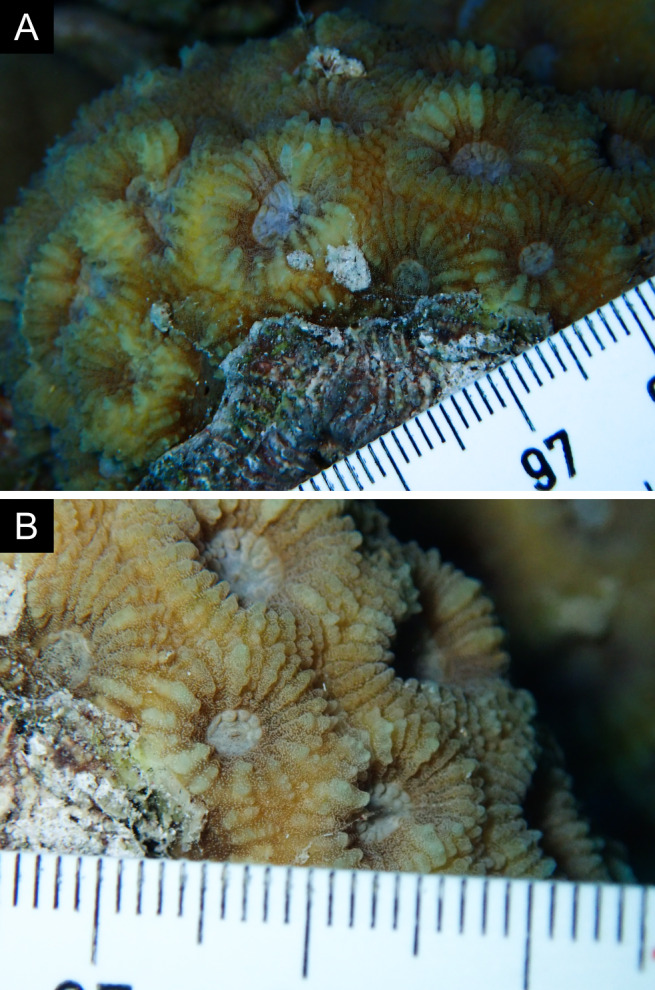
The growth form and appearance of *Dipsastraeafaviaformis* in the reef are shown in (**A**), while (**B**) provides a close-up of the colony. Photo credits: Theres Koch.

**Figure 39.**
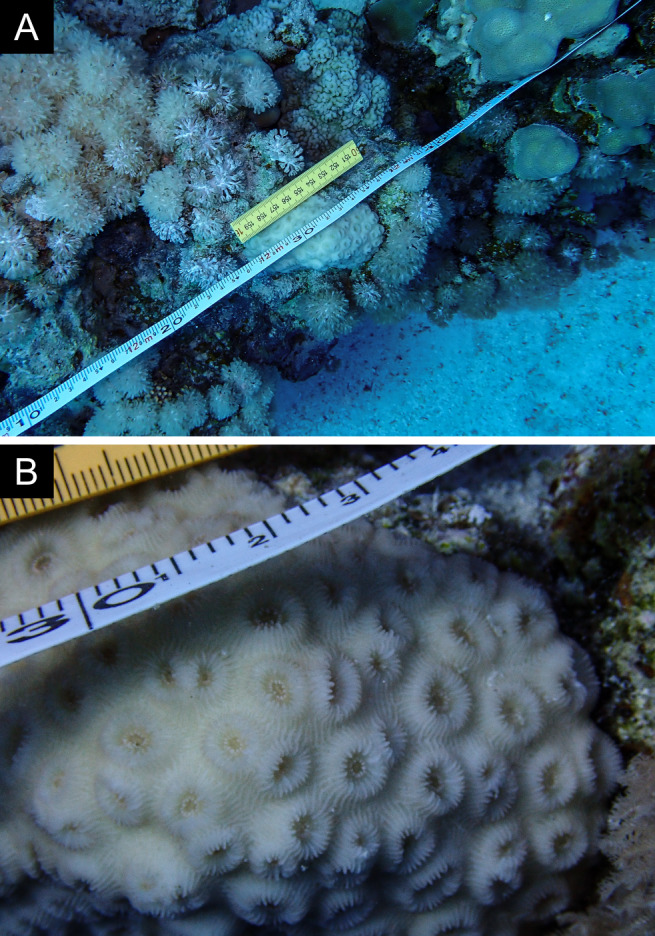
The growth form and appearance of *Dipsastraealaxa* (bleached) in the reef are shown in (**A**), while (**B**) provides a close-up of the colony. Photo credits: Theres Koch.

**Figure 40.**
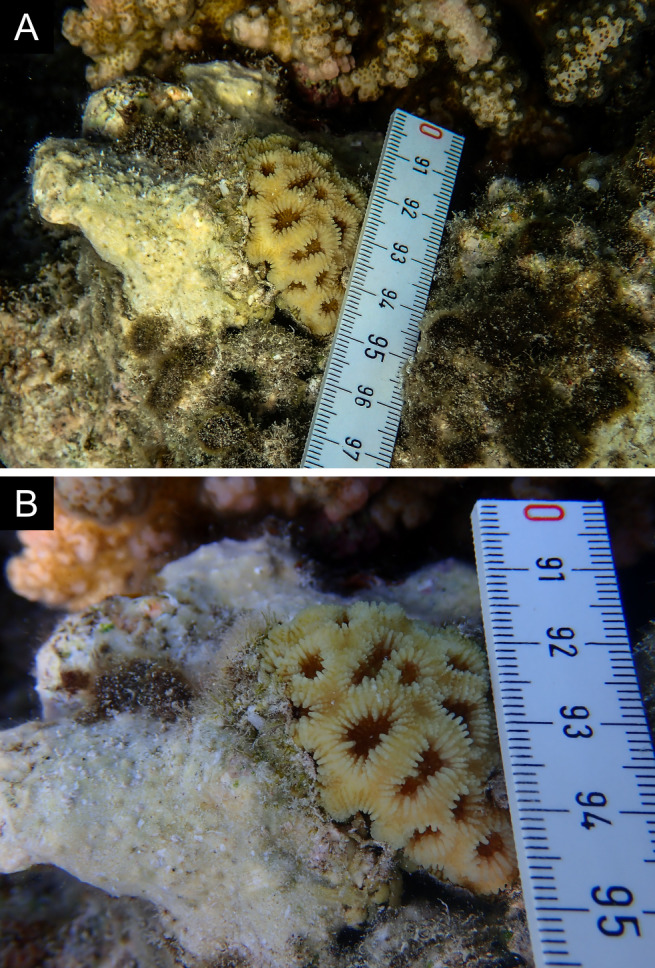
The growth form and appearance of *Dipsastraeamatthaii* in the reef are shown in (**A**), while (**B**) provides a close-up of the colony. Photo credits: Antonia Auer, Theda Schöchtner, Gözde Özer.

**Figure 41.**
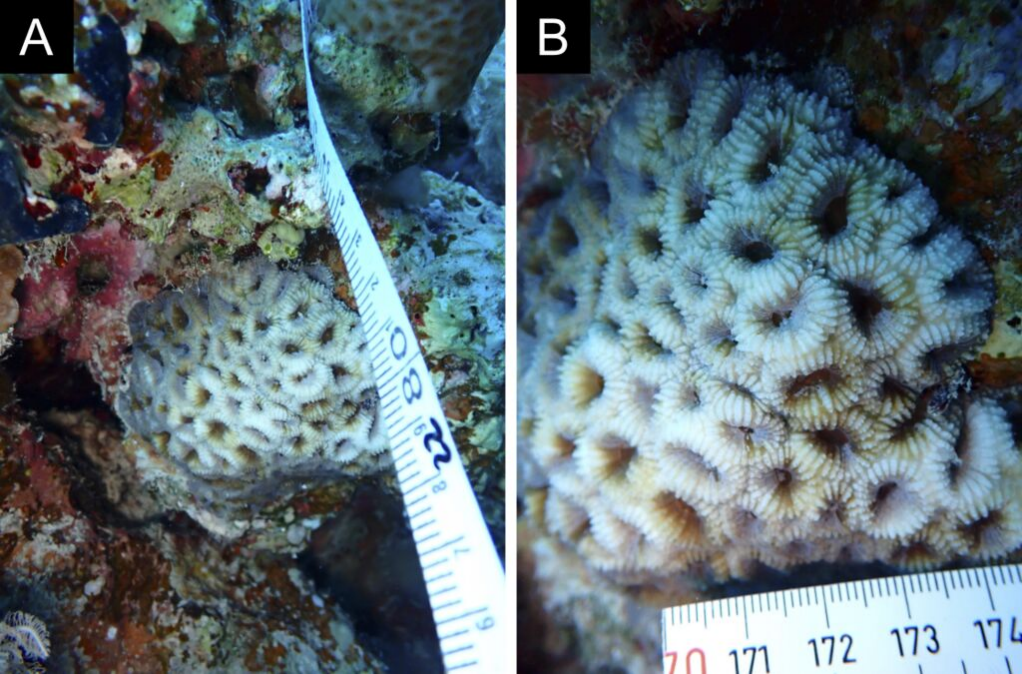
The growth form and appearance of *Dipsastraeapallida* (bleached) in the reef are shown in (**A**), while (**B**) provides a close-up of the colony. Photo credits: Victor Sebastian Scharnhorst.

**Figure 42.**
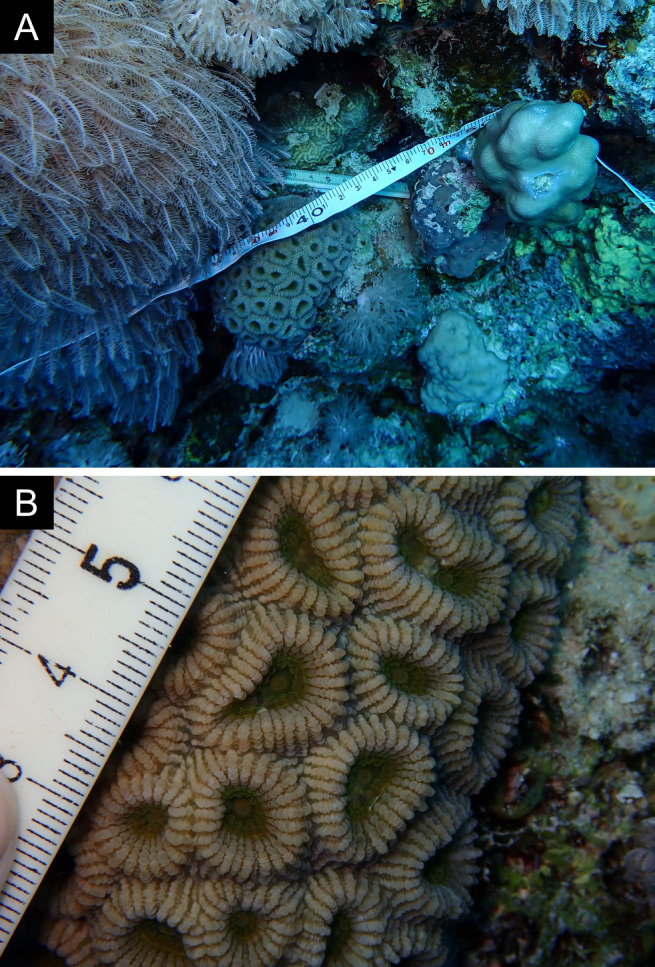
The growth form and appearance of *Dipsastraeaspeciosa* in the reef are shown in (**A**), while (**B**) provides a close-up of the colony. Photo credits: Theres Koch.

**Figure 43.**
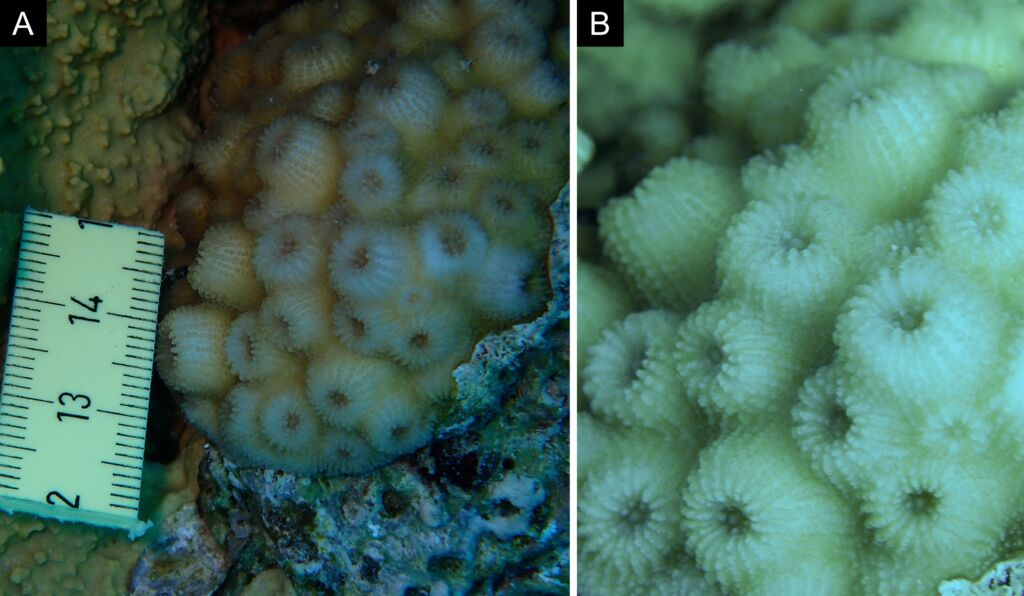
The growth form and appearance of *Echinoporaforskaliana* in the reef are shown in (**A**), while (**B**) provides a close-up of the colony. Photo credits: Theres Koch.

**Figure 44.**
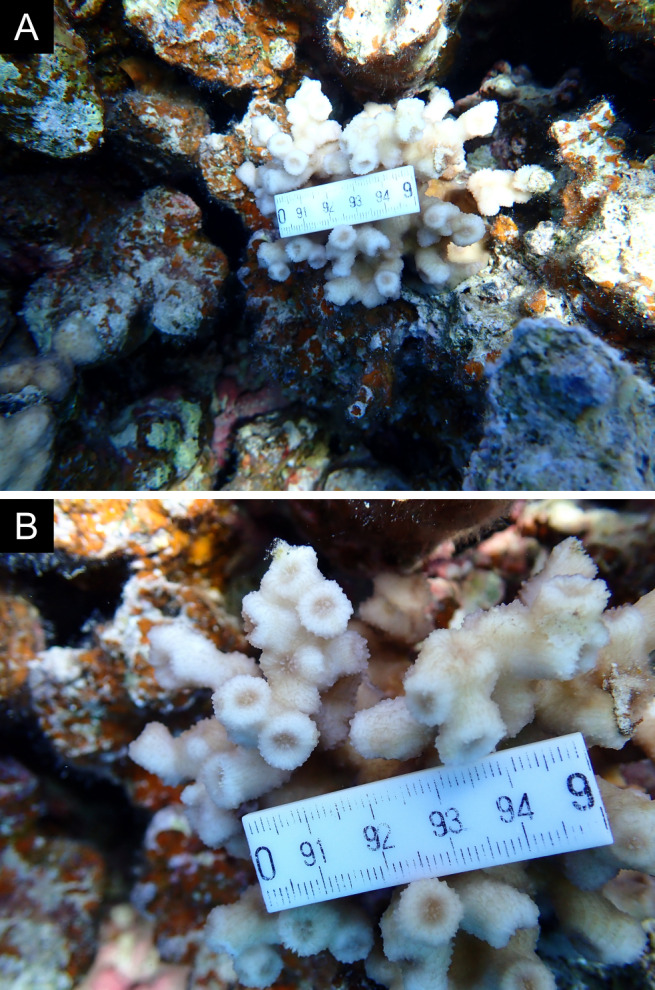
The growth form and appearance of *Echinoporafruticulosa* (bleached) in the reef are shown in (**A**), while (**B**) provides a close-up of the colony. Photo credits: Joseph Wallace Daurella.

**Figure 45.**
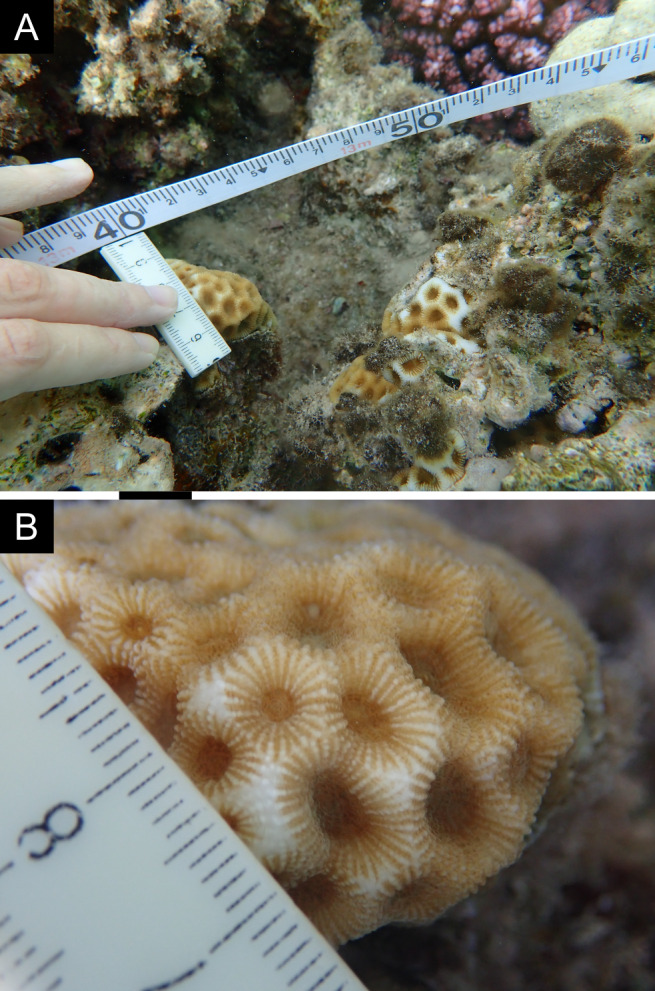
The growth form and appearance of *Favitesabdita* in the reef are shown in (**A**), while (**B**) provides a close-up of the colony. Photo credits: Antonia Auer, Theda Schöchtner, Gözde Özer.

**Figure 46.**
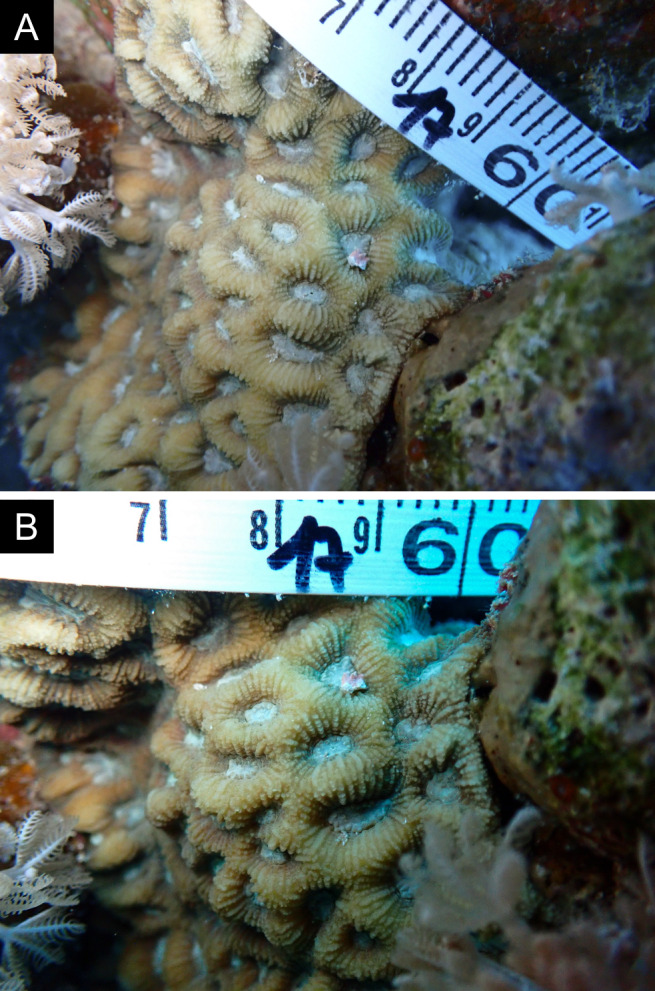
The growth form and appearance of Favitescf.complanata in the reef are shown in (**A**), while (**B**) provides a close-up of the colony. Photo credits: Victor Sebastian Scharnhorst.

**Figure 47.**
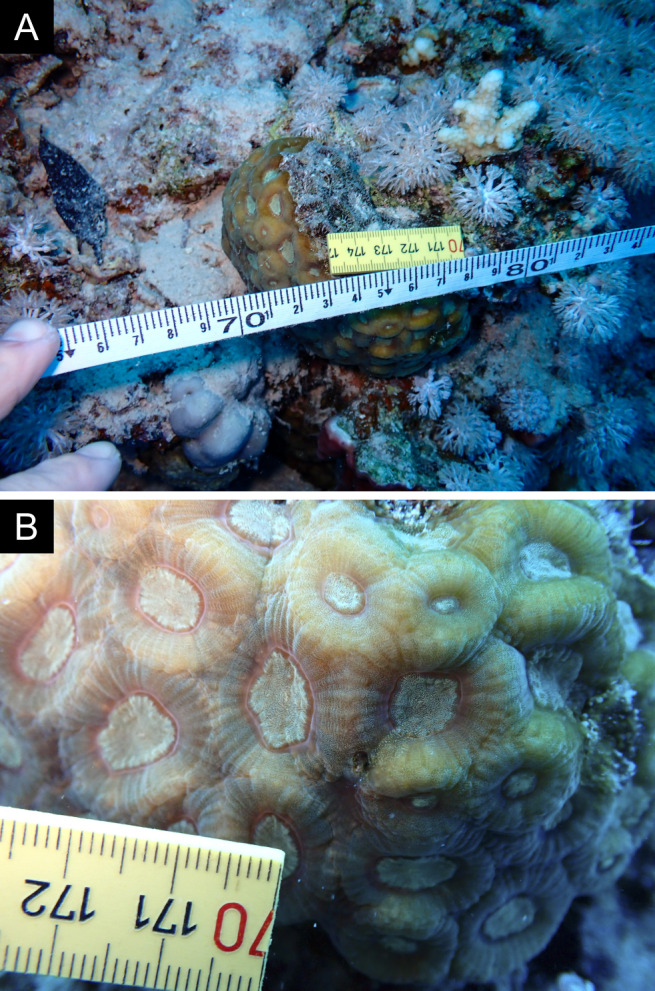
The growth form and appearance of *Favitesrotundata* in the reef are shown in (**A**), while (**B**) provides a close-up of the colony. Photo credits: Victor Sebastian Scharnhorst.

**Figure 48.**
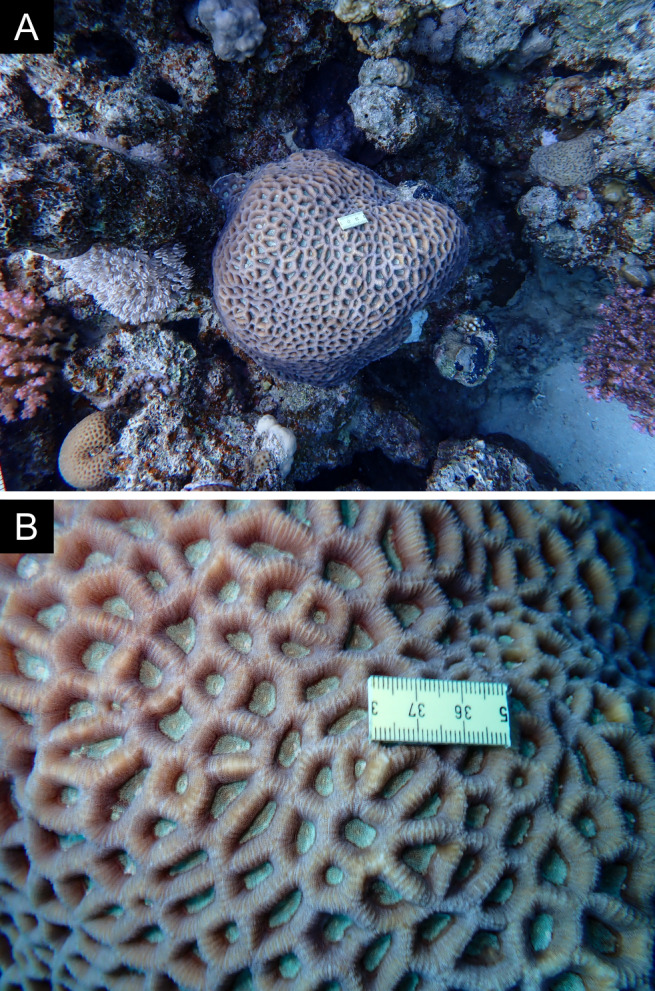
The growth form and appearance of *Favitesvasta* in the reef are shown in (**A**), while (**B**) provides a close-up of the colony. Photo credits: Lewis Alan Jones.

**Figure 49.**
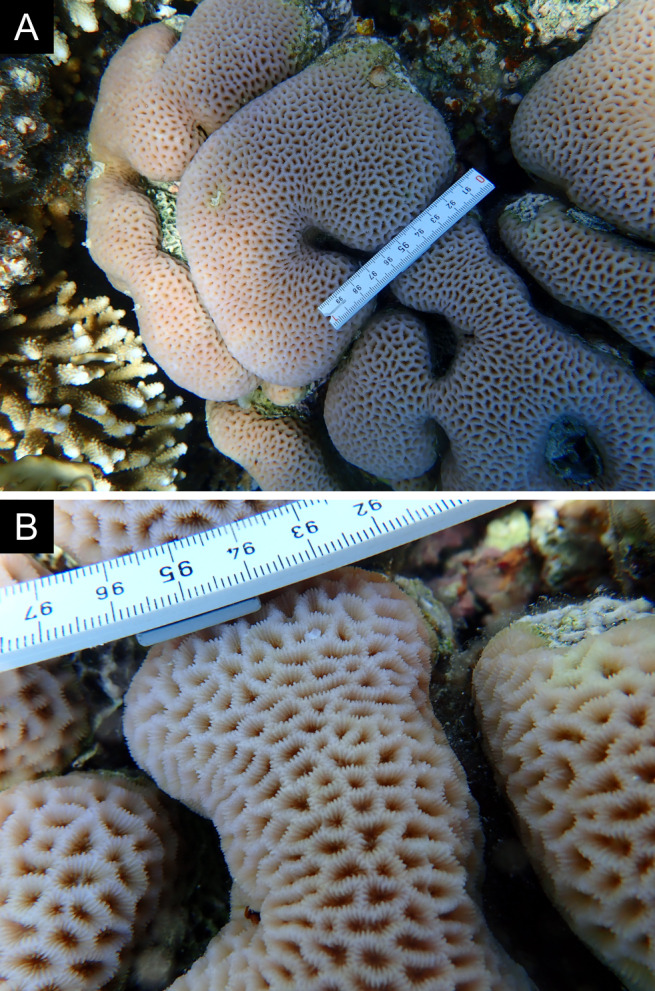
The growth form and appearance of *Goniastreaedwardsi* in the reef are shown in (**A**), while (**B**) provides a close-up of the colony. Photo credits: Antonia Auer, Theda Schöchtner, Gözde Özer.

**Figure 50.**
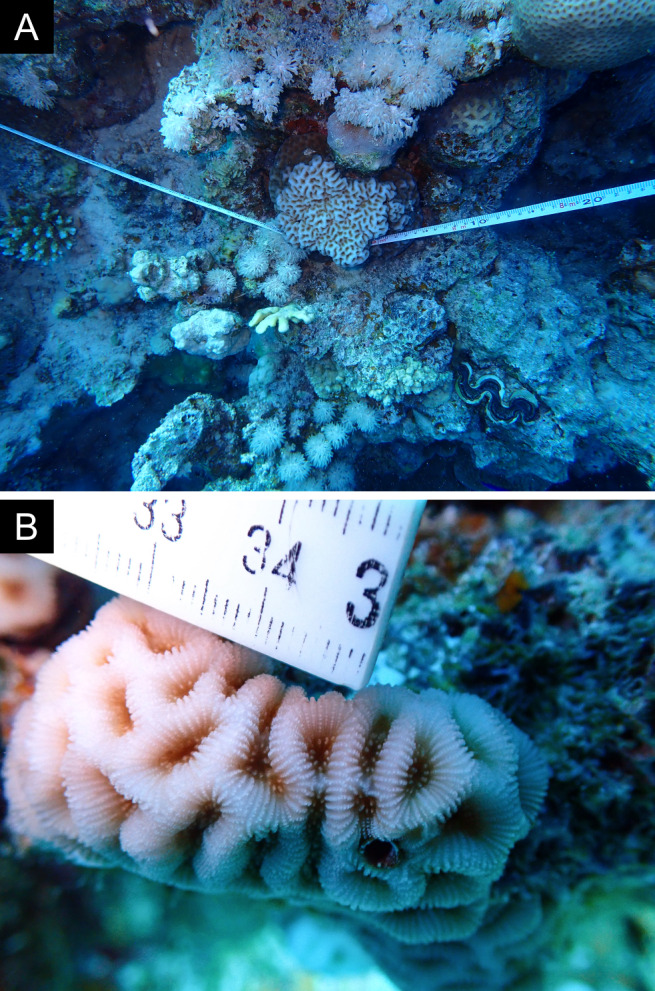
The growth form and appearance of *Goniastreapectinata* (bleached) in the reef are shown in (**A**), while (**B**) provides a close-up of another colony. Photo credits: (**A**) Theres Koch; (**B**) Victor Sebastian Scharnhorst.

**Figure 51.**
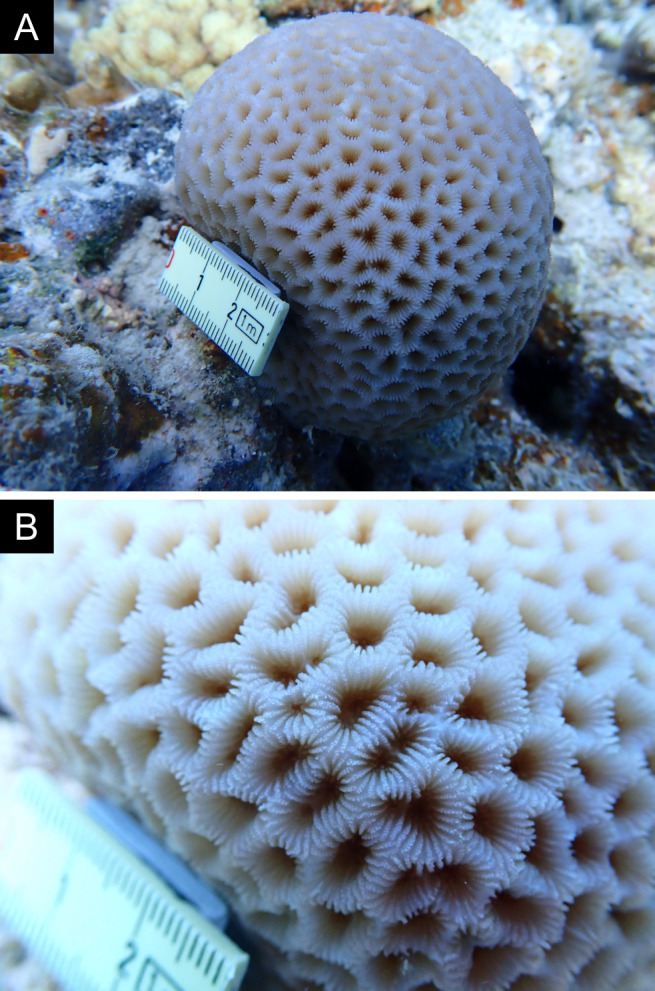
The growth form and appearance of *Goniastrearetiformis* (bleached) in the reef are shown in (**A**), while (**B**) provides a close-up of the colony. Photo credits: Lewis Alan Jones.

**Figure 52.**
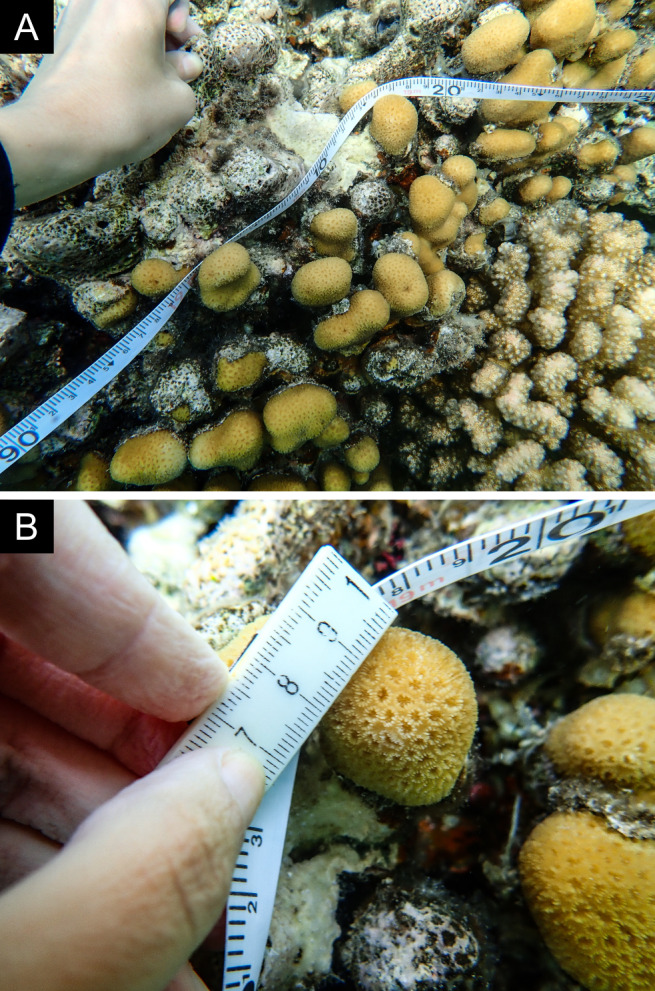
The growth form and appearance of *Goniastreastelligera* in the reef are shown in (**A**), while (**B**) provides a close-up of the colony. Photo credits: Antonia Auer, Theda Schöchtner, Gözde Özer.

**Figure 53.**
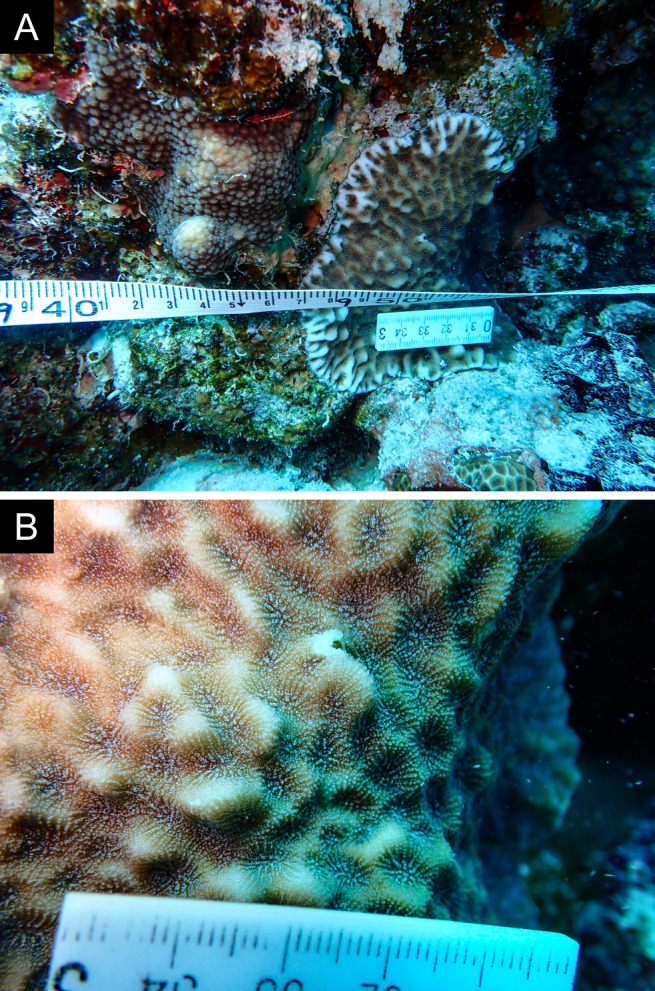
The growth form and appearance of *Merulinascheeri* in the reef are shown in (**A**), while (**B**) provides a close-up of the colony. Photo credits: Victor Sebastian Scharnhorst.

**Figure 54.**
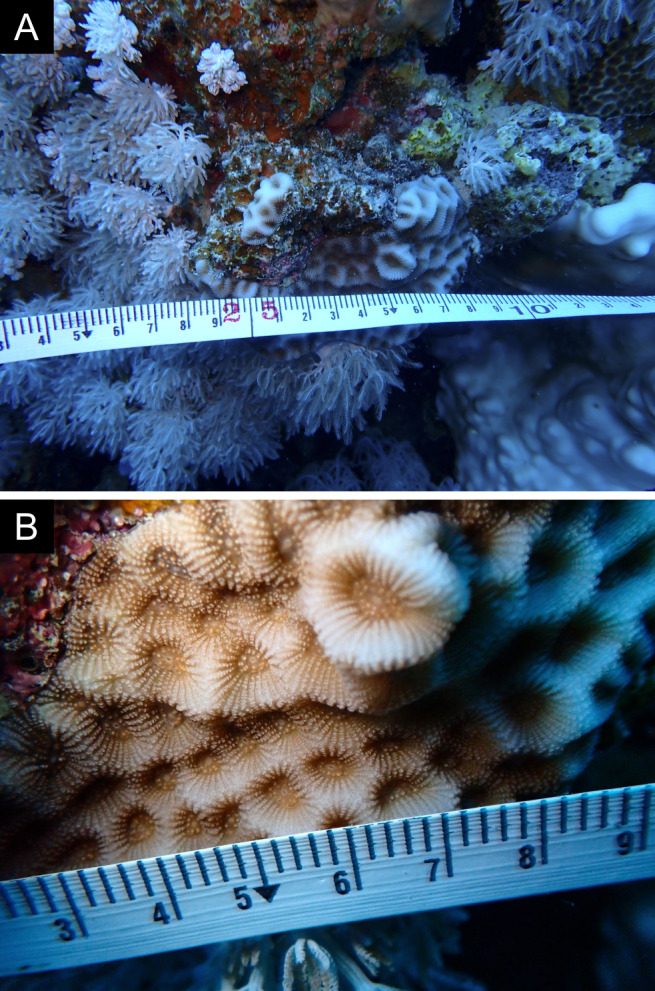
The growth form and appearance of *Paramontastraeaperesi* (bleached) in the reef are shown in (**A**), while (**B**) provides a close-up of the colony. Photo credits: Victor Sebastian Scharnhorst.

**Figure 55.**
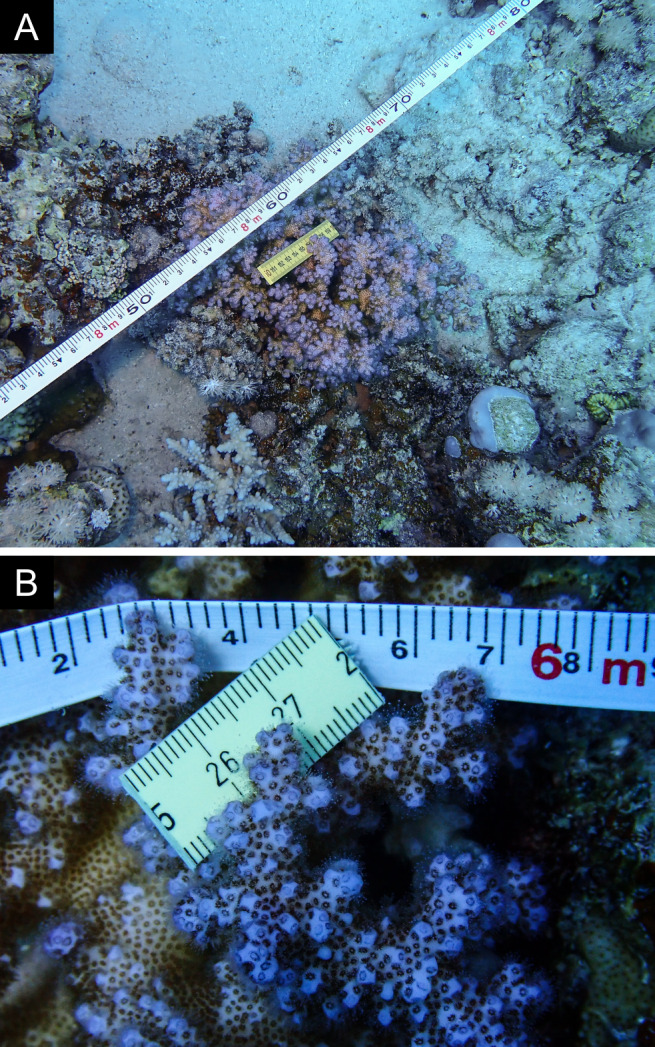
The growth form and appearance of *Pocilloporadamicornis* in the reef are shown in (**A**), while (**B**) provides a close-up of another colony. Photo credits: Theres Koch.

**Figure 57.**
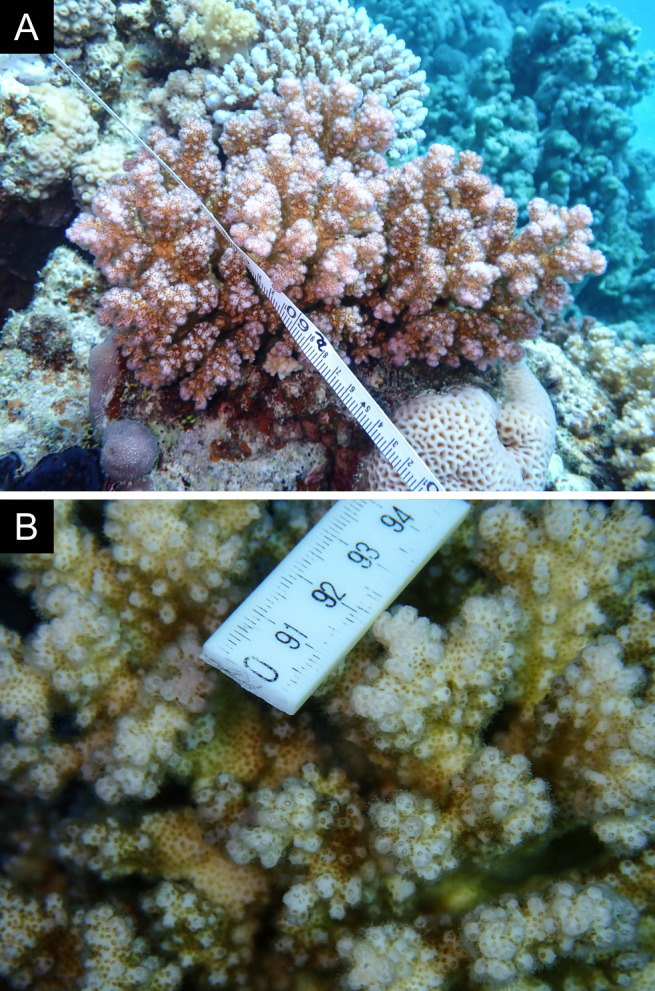
The growth form and appearance of *Pocilloporaverrucosa* in the reef are shown in (**A**), while (**B**) provides a close-up of another (bleached) colony. Photo credits: (**A**) Victor Sebastian Scharnhorst; (**B**) Theres Koch.

**Figure 58.**
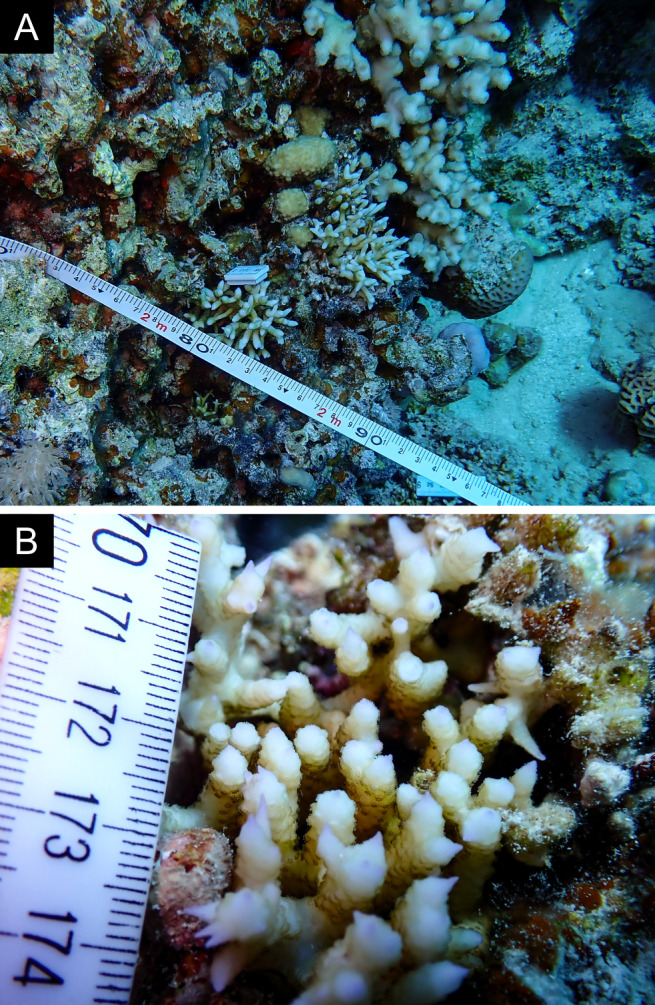
The growth form and appearance of *Seriatoporacaliendrum* (bleached) in the reef are shown in (**A**), while (**B**) provides a close-up of the colony. Photo credits: (**A**) Theres Koch; (**B**) Victor Sebastian Scharnhorst.

**Figure 59.**
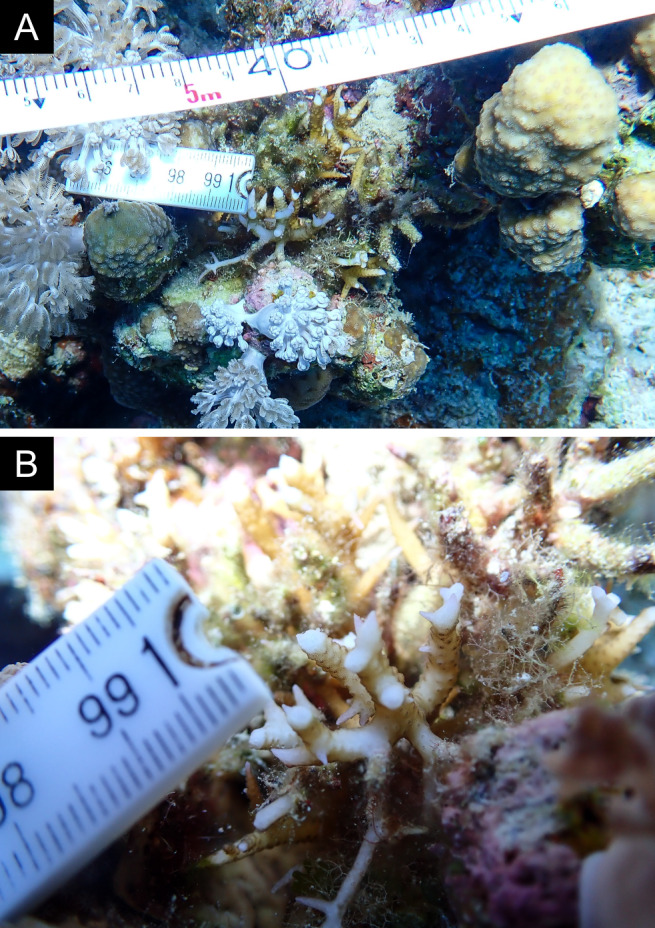
The growth form and appearance of *Seriatoporahystrix* in the reef are shown in (**A**), while (**B**) provides a close-up of the colony. Photo credits: Joseph Wallace Daurella.

**Figure 64.**
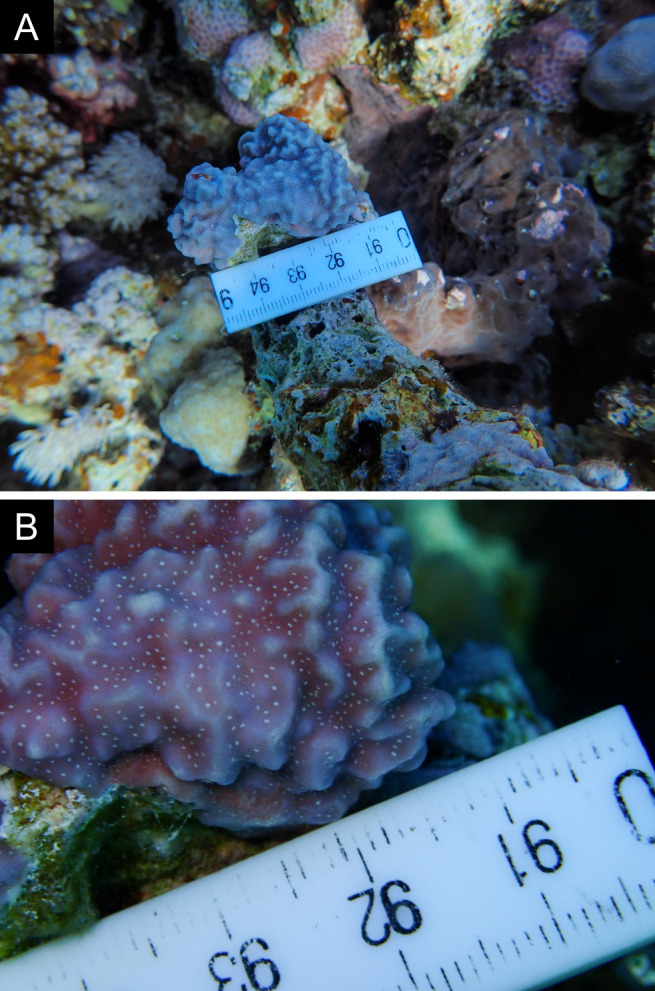
The growth form and appearance of *Poritesrus* in the reef are shown in (**A**), while (**B**) provides a close-up of the colony. Photo credits: Theres Koch.

**Figure 66.**
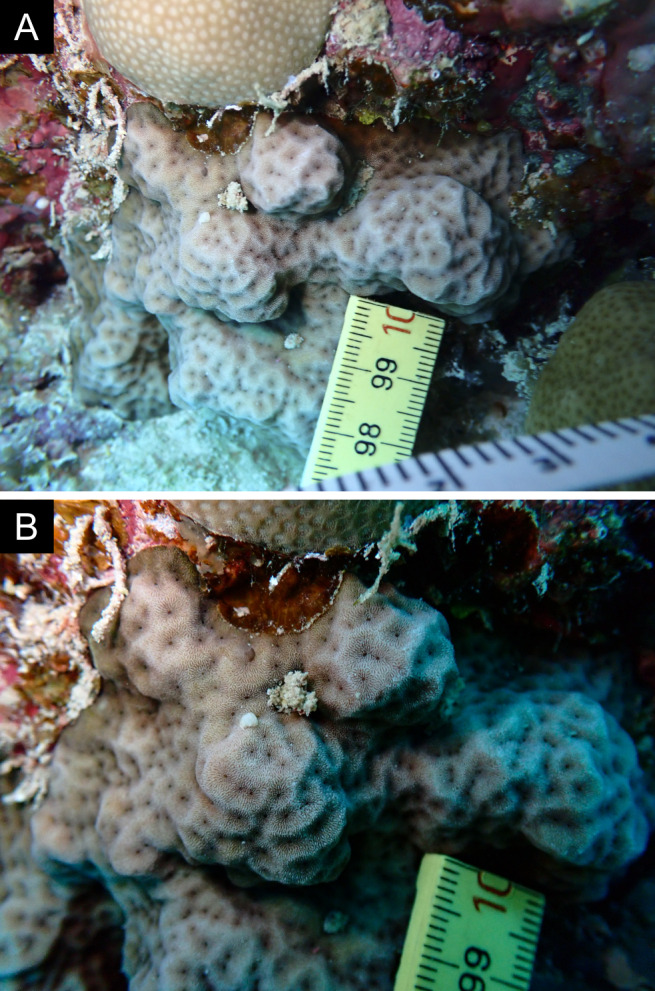
The growth form and appearance of *Psammocoraprofundacella* in the reef are shown in (**A**), while (**B**) provides a close-up of the colony. Photo credits: Victor Sebastian Scharnhorst.

**Table 1.** Life form categories and to classify coral growth forms adapted from English et al. (1994).

Categories		Code
Scleractinian Corals		
	Dead Coral	DC
	Dead Coral with Algae	DCA
	*Acropora* Coral branching	ACB
	*Acropora* Coral digitate	ACB
	Coral branching	CB
	Coral encrusting	CE
	Coral foliaceous	CF
	Coral massive	CM
	Coral mushroom	CMR
	Coral submassive	CS
Algae		
	Algal Assemblage	AA
	Coralline Algae	CA
	Macro Algae	MA
	Turf Algae	TA
Millepora		
	*Milleporadichotoma*	CME
	*Milleporaexaesa*	
	*Milleporaplatyphylla*	
	*Millepora* sp.	
Benthic Fauna		
	Mollusc	MOLL
	Other Species	OT
	Soft Coral	SC
	Sponge	SP
Substrate Types		
	Coral rubble	CR
	Rock	R
	Sand	S

**Table 2.** The observed occurrence of scleractinian coral species identified at both reef sites and across six habitats in Mangrove Bay, as indicated with an "X". Abbreviations: **reef edge** (**RE**, 1 m), **shallow reef slope** (**SR**, 4.3 m for the current-exposed site and 3.7–4.75 m for the sheltered site) and **deeper reef slope** (**DR**, 8.5–8.8 m for the current-exposed site and 7.2–9.5 m for the sheltered site).

**Family**	**Genus and Species**	**Sheltered** **Reef**			**Current-exposed** **Reef**		
		RE	SR	DR	RE	SR	DR
Acroporidae	*Acroporadigitifera* (Dana, 1846)	X			X		
	*Acroporahemprichii* (Ehrenberg, 1834)		X			X	X
	*Acropora* sp. Oken, 1815	X	X	X	X	X	X
	*Astreoporamyriophthalma* (Lamarck, 1816)					X	X
	*Montiporacrypta* Turak, DeVantier & Veron, 2000						X
	*Montiporadanae* Milne Edwards & Haime, 1851						X
	*Montiporaefflorescens* Bernard, 1897					X	X
	Montiporacf.grisea Bernard, 1897					X	
	*Montiporamaeandrina* (Ehrenberg, 1834)		X	X		X	X
	*Montipora* sp. Blainville, 1830		X			X	
	*Montiporatuberculosa* (Lamarck, 1816)	X		X	X	X	
Agariciidae	*Gardineroserisplanulata* (Dana, 1846)		X				
	*Leptoserismycetoseroides* Wells, 1954		X				
	*Pavonadiffluens* (Lamarck, 1816)			X		X	
	*Pavona* sp. Lamarck, 1801	X	X			X	
	*Pavonavarians* (Verrill, 1864)	X	X	X		X	X
Astrocoeniidae	Stylocoeniellacf.guentheri (Bassett-Smith, 1890)					X	X
Coscinaraeidae	*Coscinaraeamonile* (Forskål, 1775)			X		X	X
Dendrophylliidae	*Turbinariamesenterina* (Lamarck, 1816)						X
Euphylliidae	*Galaxeafascicularis* (Linnaeus, 1767)					X	X
	*Gyrosmiliainterrupta* (Ehrenberg, 1834)						X
Fungiidae	Ctenactiscf.crassa (Dana, 1846)		X	X		X	
Leptastreidae	*Leptastreabottae* (Milne Edwards & Haime, 1849)						X
	*Leptastreainaequalis* Klunziger, 1879					X	
	*Leptastreatransversa* Klunziger, 1879		X	X		X	X
Lobophylliidae	*Acanthastreahemprichii* (Ehrenberg, 1834)						X
	*Echinophyllia* sp. Klunzinger, 1879					X	
	*Echinophyllia* cf. sp Klunzinger, 1879			X			X
	*Oxypora* sp. Kent, 1871						X
Merulinidae	*Cyphastreachalcidicum* (Forskål, 1775)	X	X			X	X
	*Cyphastreakausti* Bouwmeester & Benzoni, 2015		X			X	X
	*Cyphastreamagna* Benzoni & Arrigoni, 2017		X		X		
	*Cyphastreamicrophthalma* (Lamarck, 1816)			X		X	X
	*Cyphastrea* sp. Milne Edwards & Haime, 1848					X	X
	*Dipsastraeadanai* (Milne Edwards & Haime, 1857)					X	
	*Dipsastraeafaviaformis* (Veron, 2000)						X
	*Dipsastraealaxa* (Klunzinger, 1879)						X
	*Dipsastraeamatthaii* (Vaughan, 1918)				X		X
	*Dipsastraeapallida* (Dana, 1846)				X	X	X
	*Dipsastraea* sp. Blainville, 1830					X	
	*Dipsastraeaspeciosa* (Dana, 1846)					X	X
	*Echinoporaforskaliana* (Milne Edwards & Haime, 1849)					X	X
	*Echinoporafruticulosa* (Ehrenberg, 1834)		X				X
	*Favitesabdita* (Ellis & Solander, 1786)				X		
	Favitescf.complanata (Ehrenberg, 1834)						X
	*Favitesrotundata* Veron, Pichon & Wijsman-Best, 1977						X
	*Favitesvasta* (Klunzinger, 1879)					X	
	*Goniastreaedwardsi* Chevalier, 1971	X	X	X	X	X	X
	*Goniastreapectinata* (Ehrenberg, 1834)			X		X	X
	*Goniastrearetiformis* (Lamarck, 1816)					X	
	*Goniastrea* sp. Milne Edwards & Haime, 1848					X	
	*Goniastreastelligera* (Dana, 1846)				X		
	*Merulinascheeri* Head, 1983						X
	*Paramontastraeaperesi* (Faure & Pichon, 1978)						X
Pachyseridae	*Pachyserisspeciosa* (Dana, 1846)					X	
Pocilloporidae	*Pocilloporadamicornis* (Linnaeus, 1758)	X	X			X	X
	*Pocillopora* sp. Lamarck, 1816	X	X	X		X	X
	*Pocilloporaverrucosa* (Ellis & Solander, 1786)	X	X	X	X	X	X
	*Seriatoporacaliendrum* Ehrenberg, 1834			X		X	X
	*Seriatoporahystrix* Dana, 1846					X	
	Stylophoracf.kuehlmanni Scheer & Pillai, 1983		X				
	*Stylophorapistillata* (Esper, 1792)		X	X	X	X	
	*Stylophora* sp. Schweigger, 1820		X	X			
	*Stylophorasubseriata* (Ehrenberg, 1834)				X		X
Poritidae	*Poritesrus* (Forskål, 1775)	X	X	X	X	X	X
	*Porites* sp. Link, 1807	X	X	X	X	X	X
Psammocoridae	*Psammocoraprofundacella* Gardiner, 1898			X			X
	*Psammocora* sp. Dana, 1846		X				

**Table 3.** Percentage Mean and Standard Deviation of Coral growth forms. Abbreviations: **reef edge** (**RE**, 1 m), **shallow reef slope** (**SR**, 4.3 m for the current-exposed site and 3.7–4.75 m for the sheltered site) and **deeper reef slope** (**DR**, 8.5–8.8 m for the current-exposed site and 7.2–9.5 m for the sheltered site).

Coral Growth Form	Sheltered Reef			Current-exposed Reef		
	RE	SR	DR	RE	SR	DR
*Acropora* Coral Branching	5.59 ± 3.43	6.14 ± 3.83	3.21 ± 3.43	4.16± 4.60	12.90 ± 9.97	16.89 ± 4.18
*Acropora* Coral Digitate	--	3.63 ± 3.96	--	--	2.30 ± 3.98	1.97 ± 3.42
Coral branching	37.70 ± 26.65	13.80 ± 4.45	4.52 ± 3.03	67.47 ± 9.35	27.30 ± 8.83	14.27 ± 7.45
Coral encrusting	1.41 ± 1.64	19.14 ± 17.25	14.38 ± 6.55	1.91 ± 1.06	21.40 ± 2.34	24.22 ± 11.63
Coral foliaceous	--	--	--	--	0.39 ± 0.68	0.33 ± 0.57
Coral massive	--	9.54 ± 14.35	72.14 ± 5.02	12.35 ± 17.75	19.18 ±13.46	27.58 ± 10.95
Coral Mushroom	--	1.82 ± 3.15	0.32 ± 0.55	--	0.42 ± 0.73	--
Coral submassive	54.02 ± 27.15	45.93 ± 25.47	5.45 ± 8.47	13.38 ± 16.59	16.11 ± 6.72	14.74 ± 10.66

**Table 4.** Percentage Mean and Standard Deviation of benthic life categories. Abbreviations: **reef edge** (**RE**, 1 m), **shallow reef slope** (**SR**, 4.3 m for the current-exposed site and 3.7–4.75 m for the sheltered site) and **deeper reef slope** (**DR**, 8.5–8.8 m for the current-exposed site and 7.2–9.5 m for the sheltered site).

Category	Sheltered Reef			Current-exposed Reef		
	RE	SR	DR	RE	SR	DR
Algae	36.13 ± 10.59	44.61 ± 15.89	45.43 ± 6.09	53.70 ± 8.85	28.65 ± 6.38	31.64 ± 17.07
Dead Scleractinian Corals	8.79 ± 8.88	10.54 ± 7.52	3.96 ± 3.90	0.18 ± 0.30	8.91 ± 4.55	4.26 ± 5.00
*Millepora*	20.86 ± 2.69	0.60 ± 1.04	0.46 ± 0.58	27.92 ± 14.05	1.57 ± 2.68	0.38 ± 0.54
Other Fauna	3.64 ± 1.83	3.93 ± 4.09	2.73 ± 1.97	0.72 ± 0.25	4.70 ± 2.14	1.63 ± 1.02
Scleractinian Corals	30.31 ± 13.79	23.35 ± 10.93	31.29 ± 1.17	17.49 ± 5.96	34.58 ± 11.11	29.53 ± 1.62
Soft Corals	0.27 ± 0.44	14.20 ± 6.39	14.27 ± 2.56	--	9.15 ± 8.05	22.92 ± 10.68
Substrate	--	2.77 ± 3.26	1.85 ± 2.83	--	15.56 ± 19.57	9.64 ± 14.51
